# Cross-sectional social network study of adolescent peer group variation in substance use and mental wellbeing

**DOI:** 10.1016/j.socnet.2023.12.002

**Published:** 2024-07

**Authors:** Srebrenka Letina, Emily Long, Paul McCrorie, Kirstin Mitchell, Claudia Zucca, Julie Riddell, Sharon Anne Simpson, Laurence Moore, Mark McCann

**Affiliations:** 1 MRC/CSO Social and Public Health Sciences Unit, School of Health & Wellbeing, University of Glasgow, Glasgow, UK; 2 Jheronimus Academy of Data Science, Tilburg University, Tilburg, Netherlands

**Keywords:** adolescents, friendship networks, peer groups, group detection methods, substance use, mental wellbeing

## Abstract

Adolescent health-related behaviours and outcomes are shaped by their peers through various social processes. Research using network data on friendship ties has uncovered evidence for processes such as peer influence and imitation. Much less is known about how the structure of small groups within a network, network communities that represents its meso level, affect individuals. The structure and composition of peer groups could play an important role in shaping health behaviour but knowledge of the effects of groups is limited.

We used data from The Peers and Levels of Stress study, a cross-sectional social network study conducted in 2006 of 22 secondary schools in Glasgow, Scotland. Students from one year group (15-16 yrs., N= 3148; 50.8% women) provided information on socio-demographics, health behaviour and friendships via a questionnaire. Dependent variables were substance use and general mental wellbeing as composite scores on different measures.

We used a series of multilevel models with students (level 1), network communities (peer groups) identified by the Walktrap algorithm (level 2), and schools (level 3). We found substantial and moderate clustering at the peer group level for substance use and mental wellbeing, respectively. Larger and more transitive groups were associated with less substance use, but worse mental wellbeing.

Addressing the methodological gap regarding the influence of the choice of group detection method on findings, we repeated our analysis using nine additional methods. The choice of the method somewhat influenced peer group variance and greatly influenced association of peer group properties with health.

This study makes two key contributions to school-health improvement research. Beyond describing the peer group clustering health outcomes, this is the first demonstration that structural and compositional characteristics of peer groups are associated with individual health, while highlighting the sensitivity of findings to group detection method used.

## Introduction

Interactions with same-age peers are an important source of influence on adolescent behaviours, attitudes, values, health risk behaviours and wellbeing (Moody et al., 2010; Long et al., 2020). Adolescents that socialize together tend to be similar in many behaviours and attitudes (e.g., see Laninga-Wijnen & Veenstra, 2021; Barnett et al., 2014). Given the significant amount of time adolescents spend in school, where they are constantly surrounded by their peers, it’s not surprising that the school environment’s role in influencing health is well-recognized (Bonell et al., 2013). Peers that belong to the same friendship group within a school may provide a different and possibly stronger source of influence than peers in another group. Peers who are members of the same group may share social norms, social identity, socialize together, influence each other’s behaviour directly or indirectly and together shape their social environment. These collective group processes could have an influence on individual health and could set the context for whether or not any health improvement intervention will have a positive effect. While it is known that young people with similar health-behaviours and outcomes cluster together (Barnett et al., 2014; Ennett & Bauman, 2000), there is a gap in understanding how properties of these groups vary and the extent to which between group variations in some properties are related to health outcomes. To date, theorizing and exploring these ‘meso level’ social processes and the effect on individuals has been underdeveloped in the adolescent health literature. In this study, we conceptualize the meso level in the context of peer groups within schools, specifically those identified using group detection methods applied to friendship nomination data. We discuss the processes behind the associations between the composition and structure of these groups and individual health outcomes.

### Meso level in studies of school networks

The importance of the school environment for health is well recognised globally as reflected in The WHO Health Promoting School framework for improving the health and wellbeing of students and their academic achievement (Langford et al., 2014), and in national recommendations to take a whole school approach to student mental health and wellbeing (Scottish Government, 2021). However, many health interventions in schools focus on the micro-level e.g., individual pupil counselling, or intervening with a pair of pupils (e.g., a bullying issue), or on the macro level i.e., whole school approaches where all pupils receive the same intervention (March et al., 2022). Better understanding of meso level processes could inform better tailoring of school health initiatives to the specific context of peer groups, where various forms of network multipliers, as described by VanderWeele and Christakis (2019), may come into effect. Since health outcomes are usually not evenly distributed across networks, but rather show community clustering (Lorant & Tranmer, 2019), this suggests that school health interventions could operate via meso level mechanisms. Defining what qualifies as a meso level in a study is dependent on its contextual application and the points of contrast, as discussed by Ylikoski (2012). In this study, we consider peer groups in schools to be the meso level. We operationalize the meso level in schools via network partitions identified by using different group detection methods based on network data. Therefore, the meso level is a level that usually goes beyond the micro-level of an individual student and extends beyond dyadic relationships and localized network structures (e.g., triads) (Robins, 2006). Hence, network theories, that is, the mechanisms that are used to explain network meso effects can differ. Yet, network partitions (or communities) have been largely underused in health research (Smith et al., 2020) leaving a gap in theory and knowledge about how their properties may affect individuals. However, this gap is increasingly recognised in social network research which is reflected in some recent methodological developments that specifically address potential differences in social processes between network communities (e.g., latent community adaptive network regression (Mathews & Volfovsky, 2021)).

### Collecting data about peer groups

Peer groups in a school are sets of pupils who may be considered as being relatively closely connected. The most central features of a group are social interactions and close relationships (Kindermann & Gest, 2009). Data on these connections between students in schools can be collected in different ways, besides self-reported friend nominations. Peer groups can be based on self-identification (so-called “social crowds”, based on shared values, beliefs, hobbies, lifestyles, etc) or other activities. They can be reported by others (teachers, other peers – e.g., socio-cognitive maps), or based on records, including on-line communication or observations of interactions (or physical proximity, e.g., Elmer & Stadtfeld, 2020) or memberships (e.g., sport clubs). All types of data collection can be used to identify groups either directly or indirectly (in case of bipartite projections) via constructing network data.

In this study we use data about friend nominations to construct networks of friendships in schools and subsequently identify their different parts representing different peer groups. When we are interested in friendship ties, characterized by a nuanced relationship quality best assessed by those within the relationship, turning to observational data or proximity-based data may not necessarily yield more valid or meaningful information and could potentially introduce additional ethical concerns and increased costs. While the lack of objectivity and informant accuracy in reporting social interactions is well-documented (Bernard et al., 1984), including subjective viewpoint may not be considered as disadvantage. In sociocentric data collection, objectivity concerns can be partially addressed by considering the reciprocity of ties. In comparison to self-identification-based data (or activities/affiliation-based data), friend nominations provide more fine-grained information on dyadic level, although the previous may be a method of choice when one is more interested in individual’s social identity then in relationship patterns. Finally, while inherently subjective, nominations of friends are based on data about individual’s friendships, and the individual is likely to be the best source of that information, in contrast with reports from teachers or other students who may have no information or accurate knowledge about the ties they are not involved in. Ideally, data collection would include more than one type of data collection, allowing that identified groups can be cross-validated with different methods, but that type of research is rarely done (for an exception, see van Woudenberg et al., 2020).

### Methods used to identify peer groups in network data

Understanding of peer group processes requires detection of groups within networks. One of the simplest ways is to identify peer group for each individual separately as those to whom one has direct contact (ego-network), but such operationalization of a group may be considered narrow when we are interested in social processes that go beyond dyad and triads and involve indirect contacts that are encompassed by mesosystems (Kindermann & Gest, 2009). In such wider group contexts, there is a possibility that the importance of dyadic exchanges taking place within them is magnified (Thibaut & Kelley, 1959). The groups identified by a group detection method (GDM) can uncover larger groups and shed light on various meso level social processes. Our ‘groups’ are defined as the sets of individuals identified by the GDMs based on data on friendship nominations within a school, and we use the terms ‘communities’ or ‘peer groups’ to refer to them. We acknowledge that GDM-derived network partitions represent one of several possible ways to identify and measure peer groups and may not perfectly align with real peer groups. Rather than adopting a strict definition, further theory building, and intervention development would benefit from diverse working definitions of meso level, group, or community. As such, we have adopted a loose definition of meso level as those patterns and processes operating above the level of individuals.

There are two main types of GDMs applicable to network data: Sociometric GDMs detect groups of individuals with a similar pattern of ties to others (e.g., similarly popular) but who are not necessarily connected with each other (Kindermann, 1993); Community Detection Algorithms GDMs aim to detect groups where ties are more common within the group than between groups in the same network. Different methods for group detection will provide different results but is not known to what extent will these differences affect related findings. Therefore, how we understand the meso level depends on the GDM used. There is no clear agreement regarding which method to use despite many large-scale studies that compare their performance, there is also a lack of data about true communities in the network (so-called “ground truth”) (Fortunato & Newman, 2022). Recent guidelines suggest that the choice of GDM should align with the purpose of the study and the properties of the algorithm underpinning the GDM (Smith et al., 2020), but there is no empirical work on how effective this approach is. The extent to which GDM choice could affect the findings of a study, or the ability of an intervention to appropriately account for meso level influences remains unknown. We address this methodological gap by assessing the influence of GDM choice on the analysis of group level variation in adolescent health in schools.

### Bundles of health behaviours

In addition to the challenge of accurately detecting groups, there is the challenge of capturing the most appropriate health outcomes. Most social network studies of peer influence on health focus on single behaviours (such as alcohol drinking, smoking, using marijuana) or constructs (depressive symptoms), with some notable exceptions (Long et al., 2017; adams et al., 2022). However, different health behaviours and outcomes are known to co-occur (Jackson et al., 2012). For example, internalising problems (negative behaviours and emotions directed inwards, for example feeling sad or anxious) and externalising problems (behaviours directed outwards, towards others in the environment, including substance use, aggressiveness, delinquent behaviour, etc.) are the two main dimensions used to describe the structure and co-occurrence of behavioural and psychological problems (Bongers et al., 2003; Leadbeater et al., 1999; Cicchetti & Toth, 1991]. Additionally, different kinds of substance use tend to co-occur (Halladay et al., 2020). For example, previous research has shown the existence of interdependence in multiple substance use among adolescents (Wang et al., 2018). Researchers increasingly recognize the possibility that mechanisms of social influence (or selection) may operate on behavioural patterns rather than single behaviours (so-called associative contagion (Golberg & Stain, 2018)), since “individuals may perceive and interpret *bundles* of behaviours of their peers, rather than simply adopt the specific behaviours of their peers” (Laninga-Wijnen & Veenstra, 2021, p.21). For this reason, we use a dimension reduction technique to identify bundles of health outcomes instead of using single outcomes. The bundles of health behaviours may be a more appropriate type of outcome to study at meso level, as we cannot assume that indirect contacts will have information on individual’s single behaviours, but they may have an idea about their general pattern of behaviours.

### Social processes in peer groups

A peer group can have a role in the development and maintenance of health-related behaviours of individuals through a range of complex, simultaneous, dynamic, and potentially interdependent mechanisms. For example, direct and indirect exposure to the behaviour can provide an opportunity to engage in certain behaviours, via peer pressure, imitation, selection and de-selection of friends, or conformity to shared norms. Some of the most examined in the literature are: selection (or homophily – tendency to form connections with similar others) and deselection, contagion (or social influence, e.g., peer pressure, describes tendency for individuals’ behaviours or attributes to be influenced by the behaviours or attributes of their social contacts), imitation and norm conformity. These processes, if present for a certain health outcome, will lead to social clustering within school according to that health outcome, in addition to some contextual factors. In social network literature it is well-recognized that selection and contagion are often difficult to disentangle and determine which of them is causing similarity based on cross-sectional data (e.g., Shalizi & Thomas, 2011). Nonetheless, that is not as serious issue when we are interested only in qualifying the level of similarity of members of the same group, irrespective of its causes. In that case the fact that adolescents cluster together based on other attributes, e.g., gender (McPherson et al., 2011; Goodreau et al, 2009), socio-economic status (Block & Grund, 2014; Chabot, 2021), ethnicity (González et al., 2005; Leszczensky & Pink, 2015) that themselves can be related with higher risk for certain unhealthy behaviours and health outcomes, still poses a challenge. Some of such traits may be included in the analysis and controlled for, but it would be difficult to claim that some unmeasured attributes may also be of relevance.

### Meso level properties and individual health outcomes

Many studies in recent decades have found that adolescents and young adults with similar health-behaviours and outcomes cluster together (Ennett & Bauman, 2000; Barnett et al., 2014). Yet, while the research has been limited to an assessment of degree of clustering of health-related outcomes within identified network communities, another crucial aspect of meso level research is to assess whether individual differences in health outcomes are associated with characteristics of the identified network partitions to which they belong to.

The school network is partitioned in several smaller parts of the original network. These communities themselves can be viewed as small(er) networks and are likely to differ in their network properties such as size, density, etc. We hypothesize that these global features of communities can be relevant for health outcomes of their members, independently or in interaction with social processes (e.g., contagion, selection) that arise from the direct or indirect (e.g., conformity, imitation) contact with other community members. Yet, theorizing about this network’s meso level and its effects on individuals has been underdeveloped in health research of adolescents. But research on whole network and ego networks may provide some insights. The idea that network properties at macro level are associated with individual wellbeing can be found in the classical work of Durkheim (1952) and has been applied and supported in studies of school networks (e.g., Gauthier et al., 2022). Numerous studies of whole school networks employing statistical models for network data (e.g., exponential random graph models, stochastic actor oriented models) which usually look at relatively smaller network structures (dyads, triads, etc.) to investigate the existence of network effects on macro level, also found support of social selection, influence, contagion (and convergence) for many health-related outcomes (e.g., for smoking see Mercken et al. (2012) and a review on smoking and social networks in schools see by Littlecott et al. (2022); for depression see Kiuru at al. (2012) and Van Zalk et al. (2020); for a review see Montgomery et al. (2020)). Research on ego-networks of adolescents and health outcomes (e.g., Laken et al. (2010) study smoking, but their research design also investigates school macro level) also could inform potential hypotheses about meso level, but to a limited extent. The findings from whole school networks and ego networks could imply that health outcomes tend to cluster within network meso-level structures, allowing for predictions about some group properties and individual outcomes. Nonetheless, it’s important to consider that social processes at the meso level may differ from those at the macro (whole network) level. Additionally, ego networks do not encompass indirect contacts, which can lead to substantial differences in findings. Most importantly, these studies do not directly investigate the network meso level.

We assume that those community properties will be associated with individual health outcomes via bidirectional effects. Individuals with certain health outcomes may have tendencies to form patterns of connections around them that lead to specific community properties or may have tendency to join groups with certain properties. E.g., individuals with high on measures that can be considered as proxy of wellbeing (emotional stability) may tend to create more transitive relationships around them as some personal network research^1^ suggests (Kalish & Robins; 2006, Maya-Jariego et al., 2020) and in that way contribute to making the community they are in more transitive.

The effects may be direct or mediated by other contextual or individual variables, and social processes on network macro level. Community properties may affect individual health outcomes of individuals in two ways simultaneously: via diffusion potential and via social capital associated with the property. Some community properties may facilitate the diffusion of health-related behaviours, e.g., in more transitive and small communities, the spread of specific health behaviours is faster than in less transitive and bigger communities (Centola & Macy, 2007). But the same property may be associated to social capital and as such affect individual health behaviours, e.g., a higher transitivity is associated to higher social integration and higher wellbeing (Wray et al., 2011).

On micro level, other individual attributes can affect how individual forms ties with others and with who (e.g., gender, some personality traits) and in that way can affect community properties. Individual attributes can have their own independent effect on individual health behaviours. It should not be surprising that our general framework is parallel to the micro-macro approach developed in analytical sociology (Coleman, 1987). The difference is that the “macro” level in the context of our study is the network’s meso level, while allowing for the macro level (school in our context) effects also to be incorporated into analysis.

The challenge in forming and testing theories about property of meso level comes from the fact that is not not possible to disentangle the effect of micro level on meso level and vice versa with cross sectional data. This is additionally complicated when macro level is also included. But even longitudinal data may not easily enable separating meso property effects related to diffusion potential and social capital.

**Fig 1:**
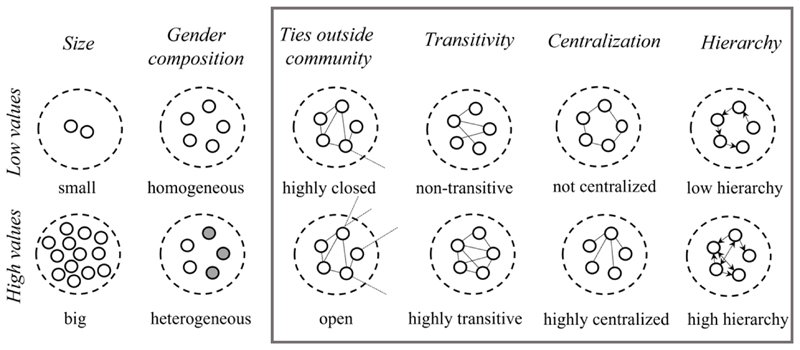
Six community properties investigated in this study. Two properties are compositional (left) and four are structural (framed right). The figure shows toy examples of communities manifesting low or high level of each property.

In this study, we focus on six properties of groups that could be associated with health, shown in figure 1. Two properties are compositional and four are structural.

The selection of properties under investigation was influenced, in part, by the choice of network measures that exhibit limited interdependence and describe fundamental characteristics of a community, such as size, the ratio of outside community ties, and transitivity. Additionally, we incorporated properties deemed generally significant by prior research (e.g., McFarland et al., 2014) focusing on adolescents’ networks within school settings. Among the six measures outlined in Figure 1, four can only be computed using network data. We will proceed to provide a detailed description of each measure and elucidate its relevance within the context of our study and tentatively suggest the possible direction of their associations with individual health outcomes.

#### Community size

The most easily noticeable difference between communities is their size – the number of students belonging to the community. Smaller communities are often considered “cliques,” even though they may not strictly adhere to the network definition of a clique. In such communities, members tend to have very close relationships and frequent communication. Smaller groups may suggest closer relationships with more frequent communication, but greater constraints due to social pressure. Larger communities may comprise several “cliques” and exhibit a different network structure than smaller groups. This is because they involve both close ties with some members and weak ties with others (Cotterell, 1996). Adolescents in bigger groups may feel less pressure to conform to a specific group identity or set of norms, allowing for greater individuality, although they may engage in social comparison more frequently. In larger communities, direct and close relationships may hold higher significance than in small groups (Giordano, 1995). Bigger groups may consist of close ties with some members and weak ties with others (Cotterell, 1997). They offer their members more freedom and diverse interaction but also introduce more uncertainty (Simmel, 1950) due to less knowledge about one another. While most members may interact, it does not necessarily mean they all like each other. Small and big communities may have different social dynamics, but also differ in their relevance to an individual. It’s important to note that adolescents may belong to both small and large peer groups simultaneously and the two are likely to overlap (small group of close relationships within a larger group).

#### Community gender composition

People of all age groups, including adolescents, tend to form friendships with similar individuals, a concept known as homophily. One of the most prominent factors influencing homophily is gender (Smetana et al., 2006; Shrum et al., 1988).

Friendships in adolescence typically begin within same-sex pairs and later expand to include opposite-sex friends (Cotterell, 1996). In case of high gender clustering many communities will have only girls or boys as members. Therefore, each community can be described as female, male, or mixed – if it had at least one member of the opposite gender. Stolle et al. (2008) suggested that communities with compositional heterogeneity create an uncertain context that raises issues concerning trust and group boundaries. A review of gender disparities in friendships (Rose & Rudolph, 2006) reveals findings that, while not always consistent, indicate that friendships among girls tend to demonstrate greater levels of intimacy, support, self-disclosure, co-rumination, and dyadic interactions compared to friendships among boys. Boys, on the other hand, often engage in more competitive friendships, characterized by hierarchies and activities such as sports, which often involve more than two individuals. It is reasonable to assume that these differences could potentially influence variations in health-related behaviours.

#### Ratio of outside community ties

Communities can be more or less “strong” or closed, where their members have most of their ties with each other, as opposed to “weak” or open communities where members have most of their ties with individuals from other communities. A strong community is characterized by a low ratio of outside community ties, while the opposite holds true for weak communities. An open community may receive more information on a diversity of norms and behaviours from other groups, while a closed may exhibit more self-reinforcement of group norms. This measure seems especially appropriate in the context of using a group detection method to identify communities, because it provides information about how delineated each community is from others and therefore how potentially it is open or closed to influences from other communities. Closed communities lack access to peers outside the group, which may result in limited information and fewer suitable references for different behaviours. Conversely, very open groups may lack distinct identity and a sense of belonging. In both scenarios, such groups can be a conducive environment for the development and maintenance of negative health behaviours. Consequently, we would anticipate diminishing returns on centralization in relation to positive health outcomes.

Transitivity – Besides its size and density, one of the most fundamental network properties is transitivity, sometimes referred to as clustering. A community is considered transitive if there is a tendency for a friend of a friend to be a friend. Transitive ties are linked to strong ties, and transitive networks are associated with higher levels of ‘social capital,’ which include elements such as trust (Buskens, 1998), norms, obligations, and information disclosure (Nahapiet & Ghoshal, 1998). Such characteristics may create environment in which positive health behaviours are encouraged and maintained. But the same property can be associated to increased control, faster information flow, and the spread of behaviours among members (Centola & Macy, 2007). The latter implies that the property’s effect will be partly contingent on how many members manifest positive and negative health behaviours.

#### Centralization

This property describes how uneven the distribution of ties in the group is and therefore it partially captures the differences in status. If everyone had a similar number of ties with others, centralization would be close to zero, implying no status differentiation in the group. Most people have a desire for status compared to others, and this desire is amplified during adolescence (Veenstra & Laninga-Wijnen, 2021). However, a very high centralization can indicate that one or a few individuals are most important in the group, and such uneven status distribution may be conducive to negative health behaviours not only for others, but also for those central individuals. Alternatively, it is possible that at risk individuals are more likely to be part of such groups. Because of their centrality, their social influence may be stronger, and their health-related behaviours may set the group norms. Hence, even if more centralized communities do not necessarily foster a healthy social environment, if the “leader” sets a positive example or norms of positive health behaviours, this may encourage positive health behaviours in the group, at least those that are observable to others.

#### Hierarchy

Centralization does not account for the status ordering provided by the direction of ties, which provides more information about the hierarchy. “Pecking orders” tend to emerge within a network (Redhead & Power, 2022; Michell & Amos, 1997), and some asymmetry in relationships is often preferred (McFarland et al., 2014). Therefore, we also employ a more complex measure of hierarchy (or social dominance) that utilizes information about the direction of ties among all triads (a subgraph of three nodes) in the community (further details in Supplementary Materials, section 4). In regard to individual health outcomes, it is possible that being in a community with a high hierarchy is unpleasant and can lead to negative health-related behaviours, or alternatively that vulnerable individuals are more likely to be part of such communities.

To conclude, in addition to homophily and contagion and other social processes, the community properties, and other processes associated with meso level, may amplify the similarity of group members.

### Research questions and hypotheses

The aim of the study was both theoretical and methodological. We sought to examine the extent of peer group variation in adolescent health outcomes, the association between group characteristics (community properties) and health, and the effect of group detection method on the ability to examine peer group variation.

We frame our research around three research questions, among which the first two are substantive and the third is methodological. The questions and their hypothesis are followed by our rationale and more detailed tentative expectations.

#### RQ1. To what extent do substance use and mental wellbeing vary at the peer group level?

H1: Peer groups in schools vary regarding the health-related outcomes of their members, even after controlling for some individual and school level characteristics.

Some individual factors (micro level) may affect peer group membership, health outcomes, or both. Specifically, we control for gender, ethnicity, socioeconomic status, and parental behaviours. We expect that even when these factors are considered, variation in health outcomes between peer groups will still be present.

In addressing this and other research questions, we avoid distinguishing between different health outcomes, not because there are not theoretical grounds to do so, but because it falls outside the scope of our present work. Our focus centres on the meso level and health outcomes from a broad perspective, rather than delving into specific variations in meso level effects on different health outcomes.

#### RQ2. Are some structural and compositional properties of communities (peer groups) related to health outcomes of individuals?

H2: Peer group characteristics (e.g., its size) are associated with individual health outcomes.

As there is, to the best of our knowledge, no existing theoretical framework or direct research addressing RQ2, our approach to answering this question is exploratory in nature and that is reflected in broad and non-specific hypothesis.

We anticipate that some community properties may have associations with individual health outcomes and form some less broad tentative expectations. For example, we might expect that a higher transitivity within communities is linked to positive individual health outcomes, while greater centralization and hierarchy within communities may be associated with negative health outcomes. These expectations, however, are contingent on several factors, such as whether individuals with negative health outcomes tend to join or form such communities. Additionally, the predictions hinge on the health behaviours and outcomes of community members; for instance, individuals with negative health behaviours within highly transitive communities may influence faster spread of such behaviours. If central members in centralized community and high-status members in highly hierarchical community manifest positive health behaviours, they may influence others to have positive health outcomes.

Community size and the ratio of ties outside the community can be hypothesized to positively impact individual health outcomes. While it is intuitive to suggest that very small or closed communities may potentially lead to negative health outcomes due to social isolation, it is also conceivable that individuals with negative outcomes may be more inclined to be part of such communities (due to lack of capacity, motivation, or opportunity to be part of a big or a more open community). Nonetheless, overly open or large communities may not necessarily guarantee better health outcomes, as the former might lack closeness and certainty, and the latter could be unstable. Hence, we may not expect that being in such groups is necessary associated with positive health outcomes, suggesting the possibility of diminishing returns of those effects in relation to positive health outcomes.

In the case of community gender composition, its relevance is informed by the existing literature on gender gap in adolescent health outcomes. Girls on average tend to have worse mental health outcomes (Campbell et al., 2021), while incidence of drug use, alcohol dependence, and smoking is greater in boys (Kloos et al., 2019). These differences lead to initial expectation that being in a one-gender community is associated with worse health outcomes compared to mixed communities. Nonetheless, if we control for gender-related differences in health outcomes among adolescents by holding the gender variable constant, we may not anticipate an independent effect of gender composition on health outcomes, unless some differences in female and male friendships in peer groups or other gender differences do not have effect on health behaviours.

#### RQ3. How sensitive is the study of the meso level in schools to the methodological approach (method used for group detection)?

H3: Evidence for between peer group variation in health, and the association of group characteristics with health outcomes will vary depending on the choice of GDM.

While we expect variations between GDMs, we refrain from specifying more precisely which GDMs partition school networks into groups which are most different in terms of health outcomes, due to following considerations:

##### We focus on sensitivity, not differences between GDMs

Aim of RQ3 is to assess the sensitivity of our findings to the choice of GDMs, not to delve into the nuances of differences between various GDMs and what underlies them. We aim to determine how different GDMs may affect our results, rather than drawing direct comparisons between GDMs. This focus aligns with the research goals of our study.

##### Complex categorization of GDMs

The field of community detection algorithms has a rich and diverse landscape of methods. There is extensive literature dealing with classification of GDMs based on various dimensions, especially for community detection algorithms (e.g., Souravlas et al., 2021; Coscia et al., 2011). For example, Smith et al., (2020) use categorization of GDMs to divisive, agglomerative, and optimization-based methods. While they provide valuable insights into the diversity of GDMs the same categorization may not be useful for our research goals. Additionally, categorizations of community detection algorithms are multifaceted, may overlap, not fully capture the nuances of GDMs relevant for specific research goal and their accuracy my vary depending on software-specific implementation of GDMs.^2^

##### Challenges in determining “True” partition quality

Using similarity of community members in health outcomes as an indicator of the “true” partition quality of a GDM can be problematic. Social processes beyond health outcomes, such as selection, contagion, on many attributes, operate at the meso level. Therefore, community partitions associated with the most homogeneous health groups do not necessarily imply that a GDM uncovers partitions closest to the ground truth. Instead, we can expect certain structural properties like modularity and community size to differ among GDMs.

##### Modularity

We can anticipate that GDMs based on blockmodeling may reveal less interconnected communities compared to GDMs founded on modularity optimization techniques like Louvain and Walktrap. The latter may unveil peer groups characterized by close and direct interactions, potentially resulting in higher similarity in health outcomes. However, this expectation hinges on the assumption that direct social processes are more influential than indirect processes like imitation and conformity.

##### Community Size

Clique-based algorithms and those considering local network structure (e.g., Walktrap, Infomap, Label Propagation) may yield smaller communities. In contrast, GDMs like Edge-betweenness, which progressively removes edges with highest betweenness centrality which is a measure that considers the global network structure (Newman & Girvan, 2004), can result in larger communities. The size of communities can impact homogeneity based on an attribute, as larger communities are less likely to be homogeneous.

In conclusion, RQ3 aims to examine the sensitivity of results to different GDMs, given the complexity and context-dependency of these methods. We acknowledge there are differences between GDMs but emphasize that our focus is on understanding the impact of choosing different GDMs on variations of final findings of analyses that are based on them, rather than detailing specific hypotheses for each method or group of methods.

## Methods

### Data and participants

We conducted secondary analysis of data from the “Peers and Levels of Stress” (“PaLS”) study, which investigated the relationship between pupils’ peer group status and level of stress. The cross-sectional data were collected from January to May 2006 in 22 secondary schools in and around the city of Glasgow, Scotland. The schools were in socioeconomically mixed and mainly urban areas (Sweeting et al., 2008). Ethical approval for the study was given by the University of Glasgow Social Science Ethics Committee (SSL/05/03), and informed consent was provided by students and their parents. In each school, all students from one year group (fourth year of secondary school, 15-16 years old) were invited to participate. Dataset was anonymised after data collection and authors had no access to information that could identify individual participants. At the time of data collection, most of the students had attended the school for four years. Overall, the response rate was 81% (N= 3148), and 50.8% participants were girls.

### Measures

We used individual socio-demographic data and family-related measures as independent variables (level 1 covariates), their health outcomes as dependent variables, and measures of communities (level 2 covariates) based on network data. Level 3 covariates were related with school level and included school size, modularity, and proportion of female students in the school.

#### Socio-demographics

Participants were asked to provide information about their gender (male or female), their year of birth, school year, and their ethnicity.

**Family and health-related measures** are described in Table 1 (for descriptive data, see Table S2 in Supplementary Materials). The sample consisted of predominantly white students aged 15 and 16 years (10 were 17 years old). Family affluence of most students was medium or high, while 16% had low family affluence (see Table 2).

#### Outcome measures: Principal component analysis

Principal component analysis (PCA) was performed on seven health-related variables: smoking, drinking, using drugs, drug effects, self-esteem, general mental health and worries. PCA reduces data dimensionality while effectively capturing the maximum variance. This process yields principal components that are linear combinations of the original variables. Unlike factor scores or latent factors, they are often more straightforward to interpret and, in contrast to raw composite scores, are orthogonal. PCA resulted in two components with Eigenvalues higher than 1, which explained 36% (PC1) and 24% (PC2) of the variance in the measures. Horn’s parallel analysis on 500 simulated datasets confirmed the retention of two components. The first component (PC1) captures substance use (SU) behaviours (smoking, drinking, using drugs, and drug effects), while the second component (PC2) captures mental wellbeing (MW; self-esteem, general mental health and worries). For more details, see Supplementary Materials, section 2.

#### Friendship networks

A sociocentric approach was used to collect data on friendships. The students were asked to nominate up to six individuals they considered friends within their school and year class, resulting in directed networks. When the name generator is limited to only six individuals, there is a risk of excluding weak ties from the network. This limitation can have implications for community detection and the ratio of ties within the same group to those outside of it. However, it’s important to note that weak ties, as theorized by Granovetter (1973), typically represent connections to individuals who are not considered part of an individual’s core network. These weak ties serve as conduits to new information and resources and are more likely to be associated with other social groups. Therefore, the absence of weak ties is more likely to impact the connections between communities by potentially underestimating their extent, rather than significantly affecting the density of connections within individual communities.

**Fig 2:**
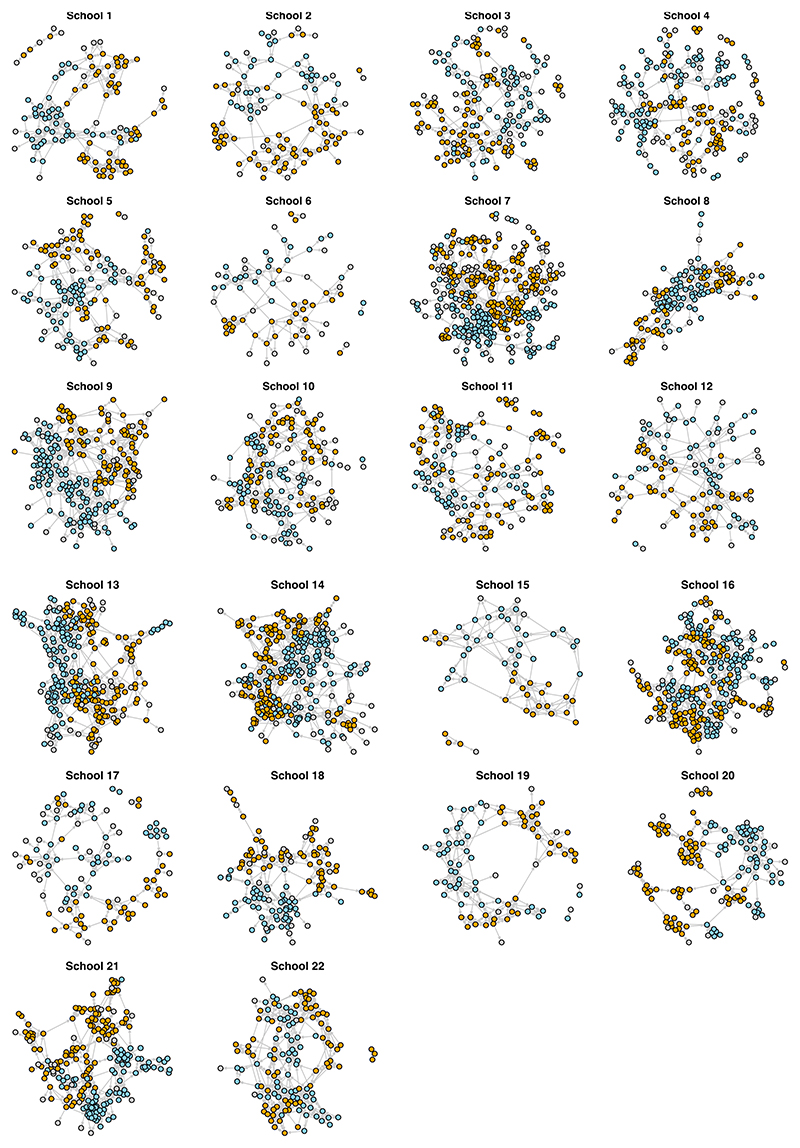
Friendship networks in 22 schools. Colour of nodes in networks: orange – young women; blue – young men, grey – no data on gender.

Some non-participating students (*N* = 501) were nominated as friends by others, and were consequently included in network analysis and group detection methods, follow. However, we encountered missing data concerning attributes and outgoing ties for these non-participating students. Notably, imputations of attribute data were exclusively performed for participating students. Figure 2 illustrates 22 friendship networks for each school, with additional network properties detailed in the Supplementary Materials (section 3).

The number of study non-participants in the network varies between schools, from only 4% to 29%. Relatively high transitivity (33% to 51%) shows a tendency for social closure, that is if A and B are both friends of C, they are likely to be friends with each other. High values of EI (external-internal) index indicate that most friendship ties (dyadic level) are between students of the same gender and the same ethnicity (see Table S5 in Supplementary Materials). The tendency for same gender friends can be seen in Fig 1.

### Analytical strategy

The friendship network of each school was partitioned into communities using ten GDMs (for RQ3) in order to test sensitivity of findings to GDM choice (RQ3). Eight GDMs were community detection algorithms (Clique percolation (CP), Edge-betweenness (EB), Fast greedy (FG), Infomap (IM), Leiden (LE), Louvain (LO), Label propagation (LP), and Walktrap (WT)) and two blockmodeling approaches: blockmodeling with indirect approach (BIA) and Stochastic blockmodeling (SBM). The criteria for the inclusion of a GDM was their availability in R software and their applicability to the networks under study. For example, some GDMs are applicable only to connected networks (e.g., Spinglass) – networks that do not have two or more members (not including the isolates) which are not connected with anybody else. For more details, see section 6 and Table S13 in Supplementary Materials containing a short description of each GDM.

We employed Walktrap algorithm (Pons & Latapy, 2005) to provide demonstrative answers to the first two research questions. The choice of Walktrap was based on several reasons. Firstly, it takes into account the directionality of connections in the network. Secondly, while other methods in our ensemble, such as Edge-betweenness^3^ and Infomap, and two blockmodeling-based approaches also allow directed networks as inputs, Walktrap stands out by utilizing random walks to identify nodes frequently visited together. This implies their shared membership in the same community. Walktrap, as implemented in igraph package (Csardi & Nepusz, 2006), in difference to other four previously mentioned GDMs, also optimizes modularity by identifying the cut point in the resulting dendrogram with the highest modularity value. This feature ensures that the partitions it uncovers have more internal connections and fewer connections between them. This aligns closely with traditional notion of peer groups as cohesive and distinct groups (Freeman, 1992, as cited in Moody & Mucha, 2023, p. 382) and makes Walktrap a suitable choice within our broader analytical framework.

Six community properties were calculated for each group and included as level 2 covariates in multilevel models (MLMs). We group the community properties in two groups: *compositional* properties that include group size^4^ and composition regarding gender; and *structural* properties that include four measures based on network data (see Fig 2 for details). Each measure of peer groups is explained in more detail in Supplementary Materials (section 4). To investigate the clustering of health outcomes within peer groups and associations of community properties with health outcomes (RQ1 and RQ2), the main analysis included a series of multilevel models (MLMs) for the two outcomes separately, based on groups identified using the Walktrap algorithm. We used a bottom-up approach, starting with the models containing only random effects: group level variation (Model 1); and including school variation (Model 1.1). A likelihood ratio test comparing the two models indicated that adding school as a random effect did not significantly improve the model fit for either of the outcomes. Thus, the effect was not included in further models. We introduced individual (Model 2), peer group (Model 3), and school (Model 4) fixed effects covariates in subsequent models. Model 4, in addition to network (school) size and proportion of female students included GDM-specific modularity scores for each school, to check whether having more delineated peer groups in schools (higher modularity) was associated with health outcomes. Due to high and positive correlation between modularity score and school size, we assigned value 1 for schools with less than 141 students, value 2 for schools with 141 to 219 students, and value 3 for schools with 220 or more students.

Furthermore, we have conducted additional analyses to address several crucial points that require consideration. Firstly, the network measures are inherently interdependent. Models 3 and 4 analyse different community properties simultaneously and these properties are not isolated from one another. For instance, larger communities often have lower transitivity. Consequently, attempting to disentangle and isolate their individual effects can be challenging. The inclusion of one network measure has the potential to influence the direction and significance of another, rendering interpretation complex. Secondly, it is important to recognize that regression coefficients are inherently conditional, and as such, their interpretation should not be construed as direct ‘effects.’ Instead, they serve as indicators of associations, adjusted for the influence of other variables incorporated into the model (as noted by Westreich and Greenland (2013)). Thirdly, we acknowledge the utility of *p*-values in guiding our interpretations. However, there are underlying assumptions associated with their use. These assumptions encompass the absence of measurement error, non-informative missing data, and conditional independence between network properties and the outcomes, predicated on the provided covariates. These assumptions, especially the last, are notably challenging due to the inherent interdependence of network measures. Lastly, we cannot assume that the associations between community properties and individual outcomes are necessarily best described as linear. In light of these considerations, while we employ *p*-values as part of our analysis, we also recognize the need for a more comprehensive descriptive analysis and additional models. To complement the multilevel models 3 to 4, we analysed relationships between each community property separately and each outcome in three ways: 1) We explored the bivariate unadjusted relationship between each community property and each outcome (using Spearman correlations, reported in supplementary-material Materials); 2) We included models that included each community property individually and random effects of communities (covariates-unadjusted model); and 3) We investigated the existence of non-linear association by adding the quadratic term for the community property to its linear term and random effects of communities (excluding gender composition which was a categorical variable). This comprehensive set of analyses aligns with our descriptive goals and accounts for the multifaceted challenges posed by the interrelated network properties. It allows for a more nuanced and comprehensive exploration of the data, which can be instrumental in providing a clearer perspective on the associations under investigation.

We conducted multilevel modelling using R packages lme4/lmer (Bates et al., 2014) (R version 4.1.0). For students who participated in the study missing attribute data was imputed with multiple imputation by chained equations (40 iterations, mice package (Van Buuren & Groothuis-Oudshoorn, 2011)). Due to complexity of our analytical strategy and relatively low percentages of missing data (see figure S1 in Supplementary Material), we used a single imputation when imputing missing values. For both dependent variables, Substance Use and Mental Wellbeing, we estimated identical linear mixed-models, using the restricted maximum likelihood method (REML) and Nelder-Mead optimizer (see Supplementary Materials, section 5 for more details of model fit). Tests of non-liner relationship and sensitivity analyses and robustness checks appear in Supplementary Materials (table S20 and section 7, respectively).

To answer RQ3, Model 3 findings were compared across all ten GDMs, supplemented by models with each community property and random effects of communities (six models per GDM). We visually inspect and report on how the intraclass correlation coefficient (ICC) and fixed effect parameters varied across the GDMs. We report all results in full and apply a correction for multiple testing to the reported coefficients for Model 3 (in Supplementary Materials, tables S21 and S22).

## Results

**Fig 3:**
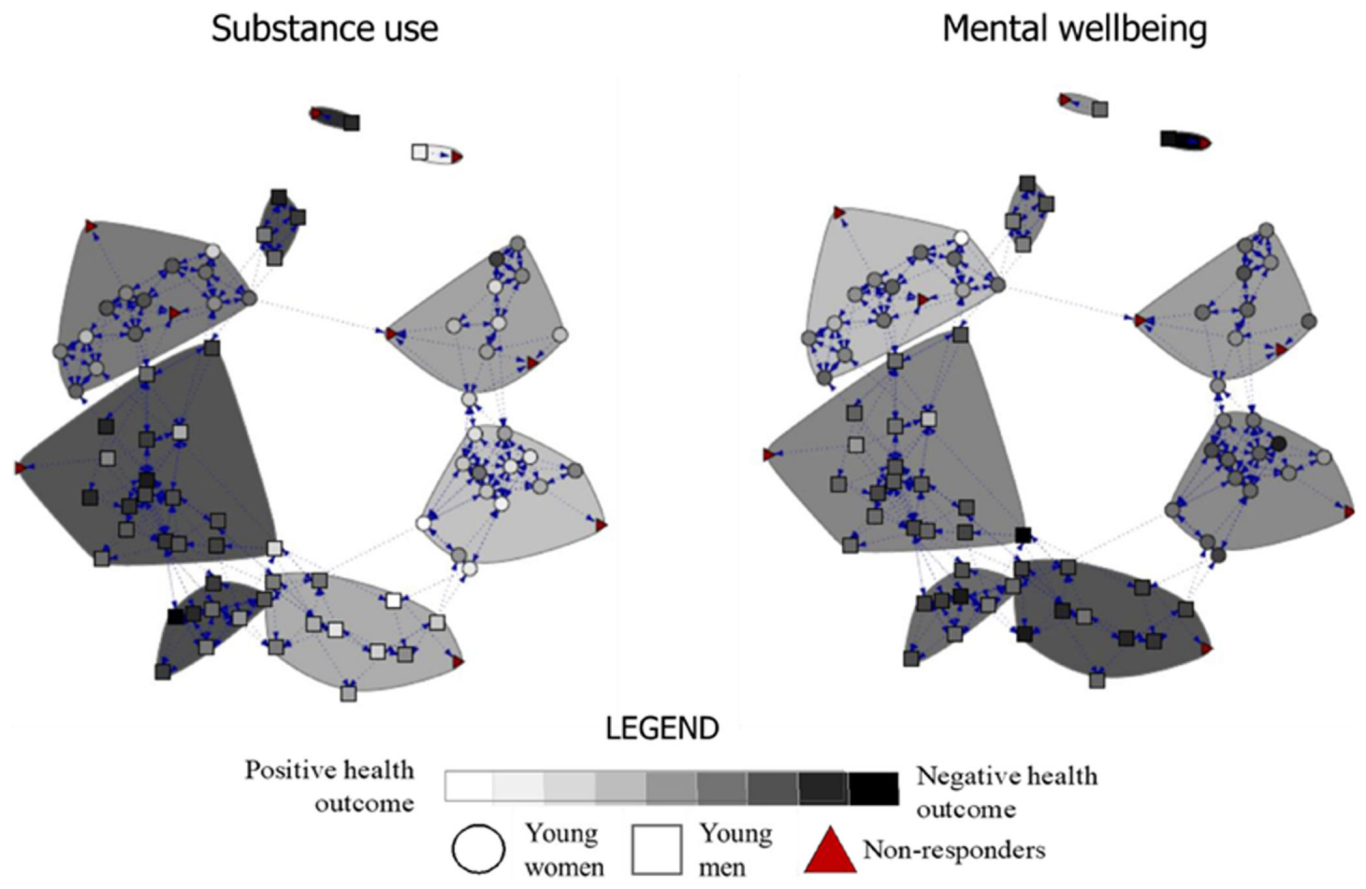
Communities and health outcomes in one school. The same school (school 19) with nine communities identified with the Walktrap algorithm is shown on left panel with Substance use outcomes per individual, and on right panel with Mental wellbeing individual outcomes. Similar shade of grey squares (young men) and circles (young women) within the same community (peer group shown in different colours) suggests that clustering regarding the outcome exists for the group. The shade of grey in the background of each community corresponds to its average level of the health outcome.

Fig 3 shows the distribution of substance use and mental wellbeing scores over communities for one school (school “19” is showed due to its relatively higher clarity because of the school size, the plots for each school are shown in section 8 in Supplementary Materials). Fig 3 suggests grouping of health outcomes by community, more so for substance use than mental wellbeing.

### Multilevel models

Model 3 was the best fitting model for both outcomes. Due to the lack of evidence for between school variation (level 3), model 3 is considered the main model in the following text and ICC only for communities’ random effects are shown.

The complete summary of results of all multilevel models for Substance Use and Mental Wellbeing, using the Walktrap algorithm, are presented in Tables 3 and 4, respectively. Higher principal components scores in Substance Use and Mental Wellbeing signify more positive outcomes, less substance use and better mental wellbeing, respectively^5^. We start with a short overview of the effects of individual covariates, thereafter we focus on level 2 covariates and measures of clustering (ICC values) that are in focus of our research questions.

### Effects of individual variables (level 1)

#### Substance use

Model 3’s total explanatory power is substantial (including both random and fixed effects, so-called conditional *R*^2^ = 0.41) and the part related only to the variables that are constant across individuals (fixed effects, marginal *R*^2^) is 0.14. Students who were female, older, ethnic majority (white), and had more controlling parents and lower parental care reported higher Substance Use (lower score).

#### Mental wellbeing

Model 3’s total explanatory power is moderate (conditional *R*^2^ = 0.26) and the part related fixed effects alone (marginal *R*^2^) is 0.15. Gender was significantly associated with Mental Wellbeing, suggesting that in this study, boys had better Mental Wellbeing than girls. The effects of parents’ behaviours are both significant. Similarly, as with Substance Use, results imply that higher control from parents is associated with worse outcomes, while higher care is associated with a better outcome. The effect of ethnicity ceased to be significant when community properties were added to the model (M3).

### Clustering of health outcomes (RQ1)

**Fig 4:**
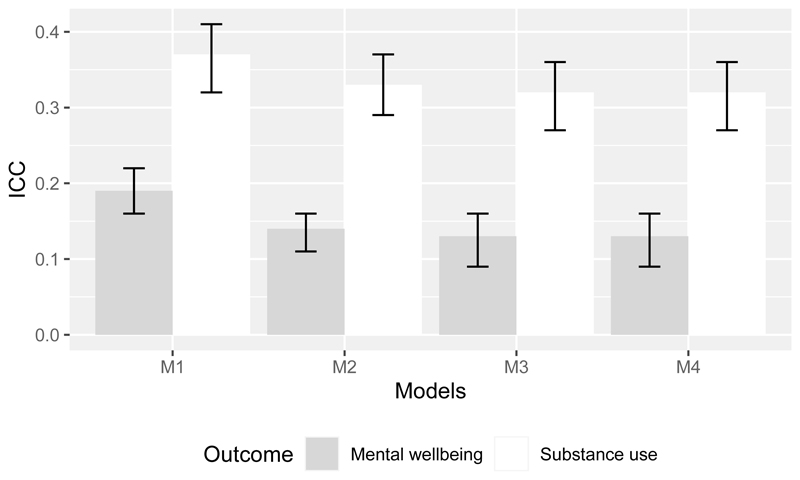
Between peer-group Adjusted Intraclass Correlation Coefficients for two health outcomes across four models. Lower limits of confidence intervals are at 2.5 percentile, upper limits of confidence intervals are at 97.5 percentile.** Group detection methods abbreviations: blockmodeling with indirect approach – BIA, Clique percolation – CP, Edge-betweenness – EB, Fast greedy – FG, Infomap – IM, Leiden – LE, Louvain – LO, Label propagation – LP, stochastic blockmodeling – SBM, Walktrap – WT.

To answer the RQ1, the main statistics of interest are intraclass correlation coefficients (ICC) which can be interpreted as a proportion of the total variance explained by between peer group rather than between-individual variation. Since we are interested primarily in random effects, we used the adjusted rather than conditional ICC (Nakagawa et al., 2017). Adjusted ICC values and their confidence intervals for the two outcomes and models 1 – 4 are shown in Fig 4.

ICC values for Model 1 show substantial peer group clustering for Substance Use (ICC = 0.37), and a notable but smaller clustering in Mental Wellbeing (0.19). 37% and 19% of the total variation in Substance Use and Mental Wellbeing, respectively, is accounted for by variations between peer groups. Accounting for individual covariates (Model 2) resulted in decreasing ICC for both Substance Use (0.33) and Mental Wellbeing (0.14). This indicates that the composition of the peer groups played a similar role in explaining clustering for both Substance Use and Mental Wellbeing. Including all community properties as level 2 covariates (Model 3), ICCs further decrease (0.22 and 0.13 respectively). In Model 4 ICCs for both outcomes do not change after including school level covariates.

### Effects of community properties on health outcomes (RQ2)

We examined six different structural and compositional properties of communities (peer groups) to see how these related to health outcomes of individuals. To answer RQ2, we use estimates of the relationship adjusted only for random effects of communities and Model 3 that included level 1 (individual covariates and all six community properties simultaneously.

#### Substance use

The estimate of community size was statistically significant and positive in Model 3, suggesting that being in a bigger community is related to lower substance use. However, the estimate in the model adjusted only for community random effects, does not suggest significant association of community size and Substance Use. According to both models, being in a more connected peer group (higher transitivity) was associated with lower substance use. How “strong” a community is (the ratio of ties outside the community) and its level of hierarchy was not associated with Substance Use. Being in an only-boys community versus being in only-girls community was associated with less substance use (higher score), but only when individual covariates and other community properties were controlled for.

#### Mental wellbeing

The effects of community size and transitivity were statistically significant and negative, meaning that being in a bigger community and being in a more transitive (more connected) community, was related with worse Mental Wellbeing. Centralization was negatively associated with Mental Wellbeing, showing a tendency of adolescents in more centralized groups to have worse mental wellbeing. There was no evidence of these associations in the models with only community size and random effects. The opposite is the case with ratio of ties outside community, where the estimate in random effects only model suggests that more open communities are associated with better mental wellbeing. In the same model, being in male or mixed community versus female community was associated with better mental wellbeing, but when covariates are included, the associations ceased to be significant. According to both models, hierarchy was not associated with Mental Wellbeing.

Model 4 in which level 3 covariates were incorporated did not show significant effects for any school level covariates for both outcomes.

Multilevel models that included random effects of communities, linear and quadratic term for each community property separately showed that the relationship between centralization and Mental Wellbeing may not be best described as linear (linear term: *p*<0.1; quadratic term: *p*<0.08).

### Sensitivity of findings to group detection methods (RQ3)

The basic description of community structure – the number of communities, their sizes, and modularity score for each GDM is provided in Table S15 in Supplementary Materials.

### Sensitivity of clustering of health-outcomes to group detection method

**Fig 5:**
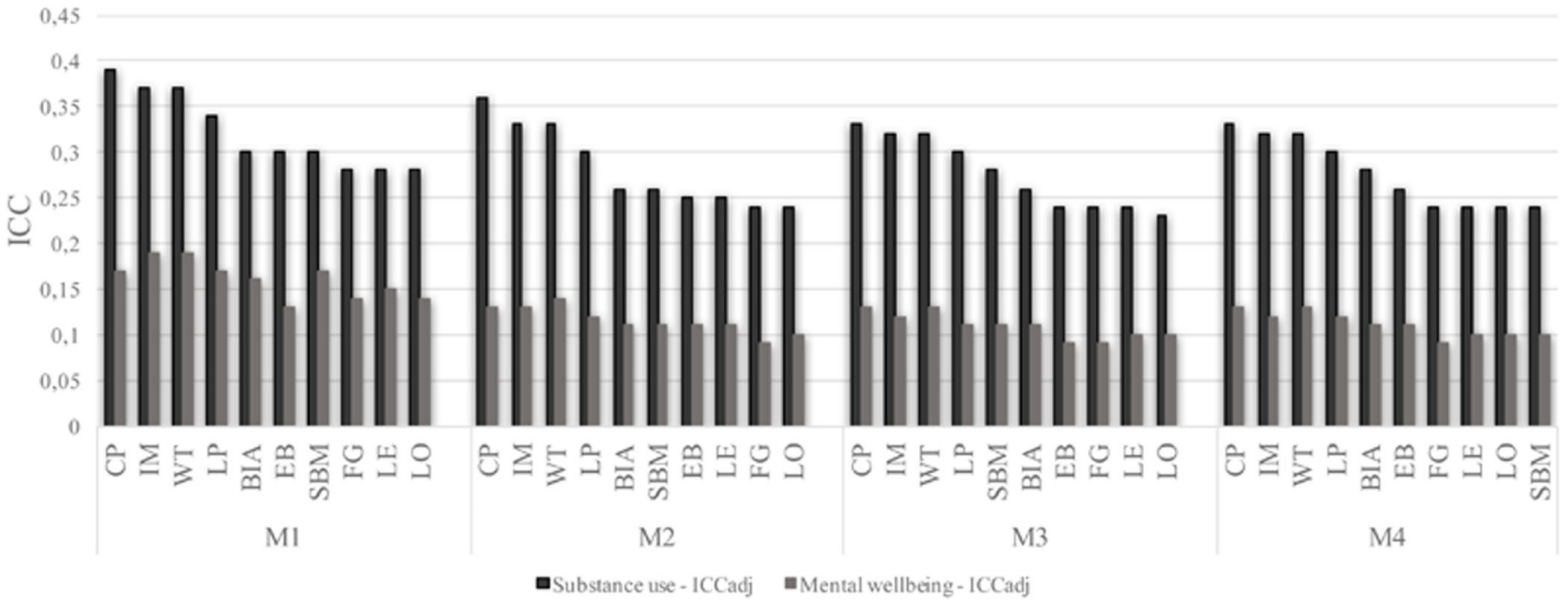
Intraclass correlation coefficients (ICC, y-axis) for models M1, M2, M3, and M4 for two health outcomes and 10 GDMs, ordered by the highest ICC in Substance Use (-).

We reran multilevel models, updating the group properties (level 2 covariates) and level 2 random effect according to the specific groups returned from each GDM. Fig 5 shows ICCs and for models M1, M2, M3, and M4, for each GDM, ordered by highest ICCs in Substance Use.

Irrespective of model and group detection method (GDM), there was greater clustering for substance use than for mental wellbeing (Fig 5). According to model 1, between 28% and 39% (depending on a GDM) of variation in Substance Use between individuals was associated with community membership, while for Mental Wellbeing it is between 13% and 19%. For model 1, there were no GDMs where the between-group variation was very low or negligible (ICC <0.1) for Substance Use or Mental Wellbeing. CP recovered highest ICC for Substance Use, while WT and IM recovered highest ICC for Mental Wellbeing.

### Sensitivity of effects of group properties on health to group detection method

Tables 6 and 7 show estimates and *p*-values for community properties in Model 3 for ten GDMs (ordered alphabetically), for Substance Use and Mental Wellbeing, respectively. *p*-values corrected for multi-testing are reported in Supplementary Materials (section 6.1).

Additionally, estimates and *p*-values for covariates-unadjusted model and community property’s quadratic term are presented is Supplementary Materials in tables S19 and S20.

There is evidence of non-linear relationships with Substance Use for hierarchy (four GDMs, negative quadratic term) community size (for IM, negative term). Regarding Mental Wellbeing, there are substantially more non-linear relationships detected. Four GDMs had curvilinear relationship with centralization (three of which had negative and one positive quadratic term), and three GDMs for transitivity (two positive and one negative quadratic term). Two GDMs found negative non-linear relationships between ratio of ties outside community and Mental

Wellbeing, and one for size (positive) and hierarchy (negative). Of all GDMs, only with CP, non-linear effects were not detected.

GDMs differ regarding significant linear effects that are found for community properties. However, for the two health outcomes, the direction of community effects, when significant (*p*<0.1), is the same for all GDMs, suggesting that different GDMs tend to converge to consensus when the effect is found. The patterns of significant effects differ substantially between covariates-unadjusted model and Model 3. In the previous, gender community composition (male versus female, positive estimate) was significant for all GDMs and both outcomes. The same applies to gender community composition (mixed versus female, positive estimate) for Mental Wellbeing, but for Substance Use, only one GDM showed significant effect. Ratio of ties outside community had significant and positive estimate for all but two GDMs, but only when Mental Wellbeing was the outcome. Transitivity had significant and negative effect to Mental Wellbeing for two GDMs, and for one GDM it had positive and significant effect on Substance Use. Hierarchy had negative and significant effect on Substance Use only (two GDMs). Positive effects of community size on Substance Use were found for two GDMs, but none for Mental Wellbeing. Centralization had one positive effect for each outcome (one GDM).

In Model 3, for most community properties less than half of GDMs found evidence of an association with a health outcome, except for transitivity which was found to be significantly associated with Substance Use with half (five) of the GDMs. For community size, for both outcomes there is also some support across GDMs (three of ten GDMs), while for gender composition there are four of ten GDMs showing an association with Substance Use. Most community properties were found to be significantly associated with each outcome for at least one GDM. WT uncovered the highest number of significant community properties for both outcomes (three), while IM did not detect any significant properties for both outcomes. When multi-testing is considered, we can see that in case of WT, transitivity for both outcomes, and community size for Mental Wellbeing have p-values (=<0.10) that suggest that effects are detected even after correcting for false discovery rate. Finally, we see that there is some evidence that hierarchy is associated with Substance Use (four of ten GDMs), and centralization for both Substance Use and Mental Wellbeing (three of ten), but this is not captured by the WT method. Only for ratio of ties outside community we found no evidence of association with Substance Use, based on any GDM used in this study.

## Discussion

This study makes two key contributions to school health improvement research. First, moving beyond peer similarity on individual health outcomes, this is the first demonstration that structural and compositional characteristics of peer groups are associated with individual substance use and mental wellbeing. Second, we have outlined the extent to which methodological decisions around group detection methods affect the ability to statistically assess meso level, peer group processes affecting health. These findings have implications for the design of network interventions (Valente, 2012) and research into school-health and adolescent social development.

### Clustering of health within peer groups

Our analysis found that clustering is relatively high for Substance Use, and moderate for Mental Wellbeing. Substance use shows more clustering within school peer groups, but for both, clustering is found even after controlling for individual and school covariates, supporting H1 hypothesis. Higher clustering of Substance Use than for Mental Wellbeing could be due to a generally higher social “visibility” or observability of the former compared to the latter. That is, descriptive and injunctive norms may be more easily deduced by observations of others for substance use than for mental wellbeing. The difference in visibility also comes from the different roles these behaviours play in social activities, social functions they serve, and their perceived benefits for young people. Internalizing problems – such as low self-esteem or anxiety – are by their nature less visible or readily observable and may be associated with more stigma than substance use or other health-related behaviours which often occur in groups and social settings. Furthermore, adolescents may hide or disguise their distress (e.g., anxiety, depression) in school context (Flett et al., 2018). Due to this difference in visibility, the mechanisms of social influence or friend selection, and the resulting social clustering may differ. It is also possible that measurement error variance for Mental Wellbeing is greater than for Substance Use, and this would also reduce group level variation.

Previous research of different health outcomes and on bigger samples but using a similar analytical strategy found that variation between peer groups explains around 2.5% of variance in students’ body mass index (Evans et al., 2016) and around 3.7% of variance in age of sexual initiation (Barker et al., 2019). We found a notably higher group level variation for Substance Use and Mental Wellbeing. However, the results are not directly comparable for several reasons: the final models in these studies included different individual covariates with neighbourhoods and schools as random effects. We did not find school level effects, possibly due to our more homogeneous sample of mostly urban, mixed-gender schools. Lower group variation may also be due to the outcomes themselves. Finally, in both studies the Louvain (LO) method was used as the main GDM, and Clique Percolation (CP) for the sensitivity analysis. The latter showed substantially higher variation between communities (11.2% in Evans et al. (2016), but not reported in Barker et al. (2019)). We found that LO was among GDMs with the lowest ICCs, suggesting the possibility that the between group variation may have been higher if the study used alternative GDMs, such as Walktrap.

### The importance of the meso level: Associations of community (peer group) properties with two health outcomes

Previous research has looked only at group level clustering and not at information about other group properties, such as its size, transitivity or hierarchy. This gap in understanding of how features of peer group affect individual health has held back understanding of the health implications of social groups; and has hindered the development of theory around meso level effects of social groups on adolescent wellbeing. Our exploratory analysis had provided insights to support future work around meso level theories of health and wellbeing. We found that by using WT some community properties were significantly associated with individual health outcomes, providing support of H2 hypothesis.

When controlling for individual covariates and other community properties, found effects were substantially different, as we expected that some individual attributes will be associated with both community properties and outcomes (e.g., gender), and that community properties are interrelated. We found that the same community property can show associations in opposite directions for different aspects of health (e.g., transitivity). This suggests that a community property cannot be considered universally good or bad for any individual health outcome. Nonetheless, curvilinear relationships between some community properties and outcomes are detected, more so for Mental Wellbeing as the outcome. Therefore, only a more nuanced understanding of the social processes within groups can provide insights into causal factors, and thus appropriate targeting of intervention strategies.

### Associations of community properties and individual substance use and mental wellbeing – possible explanations

Given that we did not start with specific hypotheses or causal models about the direction of effects and their mechanism, but rather only tentative and broad expectations, we outline some initial considerations on how group characteristics may affect health to support formal theory building, hypothesis testing and replication studies using other datasets. As there were a large set of individual, group and school factors to consider, we discuss only a subset of the model results that are related with groups’ properties for which there was evidence of association with individual health across multiple GDMs and in covariate-adjusted models (Model 3) models. Importantly, due to the interrelated nature of community properties, found non-linear effects, and other considerations mentioned in the section Analytical strategy, any interpretations of Model 3 should be considered as highly tentative.

#### Community size

Bigger communities, when controlling for other properties, are related to less Substance Use and worse Mental Wellbeing, although the unadjusted estimate suggests no association (see Supplementary Materials, table S19). Smaller groups may provide greater discretion due to higher closeness (Morgan & Grube, 1991), that is, facilitate greater secrecy around illicit behaviour. For drug use to be viewed as acceptable and normative behaviour, it may require a majority of peer group members to participate in that behaviour or comply with it (Allison et al., 1999, Eisenberg et al., 2014). That majority is more difficult to reach in bigger groups. Alternatively, those using more substances could be less likely to join big groups, because of the difference in normative behaviours or a preference for smaller, more secretive peers. However, there are potentially some less positive aspects of being in a bigger group that may have a negative effect on individual wellbeing. Larger communities also carry more social uncertainty in that they are more likely to include people that one does not know or like, and there is greater opportunity to become peripheral to the group. Also, small groups can provide more opportunities for development of close friendships that entail intimacy, self-disclosure, and acceptance than bigger groups (e.g., McElhaney et al., 2008).

#### Transitivity

Being in a more transitive peer group is associated with lower substance use. This could be explained with the notion of social capital that is associated with more transitive groups and it provides context for trust and disclosure. Alternatively, students that do not use substances may be more prone to form transitive groups. But we found that transitive groups are associated with worse mental wellbeing as well, although the latter was not found with covariates-unadjusted model. Since model with only transitivity and random effects of groups for all GDMs showed negative estimate for association of this property and Mental Wellbeing (but significant only for blockmodeling based GDMs), we will shortly discuss it. In transitive groups, there may be a higher control, closeness (structurally strong ties), and awareness of members’ behaviours which could deter substance use. It is possible that those aspects or some other aspect of transitive groups can also lead to its negative associations with Mental Wellbeing, in part due to possibly stronger social influence and diffusion potential – if some members had poor mental health, such setting could amplify contagion. Given that for some GDMs, non-linear effect is detected for transitivity-mental wellbeing relationship (see table S20 in SM), it is possible that the relationship is more complex. Overall, this group property showed among the most consistent findings across the various GDMs (in Models 3), which suggests that further theorising and understanding of how the transitivity of groups emerges, changes, and affects health should be a priority for future work.

#### Centralization

Being in a more centralized group is associated with worse Mental Wellbeing in Model 3. In such groups, some members have more ties than others, implying that they have a higher social status or are simply more active members of a community, while others are less popular or visible. This may suggest that groups with a greater status differences place group members under greater stress, either to maintain status or because they see themselves as less well liked by others in their social reference group. Given that there was evidence of non-linear, inverted U-shaped relationship (for WT and some other GDMs, when not controlling for covariates), suggesting diminishing returns of centralization in relation to one’s mental wellbeing. To our knowledge, these structural effects have not been previously studied and further consideration of these social processes is justified.

The effects of gender composition were not completely “washed-out” for all GDMs when controlling for gender and other community properties which could be interpreted to suggest that gender composition of groups may involve specific dynamics associated with health outcomes. Ratio of ties outside community had significant and positive effect on Mental Wellbeing for all but two GDMs in covariates-unadjusted models. This suggests that more open communities are better for individual wellbeing, possibly because the being a non-isolated group provides access to diversity of norms and different types of relationships. Alternatively, and not mutually exclusive, adolescents with better mental wellbeing may have tendency to join or maintain more open groups. We found some confirmation of diminishing return of this property to mental wellbeing for some GDMs, but the direction of quadratic term was not consistent.

Some community properties may play a role in shaping student health in schools. This information may help to inform the design of a network segmentation intervention. To date, most studies have used a node selection or peer influence approach rather than focussing on identifying segments relevant for intervention (for a recent review see Hunter et al., 2019). Our findings suggest that, beyond simply identifying groups to receive the active ingredients of an intervention, better understanding of the variations in structures of the target groups could be used to enhance how the intervention may operate. For example, a segmentation intervention targeting a highly transitive group may take an intensive approach that ensures the higher collective social control within the group does not counteract individual behaviour change, while low transitivity groups may require a different deployment of resources or fewer group members to elicit change. These possibilities warrant much closer theoretical and empirical attention, particularly as the identification of peer groups is fast and low cost (e.g., select the school peers who have lunch together) in comparison to identifying peer leaders from a sociometric survey of the whole school.

### Sensitivity of findings to group detection method

The finding of peer group variation in health are reasonably robust to the choice of GDM, but choice of GDM can substantially affect findings around the association of group characteristics and health – supporting our H3 hypothesis. Using LO would lead to a between-group variation estimate that was 69% of the estimate returned using CP. If we used IM, we would find no evidence of association between community structure and health (in covariates-adjusted models) but would find several significant associations using WT or LE.

Considering both health outcomes and Model 1, the GDMs showing the highest between group variation in health are CP, IM, and WT, while the lowest variation is found for FG, EB, LE and LO. This may be due to FG, LE, and LO detecting communities that are larger. In difference with CP, IM, and WT, that may be more sensitive to network’s local structures and provide more fine-grained community as they are clique-based, based on random walks, and based on optimization information flow, respectively. The finding for EB has a less obvious explanation and it is surprising because it is one of the most frequently used algorithms, but the algorithm is divisive and does not optimise modularity but betweenness centrality of edges within a network. Although blockmodeling-based methods, BIA and SBM, are not based on notion of cohesive or distinct communities, they also uncovered groups that vary in health outcomes, and some of their community properties had effects on individual health. There is an important role for theory around meso level social processes to make sense of the qualitative differences in information returned by various GDMs.

Higher between group variance does not mean that detected groups are more valid or closer to a ground truth. Homophily operates on many dimensions besides health (Block & Grund, 2014) and peer groups may form and change for many reasons. It is possible to model meaningful social structures related to health even without having a ground truth data of peer groups within the study schools. While we do not make any claim around which GDM is most suitable for modelling, the plausible values for between peer group variation fall between 28 and 39% for Substance Use and 13 and 19% for Mental Wellbeing, both ranges indicating that clustering based on those health outcomes exists.

From an analytical perspective, using WT produced fewer very small communities (having three or less members, see Table S15 in Supplementary Materials), this facilitates the study of group structure which requires a larger number of group members to calculate, but at the expense of underestimating the genuine occurrence of smaller groups.

### Some guidelines for choosing a GDM

Bothorel et al. (2021) recently proposed a methodology for choosing a GDM consisting of several steps which resemble the procedure we used in this study. Our approach diverges as our goal was not to choose the most optimal GDM, but rather to study meso level effects and check sensitivity of findings to GDM used. We both used principal component analysis on several node attributes to measure homophily within communities. While Bothorel et al.’s procedure suggested this measure as an indicator for choosing a method, we caution against such an approach. Smith et al. (2020) advise using LO (e.g., Evans et al., 2016) or Spinglass (the latter not included in our study) when GDM is a part of a wider analytical strategy. Following this advice would have given a comparable between group variation to the other GDMs, and somewhat fewer associations between community properties and health outcomes (see Tables 6 and 7). Our aim was not to decide which GDM is “optimal”, as any data-driven approach may overlook the important aspects of group structure in favour of a chance partitioning of the data. However, by gauging the sensitivity of our findings to the GDM, we make some observations regarding relevant considerations when choosing a GDM in similar research and intervention contexts.

It is often noted (e.g., Smith et al., 2020) that features of networks under study may be helpful for the selection of the GDM. For instance, some algorithms are not implemented for directed networks, so it would make sense to exclude them in situations when directed network data is available as it would lead to loss of potentially useful information. However, as we found from the GDMs used in this study (CP, FG, LE, LO, and LP), the information about directionality did not greatly affect the between group variation and community properties associated with outcomes. For our outcomes of interest, it may be that the relevant social influence processes happen within communities, and they do not require reciprocated or even direct ties. Our findings suggest that directionality information should not rule out the use of a GDM for a schools-health project. For example, some peer groups may benefit from an intervention with smoking prevention resources, while other peer groups in the same school may gain less benefit from prevention but greater benefit from cessation resources.

An additional consideration relates to the size of communities. Studying social processes related to group characteristics such as transitivity and gender composition is less feasible and meaningful with very small or very big groups. According to the literature (Cotterell, 1997), big and small groups in adolescence have different social functions and dynamics, and they may therefore present a qualitatively different social environment. EB produced the highest number of big communities (*N*>30), while BIA resulted in the biggest communities on average. CP, EB and SBM returned a high number of small groups, producing missing data on the group level variables. Depending on a GDM, 1% (FG) to 16.5% (SBM) observations were dropped from Model 1 to Model 3 for this reason (see table 18 in SM). One approach would be to choose a GDM that produces communities within a predefined range for group size. A limitation of that approach is that it precludes the study of what may be genuine isolates, dyads or very small or very big groups which may be relevant for the specific topic of the project. Social processes may operate very differently between small, moderate and large peer groups, which further reinforces the importance of closer consideration of theory about meso level properties and group formation and influence.

Different goals of using GDMs make some considerations more important than others and may lead to different choices of an “optimal” GDM. The caveat is that many of the important considerations can be decided upon post hoc after applying a range of methods and studying the characteristics of the groups. With this approach, it is still possible to provide a priori criterion around which GDM to use for analysis e.g., “We will select the GDM where less than 10% of the nodes are placed in groups with five or fewer members”.

An alternative approach – which can be combined with the exclusions mentioned above - is to consider an ensemble modelling approach. Our results found that between group variance was substantial regardless of GDM used. Fitting a range of models can demonstrate the range of plausible values for between peer group variance. Techniques developed for meta-analysis of results from multiple studies can be used to identify whether consistent associations appear after accounting for multiple comparisons (see figure S20 in Supplementary Materials), and the extent to which GDMs return widely different insights around meso level processes (e.g., were there large or small between-GDM differences in the number of very small groups).

### Practical implications

Since adolescence is the period of onset for many novel health behaviours and wellbeing outcomes (Kaltiala-Heino et al., 2011), it is also a development period when preventive efforts can be particularly impactful. Finding peer clustering for both substance use and mental wellbeing suggests that school level interventions may operate differently across segments of the school population. GDMs could identify groups of a specific interest which may benefit from more tailor-made interventions. Based on our findings, community properties of peer groups in schools are associated with individual health outcomes. Our results suggest there may be opposite answers depending on the outcome of interest. All of these potential trends require further scrutiny and hypotheses testing in observational or intervention research. As such, health improvement interventions must carefully consider the contextual features of the peer groups in which any health improvement intervention takes place.

### Limitations of the study and future research

There are several limitations of the study, which also suggest avenues for future research. Cross-sectional data did not allow causal claims about community properties and individual health outcomes. Communities, in addition to not being static, can also overlap. Since all GDMs except CP could detect only exclusive groups, we used an analytical approach (hierarchical MLM) that was best suited for nine GDMs, but not for CP. Future research using CP could use multiple membership multilevel models to appropriately test CP’s community structure. We offered some plausible explanations for found community property effects, but systematic reviews of adolescent health literature, including findings coming from research using other (e.g., qualitative) methods is needed to set the stage for building more nuanced meso level theories.

We used data from an earlier study, collected when social network platforms and on-line communication were not nearly as ubiquitous as today. Social media has brought significant change to the ways in which peer groups interact, as well as to the ‘visibility’ of health behaviours – and these may have an impact on which community properties are most important. In addition, research suggests secular trends in substance use and mental health have changed in recent decades, showing decrease in substance use and increase in poor mental health among adolescents (Kraus et al., 2018; Collishaw, 2015) and these differences will likely affect clustering in these outcomes within peer groups. Repetition of this analysis in contemporary peer groups is an important next step. Our analysis is restricted to friends at the same school and year. Although the majority of peer ties tend to be from the same school and year (Ennett & Bauman, 1996), adolescents may have peers of different ages and peers outside school who influence their substance use and mental wellbeing. Controlling for other contextual factors that may facilitate social clustering such as geographical proximity (e.g., neighbourhoods), friendships choice constraints, exposure to similar media influences, shared activities, etc., can further clarify the role of peer groups in individual health outcomes. From the micro level perspective, the role of other potentially relevant individual factors may be investigated (existence of health conditions, health interests and knowledge) and group composition based on other attributes besides gender that employs different ways to describe the distribution of these attributes within peer group can be explored. We used single imputation for attributes of study participants, but future research dealing with extensive missing data should consider multiple imputations.

While we did not find evidence for effects of macro level properties (i.e., school effects) in our study, this does not mean network macro level has no influence on individual health. Future studies should try to identify and consider school contextual effects as the important properties at macro level with more heterogeneous samples of schools and investigate their potential effects and interactions with meso (and micro) level effects. For example. substance use in a school with high prevalence may be qualitatively different to substance use in a low prevalence school, and this may also be expressed in meso level differences e.g., the perceived differential between the peer group norm and the wider school norms (“small fish in a big pond/big fish in a small pond” described by Lazega et al., 2008). Furthermore, future research could consider other approaches to identifying meso level, e.g., relying on social identities and roles (e.g., by using latent class modelling or bipartite networks) rather than connection patterns. Going beyond simple graphs and using hypergraphs (Estrada & Rodriguez-Velazquez, 2005) presents another way to represent and study network’s meso level. We investigated six community properties, but there are other potential properties that could be relevant for individual health. For instance, centrality of a group relative to other groups in the network as well as the centrality in the peer group as individual variable could be included as attribute data when using advanced network methods e.g., Bayesian hierarchical exponential random graph models (Agneessens et al., 2022), latent space network models (Mathews & Volfovsky, 2021), or multilevel exponential random graph models or auto-logistic actor attribute models (Lazega & Wang, 2023). The meso level theorising that we advocate for could apply equally well to other methods used for collecting data about peer groups, and future research would benefit by incorporating different types of data. Cultural and gender differences in clustering and relevance of community properties may exist, so research examining gender effects and different cultural settings is called for. Finally, other individual outcomes, besides health-related, such as aggressiveness, delinquency and academic achievement can also be analysed on meso–level in future research.

## Supplementary Material

Supplementary Materials

## Figures and Tables

**Table 1:** Family and health-related measures used in PaLS study.

*Measure, scale*	*N of items, example*	*Cronbach* *alpha*	*Scoring*
Family related variables			
Family affluence, Family Affluence Scale (Boyce et al., 2006)	4 items, “Does your family own a car, van or truck?”	0.49	Range from 0 to 7, higher scores signifying higher family affluence. Due to high negative skewness (-0.56) categorised in one of the following groups: “low”, “medium”, and “high”, representing values 0-3, 4-5, 6-7, respectively
Parental care and control, Parental Bonding Instrument brief form (Parker, 1979; Klimidis et al., 1992)	8 items in total; 4 about parental care (e.g., “My parents are loving”); 4 about parental control (e.g., “My parents, try to control everything I do”)	Parental care: 0.72; Parental control: 0.59	For both subscales, range from 0 to 8, a higher score – higher parental care/control
Health-related variables			
Smoking	One question about the frequency of smoking	/	Range from “I have never smoked at all (not even a puff)” (1) to “I smoke regularly (1 or more cigarettes a week)” (5)
Drinking	One question about the frequency of drinking	/	Range from drinking “every day” (1) to never having an alcoholic drink (8)
Using drugs	Asked how often they use the following drugs (not including drugs that the doctor or chemist has prescribed to them): cannabis, Valium, amphetamine, LSD, ecstasy, solvents, cocaine, heroin, and magic mushrooms. The questionnaire included colloquial (street) names for these drugs as well. For each drug, five possible answers ranged from “Every day” to “Never”.	/	Due to overall low prevalence of drug use, the total score is calculated such that if a participant had reported using any drug every day or weekly, less often, or never, the assigned values were 2, 1, and 0, respectively (higher values – more frequent use).
Drug effects (recent drug-related experiences and dependence)	3 items referring to the last month, “I forgot things I did due to drugs.”, possible answers from “Every day” (1) to “Never” (5)	0.79	Range from 1 to 15, lower scores – higher dependence
Self-esteem, scale based on Rosenberg (Rosenberg, 1965)	10 items, “I am pretty sure of myself”, possible answers from “Strongly agree” (4) to “Strongly disagree” (1)	0.86	Range from 0 to 30, higher score – higher self-esteem
General mental health, General Health Questionnaire, GHQ-12, based on (Goldberg, 1978)	12 questions about health in general in the past few weeks, “Have you recently been able to concentrate on whatever you’re doing?”. Potential answers from “Better than usual” (1) to “Much less than usual” (4)	0.85	Range from 0 to 36, higher score – worse mental health
Worries	10 items on extent of worry about common worries for teenagers (e.g., “Doing well at school”, “My looks”), possible answers “A lot” (1), “A bit” (2), and “Not at all” (3)	0.78	Range from 0 to 30, higher score – having less worries

/ - not applicable

**Table 2:** Demographic characteristics of the PaLS participants (N = 3194).

*Variable*		*Categories*		N total*
**Gender**	*Female*	*Male*		
*N*	1622	1572		3194
*%*	50.8	49.2		
**Ethnicity**	*White*	*Other*		
*N*	2808	246		3054
*%*	87.9	7.7		
**Age**	*15*	*16 and 17*		3190
*N*	1615	1575		
*%*	50.6	49.3		
**Family** **affluence**	*Low*	*Medium*	*High*	
*N*	496	1280	1309	3085
*%*	15.5	40.1	41.0	3.4

*N total* –total number of all cases with no missing data; *N* – number of

cases in each category; % - percentage of cases in each category

**Table 3:** Dependent variable: Substance Use (-) -– results for Walktrap community detection algorithm.

Parameters/ Models	Estimate [95% confidence intervals]				
	M1	M1.1	M2	M3	M4
(Intercept)	-0.07 [-0.14, 0]	-0.07 [-0.14, 0]	0.26 [0.11, 0.41]	-0.16 [-0.54, 0.22]	-0.27 [-1.84, 1.3]
*Level 1 covariates*					
Gender (male)			**0.24 [0.16, 0.33]**	**0.21 [0.1, 0.31]**	**0.21 [0.1, 0.31]**
Age			**-0.08 [-0.14, -0.03]**	**-0.08 [-0.14, -0.03]**	**-0.08 [-0.14, -0.03]**
Ethnicity (white)			**-0.48 [-0.6, -0.36]**	**-0.47 [-0.59, -0.35]**	**-0.47 [-0.59, -0.35]**
Family affluence (medium)			0.08 [0, 0.17]	0.07 [-0.01, 0.16]	0.07 [-0.01, 0.16]
Family affluence (high)			0.04 [-0.05, 0.13]	0.03 [-0.06, 0.12]	0.03 [-0.06, 0.12]
Parental control			**-0.11 [-0.14, -0.08]**	**-0.11 [-0.14, -0.08]**	**-0.11 [-0.14, -0.08]**
Parental care			**0.23 [0.2, 0.26]**	**0.23 [0.2, 0.26]**	**0.23 [0.2, 0.26]**
*Level 2 covariates*					
Community size				**0.13 [0.01, 0.25]**	**0.14 [0.01, 0.26]**
Community gender comp.(male)				0.16 [-0.02, 0.35]	0.15 [-0.04, 0.34]
Community gender comp.(mixed)				-0.06 [-0.24, 0.12]	-0.07 [-0.25, 0.11]
Ratio of ties outside community				0.09 [-0.48, 0.65]	0.19 [-0.46, 0.85]
Transitivity				**0.63 [0.25, 1.01]**	**0.67 [0.28, 1.07]**
Centralization				0.23 [-0.35, 0.8]	0.23 [-0.34, 0.8]
Hierarchy				-0.06 [-0.39, 0.28]	-0.06 [-0.41, 0.28]
*Level 3 covariates*					
School/network size					-0.03 [-0.12, 0.07]
Modularity (school)					0.64 [-1.23, 2.52]
Prop. F in school					-0.7 [-2.04, 0.64]
Num. obs.	3148	3148	3148	3079	3079
AIC	8228.78	8230.78	7833.15	7633.09	7639.32
BIC	8246.95	8255.00	7893.69	7735.64	7759.97
Log Likelihood	-4111.39	-4111.39	-3906.57	-3799.55	-3799.66
Var: Residual	0.66	0.66	0.58	0.58	0.58
N groups: Community	387	387	387	339	339
Var: Community (Intercept)	0.38	0.38	0.29	0.27	0.27
N groups: School		22			
Var: School (Intercept)		0.00			
ICCadj./ICCcond.	0.37/0.37	0.00/NA	0.33/0.29	0.29/0.27	0.29/0.27
*R*^2^mar./ *R*^2^cond.	0/0.37	0/0.37	0.12/0.41	0.14/0.41	0.14/0.41

Abbreviations for Tables 4 and 5: Community gender comp. – community gender composition; Prop. F in school – proportion of females in the school; Num. obs. – Number of observations; AIC – Akaike information criterion; BIC – Bayesian information criterion; Var – variance; N groups – number of groups; ICCadj. – adjusted intraclass correlation coefficient; ICCcond. – conditional intraclass correlation coefficient; *R*^2^mar. – marginal *R*^2^; *R*^2^cond. – conditional *R*^2^; Age is dichotomized: 15 yrs = 0; 16 and 17 yrs = 1; Reference categories for factors: Gender: male; Ethnicity – white; Family affluence – low; Community gender comp. – female.

**Table 4:** Dependent variable: Mental Wellbeing (+) – results for Walktrap community detection algorithm.

Parameters/ Models	Estimate [95% confidence intervals]				
	M1	M1.1	M2	M3	M4
(Intercept)	0 [-0.06, 0.05] 0 [-0.06, 0.05] -0.38 [-0.53, -0.23]			0.15 [-0.16, 0.46]	0.28 [-0.92, 1.48]
*1 covariates*					
Gender (male)			**0.56 [0.48, 0.64]**	**0.56 [0.45, 0.68]**	**0.56 [0.45, 0.68]**
Age			0.02 [-0.05, 0.08]	0.01 [-0.05, 0.07]	0.01 [-0.05, 0.07]
Ethnicity (white)			**0.14 [0.02, 0.26]**	0.12 [-0.01, 0.24]	0.11 [-0.01, 0.24]
Family affluence (medium)			-0.04 [-0.13, 0.05]	-0.04 [-0.14, 0.05]	-0.04 [-0.14, 0.05]
Family affluence (high)			-0.02 [-0.12, 0.07]	-0.02 [-0.12, 0.08]	-0.02 [-0.12, 0.08]
Parental control			**-0.17 [-0.21, -0.14]**	**-0.18 [-0.21, -0.15]**	**-0.18 [-0.21, -0.15]**
Parental care			**0.09 [0.06, 0.13]**	**0.09 [0.05, 0.12]**	**0.09 [0.05, 0.12]**
*Level 2 covariates*					
Community size				**-0.13 [-0.22, -0.04]**	**-0.14 [-0.23, -0.05]**
Community gender comp.(male)				0.01 [-0.15, 0.18]	0.02 [-0.14, 0.18]
Community gender comp.(mixed)				-0.02 [-0.15, 0.12]	-0.01 [-0.15, 0.13]
Ratio of ties outside community				-0.14 [-0.58, 0.3]	-0.22 [-0.74, 0.29]
Transitivity				**-0.6 [-0.9, -0.29]**	**-0.63 [-0.94, -0.31]**
Centralization				**-0.58 [-1.05, -0.12]**	**-0.58 [-1.04, -0.12]**
Hierarchy				0.03 [-0.25, 0.31]	0.04 [-0.24, 0.33]
*Level 3 covariates*					
School/network size					0.01 [-0.06, 0.09]
Modularity (school)					-0.57 [-1.99, 0.86]
Prop. F in school					0.58 [-0.43, 1.59]
Num. obs.	3148	3148	3148	3079	3079
AIC	8556.69	8558.69	8214.19	8029.59	8037.24
BIC	8574.85	8582.90	8274.73	8132.14	8157.89
Log Likelihood	-4275.34	-4275.34	-4097.09	-3997.80	-3998.62
Var: Residual	0.79	0.79	0.72	0.71	0.71
N groups: Community	387	387	387	339	339
Var: Community (Intercept)	0.19	0.19	0.11	0.10	0.10
N groups: School		22			
Var: School (Intercept)		0.00			
ICCadj./ICCcond.	0.19/0.19	0.00/NA	0.14/0.12	0.13/0.11	0.13/0.11
*R*^2^mar./ *R*^2^cond.	0/0.19	0/0.19	0.15/0.26	0.15/0.26	0.15/0.26

**Table 5:** Estimates of effects of community properties on Substance Use (-) and Mental Wellbeing (+) for models with one community property and random effects of communities (Walktrap).

*Community property*	*Substance Use (-)*			*Mental wellbeing (+)*		
	*Estimate*	*SE*	*p*	*Estimate*	*SE*	*p*
Community size	0.05	0.046	0.269	0.01	0.035	0.843
Community gender comp.(male)	0.35	0.082	**<0.001**	0.6	0.06	**<0.001**
Community gender comp.(mixed)	0.05	0.091	0.618	0.2	0.064	**0.002**
Ratio of ties outside community	-0.11	0.258	0.679	0.59	0.216	**0.007**
Transitivity	0.31	0.157	**0.047**	-0.31	0.132	0.019
Centralization	-0.25	0.262	0.336	0.01	0.216	0.954
Hierarchy	-0.02	0.174	0.925	-0.1	0.147	0.479

GDM – group detection method; SE: Standard error

Bold font – *p* value =<0.10

**Table 6:** Community property effects for Substance Use (-) (Model 3) for 10 GDMs.

GDM		Size	Gender. comp. male	Gender. comp. mixed	ROTC	Transitivity	Centralization	Hierarchy
BIA	Est.	0.04	0.026	-0.077	-0.355	0.346	0.292	-0.613
	*p*	0.551	0.802	0.397	0.187	0.196	0.508	**0.055**
CP	Est.	0.134	0.18	-0.033	-0.345	0.399	0.071	-0.286
	*p*	0.161	**0.052**	0.739	0.109	**0.093**	0.804	**0.024**
EB	Est.	0.045	0.311	-0.011	-0.181	0.214	-0.057	-0.105
	*p*	0.592	**0.001**	0.913	0.351	0.276	0.838	0.551
FG	Est.	0.07	0.081	-0.031	-0.407	0.364	0.797	-0.769
	*p*	0.296	0.446	0.736	0.195	0.148	**0.04**	**0.014**
IM	Est.	0.022	0.067	-0.016	-0.138	0.126	-0.18	-0.177
	*p*	0.602	0.469	0.852	0.504	0.412	0.404	0.221
LE	Est.	0.098	0.073	-0.011	-0.013	0.593	1.512	-0.723
	*p*	**0.076**	0.473	0.905	0.967	**0.027**	**0.001**	**0.03**
LO	Est.	0.028	0.023	0.03	-0.16	0.6	0.746	-0.516
	*p*	0.607	0.819	0.734	0.631	**0.029**	**0.093**	0.111
LP	Est.	0.133	0.147	0.008	-0.257	0.458	0.231	-0.007
	*p*	**0.018**	0.118	0.928	0.248	**0.013**	0.432	0.964
SBM	Est.	0.017	0.243	-0.07	0.031	0.225	0.088	-0.234
	*p*	0.807	**0.009**	0.449	0.85	0.123	0.645	0.134
WT	Est.	0.128	0.16	-0.063	0.088	0.63	0.227	-0.056
	*p*	**0.04**	**0.092**	0.493	0.763	**0.001**	0.444	0.748

GDM (group detection methods) abbreviations: blockmodeling with indirect approach – BIA, Clique percolation

– CP, Edge-betweenness – EB, Fast greedy – FG, Infomap – IM, Leiden – LE, Louvain – LO, Label propagation

– LP, stochastic blockmodeling – SBM, Walktrap – WT.

Other abbreviations: Est. – estimate; *p* – *p*-value; Gender.comp. – gender composition of communityReference group for Gender composition: female Gray cells: *p*-values Bold font: *p*-values =< than 0.10 Underlined and bold – *p*-values =<0.10 after correction for multi-testing (false discovery rate)

**Table 7:** Community property effects for Mental Wellbeing (+) (Model 3) for 10 GDMs.

GDM		Size	Gender. comp. male	Gender. comp. mixed	ROTC	Transitivity	Centralization	Hierarchy
BIA	Est.	-0.085	-0.058	-0.047	0.372	-0.328	-0.435	0.237
	*p*	0.087	0.507	0.5	**0.078**	0.12	0.219	0.383
CP	Est.	-0.038	-0.007	-0.046	0.235	-0.31	-0.124	0.222
	*p*	0.564	0.935	0.55	0.174	**0.098**	0.587	**0.039**
EB	Est.	-0.038	-0.155	-0.178	0.055	-0.33	-0.113	-0.084
	*p*	0.515	**0.058**	**0.02**	0.725	**0.034**	0.618	0.576
FG	Est.	-0.014	-0.067	-0.06	0.137	-0.16	-0.371	0.267
	*p*	0.765	0.437	0.381	0.576	0.427	0.236	0.313
IM	Est.	-0.016	0.018	-0.087	0.107	-0.05	-0.019	0.106
	*p*	0.631	0.836	0.195	0.517	0.689	0.915	0.382
LE	Est.	-0.06	-0.081	-0.113	0.069	-0.286	-0.708	0.016
	*p*	0.158	0.342	0.105	0.789	0.189	**0.048**	0.955
LO	Est.	-0.069	-0.058	-0.117	0.18	-0.134	-0.702	-0.201
	*p*	**0.09**	0.494	0.086	0.485	0.541	**0.05**	0.465
LP	Est.	-0.08	-0.063	-0.061	0.182	-0.212	-0.22	0.004
	*p*	**0.052**	0.453	0.375	0.299	0.156	0.348	0.974
SBM	Est.	0.023	-0.032	-0.086	0.063	-0.094	0.07	0.036
	*p*	0.655	0.715	0.251	0.632	0.42	0.656	0.786
WT	Est.	-0.133	0.014	-0.018	-0.141	-0.596	-0.585	0.033
	*p*	**0.004**	0.866	0.798	0.535	**<0.001**	**0.014**	0.817

GDM (group detection methods) abbreviations: blockmodeling with indirect approach – BIA, Clique percolation

– CP, Edge-betweenness – EB, Fast greedy – FG, Infomap – IM, Leiden – LE, Louvain – LO, Label propagation

– LP, stochastic blockmodeling – SBM, Walktrap – WT.

Other abbreviations: Est. – estimate; *p* – *p*-value; Gender.comp. – gender composition of community Reference group for Gender composition: female Gray cells: *p*-values Bold font: *p*-values =< 0.10 Underlined and bold – *p*-values =<0.10 after correction for multi-testing (false discovery rate)

## Footnotes

1 However, in the cited research the study participants were adults and there is a possibility that findings may not apply to adolescents.

2 For instance, Walktrap function in R (cluster_walktrap) returns the membership vector based on the cut of the dendrogram corresponding to the highest modularity value, although it usually is not considered as modularity-based algorithm (in Smith et al.,2020).

3 Edge-betweenness optimizes the betweenness centrality of edges within a network to identify communities.

4 The size is grouped among the compositional properties in order to distinguish between non-network and network-based properties of communities.

5 To remind the reader of this we will write (-) after Substance Use, and (+) after Mental Wellbeing, to signify that higher value mean lower substance use and higher mental wellbeing.
