# Supplementary material for: Cross-sectional social network study of adolescent peer group variation in substance use and mental wellbeing
Source: Soc Networks. Author manuscript; Available in PMC 2025 Apr 4. (PMC7617561; doi:10.1016/j.socnet.2023.12.002)
Supplement: Supplementary Materials [file EMS203567-supplement-Supplementary_Materials.pdf]

Cross-sectional social network study of adolescent peer group variation in substance use and mental wellbeing: The importance of the meso-level

Supplementary Materials

Table of Contents

1 Dataset, missing data, and descriptive statistics ... 2
2 Principal component analysis ... 4
2.1 Raw scores and factor scores ... 5
3 Friendship networks in 22 schools ... 6
4 Community properties ... 8
4.1. Descriptives of community properties (Walktrap) ... 11
5 Multi-level models ... 13
5.1 Pair-wise model comparisons ... 18
5.2 Comparison of all models ... 19
5.3. Diagnostics of Model 3 (Walktrap) ... 20
5.4 Bivariate regression: level 1 and level 2 covariates and health outcomes ... 22
6 Group detection methods (GDMs) ... 23
***Table S13. Short description of all GDMs used in the study* ... 25**
6.1. Spearman correlations of the two outcomes and each community property ... 34
6.2. Bivariate relationship between six community properties and two health outcomes, controlling for community membership (as random effect) for 10 GDMs ... 37
6.3. Checking the existence of non-linear relationship between community properties and two health outcomes ... 40
6.4. Sensitivity of effects of community properties on two health outcomes to group detection method 44
7 Robustness checks ... 50
7.1 Raw scores and factor scores for Substance use and Mental wellbeing as outcome ... 50
7.2 Differences between schools with high and low response rate (all GDMs) ... 52
8 Two health outcomes and Walktrap communities ... 53
9 References ... 62

1 Dataset, missing data, and descriptive statistics

Table S1. Table showing number of students having data about friendships within school year (network data) and attribute data (3194 students participated in the study)

Students	***N***	***%***
Attribute data (participants in the study)	3194	*86.4*
Network data and attribute data (“non-isolates”)	3148	*85.2*
Partial* network data (no attribute data, non-participants)	501	*13.6*
Any network data	3649	*98.8*
**Total:** Any data (network or attribute)	**3695**	***100%***

* Contains only information about in-going ties

501 students who did not participate in the study had network data about their in-going ties from other students who participated in the study. 46 of 3194 students that participated in the study did not nominate anyone in their school year as a friend and were not nominated by anyone (“isolates”, not included in the following analyses).
Not all students participating in the survey had all attribute data used in the main analyses (see Fig S1).

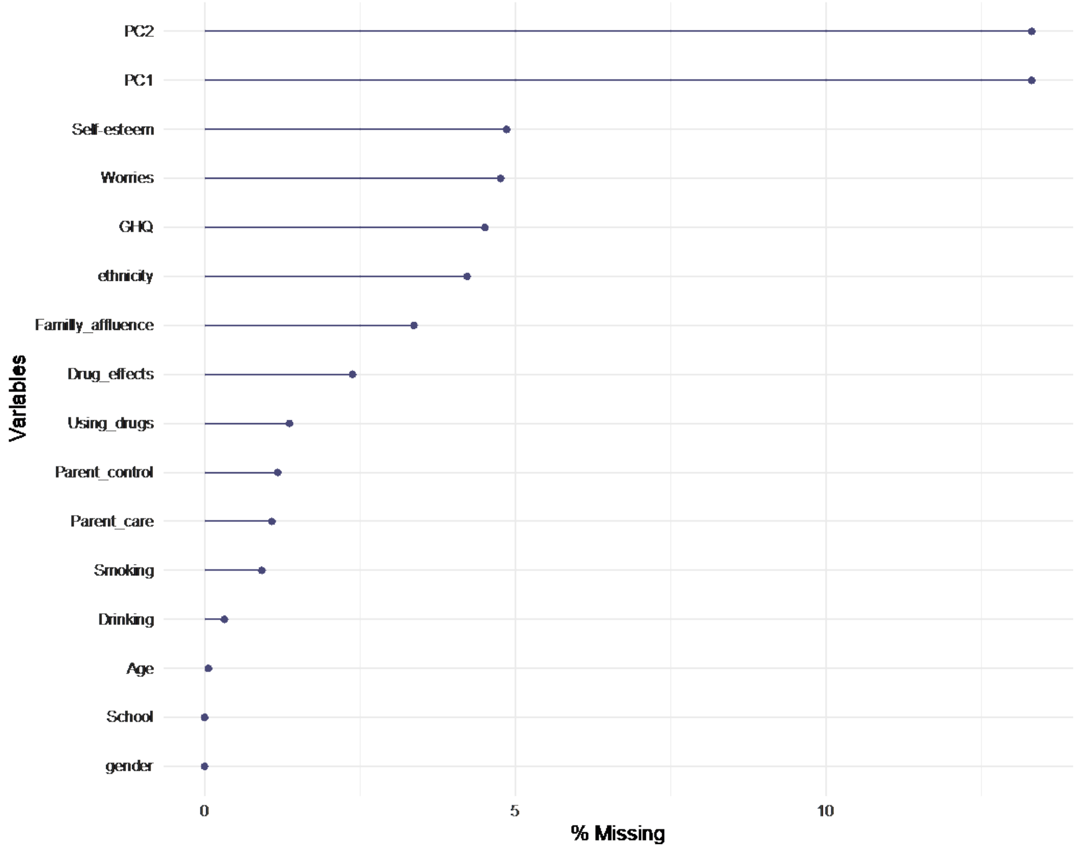

PC2, PC1 – principal component scores for the second (Mental Wellbeing) and first (Substance Use) component, respectively; GHQ – score on General Health Questionnaire

Fig S1. Percentage of missing data for single attributes (variables) for students participating in the study (*N*=3 194; x-axis: percentage of missing data; y-axis: attributes/variables used in the study)

Descriptive statistics for continuous variables in the study are shown in Table S2.

Table S2. *Means and standard deviations of continuous measures (N = 3194)*

Variables	*N*	*M*	*SD*	*Skewness*	*Kurtosis*
Parental control	3155	2.12	1.54	0.71	0.409
Parental care	3159	6.44	1.511	-0.962	0.662
Health-related variables					
Smoking	3164	2.29	1.501	0.933	-0.635
Drinking	3184	4.87	1.615	0.391	-0.649
Using drugs	3149	0.37	0.608	1.390	0.821
Drug effects	3115	14.76	0.948	-6.037	46.886
Self-esteem	3034	19.78	4.475	-0.367	0.733
General mental health	3044	11.07	5.477	0.962	1.021
Worries	3036	21.10	4.121	-0.177	-0.472

N -– number of all cases with no missing data; *M* – mean; *SD* – standard deviation

We imputed values for students who missed some of the data on attributes although they participated in the study. To impute the data, we used all other attributes included in Fig S1, but we did not include any network data (R package mice (Van Buuren, & Groothuis-Oudshoorn, 2011), 40 iterations, one imputation).

2 Principal component analysis

Fig S2 shows Pearson's correlations between seven health-related variables.

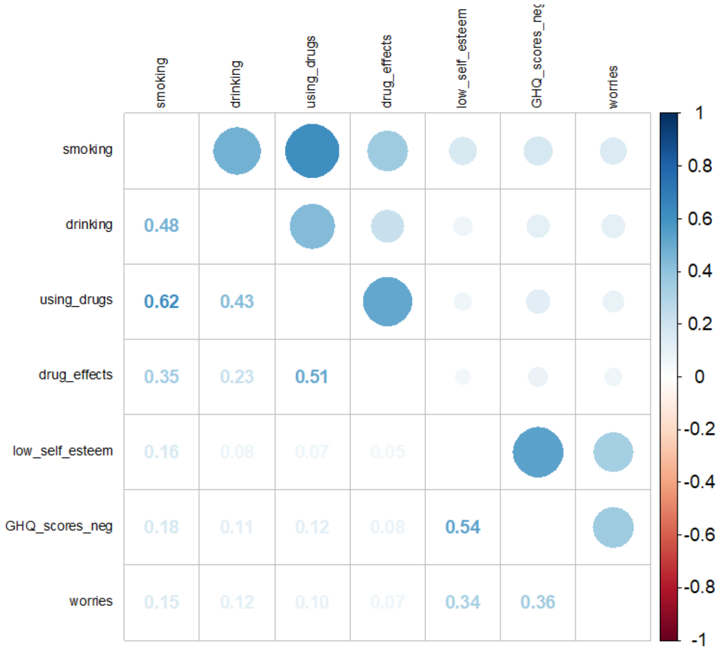

Fig S2. Pearson's correlations between seven health outcomes (*N* = 3148, non-imputed data, pair-wise complete observations; all variables are coded so higher values signify a more negative outcome)

Health outcomes: Substance Use (SU) and Mental Wellbeing (MW)

We aimed to create not correlated health outcomes and wanted to use a high percentage of variance in seven input variables. Therefore, we opted for a more complex weighting scheme for seven items to arrive at two component scores for each individual. Thus, SU scores include positive weights for items related with mental wellbeing, while MW scores include negative weights for items related with substance use (see Table S3 below). That is, in our sample, students who used substances more tended to have worse mental wellbeing, whilst students who had better mental wellbeing tended not to use drugs.

Table S3. Weights of seven health related variables for two principal components (N = 2758, cases with complete data)

Variable	*PC1 weights Substance use*	*PC2 weights Mental wellbeing*
Smoking (-)	0.31	-0.15
Drinking (-)	0.25	-0.16
Using drugs (-)	0.31	-0.23
Drug effects (-)	0.23	-0.20
Low Self-esteem (-)	0.17	0.43
GHQ (-)	0.19	0.41
Worries (-)	0.16	0.34

(-) – all variables are recoded in the same direction to facilitate the interpretation of weights, higher score meaning more negative outcome (e.g., more drug use, lower self-esteem)

2.1 Raw scores and factor scores

Raw scores are calculated by summing standardized values[1] for everyone as follows:
$SU =$ Smoking + Drinking + Drug use + Drug effects
$MW =$ Self-esteem + General mental health (GHQ) + Worries
In contrast with principal component and factor scores, only behaviours and outcomes on which the highest loadings are found for the two identified components are used for the calculations.
Factor scores are based on factor analysis (orthogonal, factors with oblique rotation were correlated below 0.3). Since, the two factors together explain 46% of variance (in comparison to two principal component scores that explain 60%) and had scores with higher skewness than principal components, we decided to use principal component scores.
Two principal component scores are highly correlated with both row scores (Spearman correlations: 0.88; 0.77, non-imputed dataset $N = 3148$, for Substance use and Mental wellbeing, respectively) and factor scores (Spearman correlations: 0.98; 0.95, non-imputed dataset $N = 3148$, for Substance use and Mental wellbeing, respectively).

[1] Technically, the values are not "raw" since we standardized them. We use the adjective "raw" to highlight that they are based on simpler calculations.

3 Friendship networks in 22 schools

Descriptives of some network properties of 22 schools are shown in Table S4.

Table S4. Basic network descriptives of 22 schools

School	*1*	*2*	*3*	*4*	*5*	*6*	*7*	*8*	*9*	*10*	*11*	*12*	*13*	*14*	*15*	*16*	*17*	*18*	*19*	*20*	*21*	*22*
N net. (without isolates)	115	113	173	190	145	73	348	132	218	162	159	115	263	282	57	278	107	132	86	143	222	136
N isolates	1	0	2	8	1	3	4	1	0	2	2	2	0	4	1	2	6	0	2	1	2	2
% Non respond.	12.2	12.4	17.9	22.1	15.2	28.8	18.1	9.8	8.7	19.8	12.6	13.9	12.5	10.6	7.0	10.8	23.4	14.4	10.5	11.2	10.4	3.7
Density	0.04	0.03	0.02	0.01	0.03	0.04	0.01	0.03	0.02	0.02	0.02	0.03	0.02	0.02	0.07	0.01	0.02	0.03	0.05	0.03	0.02	0.03
Recipro-city	0.67	0.58	0.49	0.47	0.58	0.58	0.55	0.62	0.56	0.55	0.52	0.60	0.56	0.59	0.60	0.56	0.50	0.53	0.62	0.62	0.60	0.6
Transit-ivity	0.49	0.43	0.42	0.40	0.40	0.50	0.37	0.34	0.35	0.39	0.46	0.42	0.33	0.34	0.43	0.39	0.42	0.38	0.42	0.51	0.41	0.33
% Big. comp.	93.9	98.2	97.1	90	98.6	90.4	98.3	100	100	98.8	95	98.3	100	100	93	99.3	95.3	100	95.3	96.5	100	97.8
Centrali-zation	0.036	0.035	0.027	0.031	0.038	0.054	0.014	0.044	0.027	0.025	0.031	0.059	0.023	0.02	0.06	0.022	0.034	0.044	0.069	0.034	0.025	0.035
Total degree	7.97	6.39	5.85	5.32	7.30	5.42	7.33	7.76	8.39	7.11	6.45	6.87	8.04	8.67	7.47	7.89	4.92	6.56	7.58	8.53	8.23	8.78
EI gender	-0.87	-0.94	-0.74	-0.82	-0.80	-0.89	-0.83	-0.70	-0.86	-0.76	-0.71	-0.74	-0.76	-0.76	-0.77	-0.83	-0.80	-0.88	-0.91	-0.95	-0.90	-0.75
Ethnicity (white)	0.99	0.99	0.99	0.85	0.97	0.98	0.86	0.98	0.85	0.90	0.99	0.99	0.90	0.96	0.95	0.92	0.74	0.96	0.88	0.97	0.89	0.89
Avg. FA	4.83	4.18	4.93	4.58	4.62	3.69	4.66	4.89	6.28	5.04	4.68	4.78	5.83	5.45	4.80	5.44	3.68	4.79	4.87	5.21	5.52	5.04
% Girls (net)	0.56	0.64	0.52	0.41	0.51	0.53	0.56	0.50	0.43	0.49	0.59	0.47	0.48	0.50	0.46	0.50	0.47	0.51	0.46	0.55	0.52	0.53

Abbreviations: net – network; %Big.comp. – the percentage of students in the big component of the network – the biggest connected part; EI gender – EI index for gender; Avg. FA – average family affluence; Numbers in columns: 22 schools

4 Community properties

The details about how we calculated six community properties of each peer group in the networks are provided in the text below.

Community size. The number of all students belonging to the community.

Community gender composition. Due to high gender homophily many communities will have only girls or boys as members. Therefore, each community is described as female, male, or mixed – if it had at least one member of the opposite gender.

Ratio of ties outside the community. Each member of a community can have ties with other members of the same community (inside community ties) and/or with members from other communities in the school (ties outside community). The ratio of ties outside communities is calculated by summing all outside community ties of all members and dividing it by the sum of all their (inside and outside community), expressed by formula: $\frac{\Sigma\ external\ community's\ ties}{\Sigma\ all\ (internal+external)\ community's\ ties}$. It is analogous to measure often used on whole network and all communities, known as mixing parameter (μ), but in our case it is applied to each community separately. Value of the ratio for communities with just one member is 1.

Transitivity. Transitivity measures the tendency of nodes to cluster together. There are several different versions of the measure. and we use so-called global transitivity. More specifically, it is based on triads – network subgraphs formed by three nodes. Transitivity means that if there is a tie between *i* and *j* and between *j* and *k*, there is also a tie between *i* and *k*, ignoring the direction of ties. It measures the relative frequency of triangles in the community. expressed by formula: $\frac{3*N\ of\ connected\ triads}{N\ of\ all\ connected\ triplets}$. where triplets are any two ties that share a node. Transitivity can theoretically vary from 0 to 1. and higher score means higher transitivity. Communities that have just one or two ties between members had no transitivity value of 0, while communities with no ties between members (possible to have in BIA approach) had no transitivity value (0).

Centralization. This measure quantifies variation in centrality scores among nodes in the network. We apply the measure to nodes' total number of ties (in-going and out-going). regardless of their direction and measure it at community level. The formula for centralization (Freeman, 1979) is:

$$C_D = \frac{\sum_{i=1}^{g}[C_D(m) - C_D(n_i\ )]}{[(g-1)(g-2)]}$$

where g is the number of nodes in a community *i* represents each node, m is the centrality value of the node with highest centrality in the community. This value is normalised by dividing by the theoretical maximum centralization score for a graph with the same number of nodes. In that way, centralization is the ratio of the actual sum of differences to the maximum possible sum of differences and it ranges from 0 to 1.

Hierarchy. We use Tau statistics constructed by McFarland et al. (2019) to capture hierarchical, vertical differentiation in the network. As transitivity, this measure is based on triads. but in difference with both transitivity and centralization it considers the direction of ties. Hierarchy exists when two individuals in the network nominate the same third individual, implying an over-representation of "up" pointing triads. In directed networks 16 types of triads are possible to occur. Their labels use the number of mutual, asymmetric, and null ties, followed by an abbreviation for direction (D – down; U – up; T – transitive; and C – cycle). This measure is based on the count of five of them that show some "status ordering" (021D, 021U, 030T, 120D, 120U), subtracted by the count of one so-called antithetical case (021C) that shows inconsistency in status ordering (see Fig S3). The ranked-clustering weighting scheme is built by Davis and Leinhardt (1972). The total score was divided by the sum of all triads in the community to make the scores comparable across different community networks. The higher occurrence of the specific types of directed triads (and lack of 021C) among all triads in the community (the higher the score) suggests a tendency toward hierarchy in the overall network (community in our case). Negative values in hierarchy were possible if 021C configuration was more frequent than all other hierarchical configurations (21D, 021U, 030T, 120D, 120U).

Triad label	*021D*	*021U*	*030T*	*120D*	*120U*	*021C*
Triad	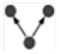	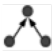	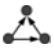	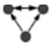	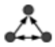	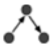
Weights	1	1	1	1	1	-1

Fig S3. Triads weighted for Tau (hierarchy) score

The centralization and hierarchy scores cannot be calculated for communities that have less than three members (to see examples on networks (communities) with two to five nodes (members), see Table S5).

Table S5. Examples of transitivity. centralization related and hierarchy values for small communities

	Example	Transitivity	Centralization	Hierarchy (simple score, not normalised)
1	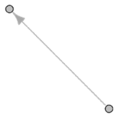	NA	NA	NA
2	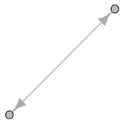	NA	NA	NA
3	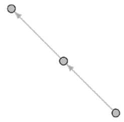	0	2	-1
4	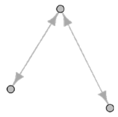	0	4	0
5	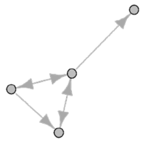	0.6	8	0
6	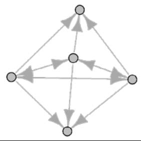	0.86	6	9

4.1. Descriptives of community properties (Walktrap)

Descriptives of community properties for Walktrap algorithm are shown in Table S6, and the scatterplot showing correlations between community properties and two health outcomes (mean of the group), and their distributions is shown in Fig S4. Since Gender composition is a nominal property, a variable called G.C.n (Gender composition - numeric) in which mixed groups were assigned with value 0, females and males with 1 and -1 is used in Fig S4, respectively. The correlation values are Pearson's correlations. Five community properties show a variation in values across communities (Table S6). Most communities are female (40.3%) or male (38.2%), but 21.4% are mixed in gender.

Table S6. Descriptives of five continuous community properties for Walktrap algorithm (N = 387)

Community property	N	Mean	Median	SD	Min.	Max.	Range	Skew.	Kurtosis
Community size	387	9.43	7	7.22	1	44	43	1.68	3.55
Ratio of outside community ties	387	0.29	0.29	0.16	0	1	1	0.4	0.77
Transitivity	352	0.58	0.59	0.26	0	1	1	-0.5	0.23
Centralization	339	0.28	0.25	0.15	0	1	1	1.35	4.04
Hierarchy	339	0.16	0.07	0.24	-0.25	1	1.25	2.03	3.9

N – non-missing data; SD – standard deviation; Skew. – skewness; Prop. F – proportion of females in community

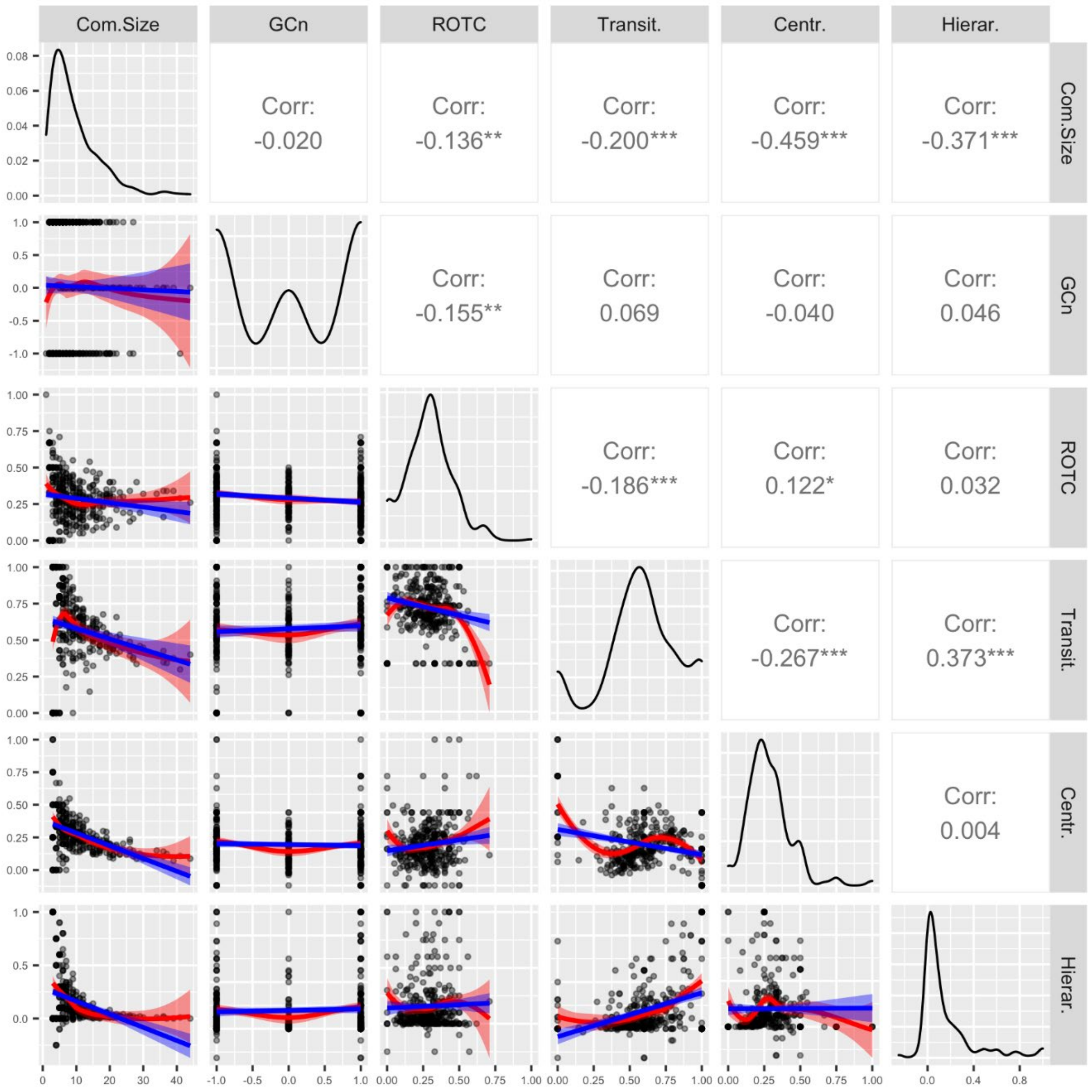

Com.size – community size; GCn. – gender composition as numeric variable: 1 is assigned to female, 0 to mixed and -1 to male; ROTC – ratio of ties outside the community; Tran. – transitivity; Centr. – centralization; Hier. - hierarchy

Fig S4. Scatterplots. distributions. and Pearson correlation coefficients between Walktrap's community properties (unit of analysis is community, *N*=387). In scatterplots: blue – linear trend based on linear regression; red – non-linear trend based on local polynomial regression fitting.

Bigger communities are less centralized, have less hierarchy and are less transitive. Additionally, the smaller proportion of their ties is outside communities. Peer groups made of boys are more open – have a higher ratio of ties with members of other

communities. More open communities are less transitive and show a tendency to be more centralized. More transitive communities have a higher hierarchy, but are less centralized.

5 Multi-level models

Boxplots in Fig S5 show distributions of Substance Use and Mental Wellbeing in 22 schools.

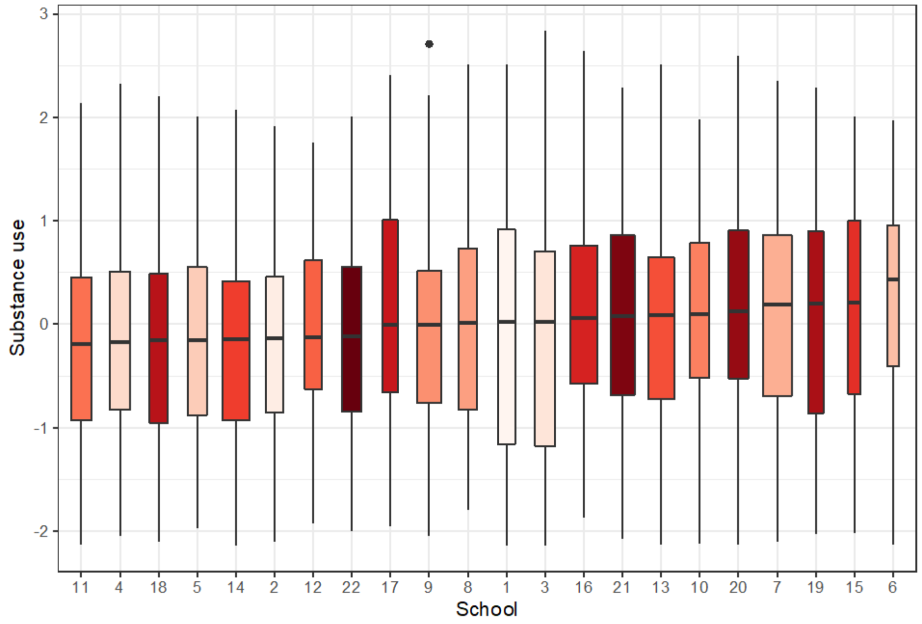

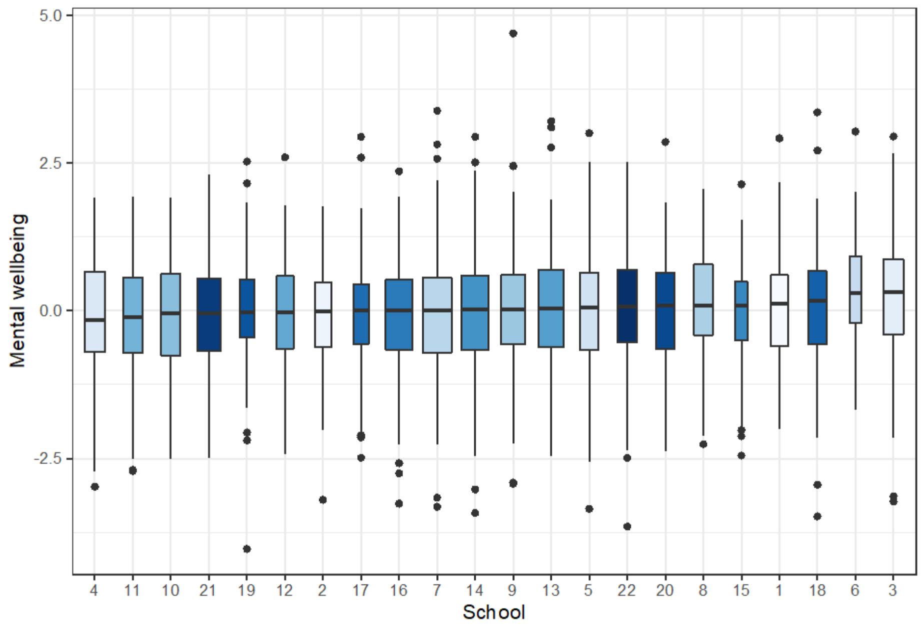

Fig S5. Boxplots for Substance use (top) and Mental wellbeing (bottom) by 22 schools (*N*= 3148, imputed dataset)

Caterpillar plots in Figs S6 and S7 show variation in two health outcomes in peer groups (communities) in schools.

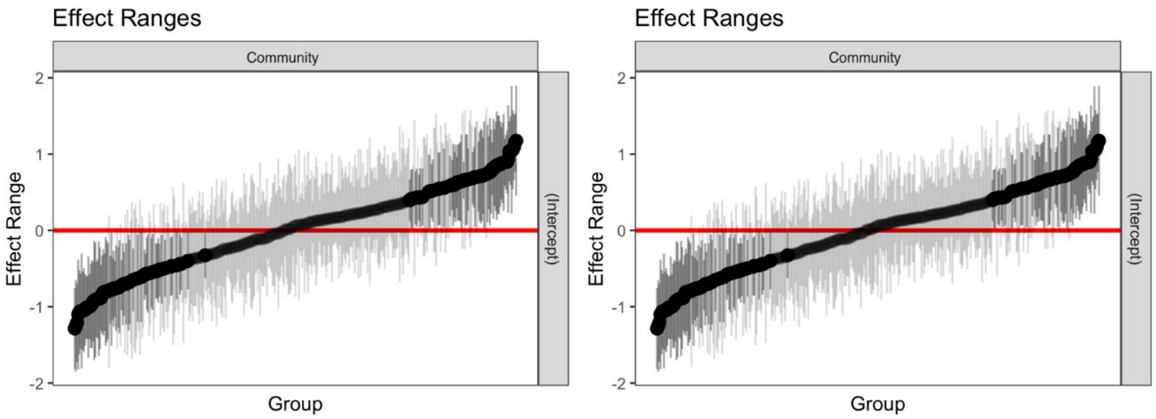

Fig S6. Caterpillar plots of random effects for Substance use (left) and Mental wellbeing (right), Model 1

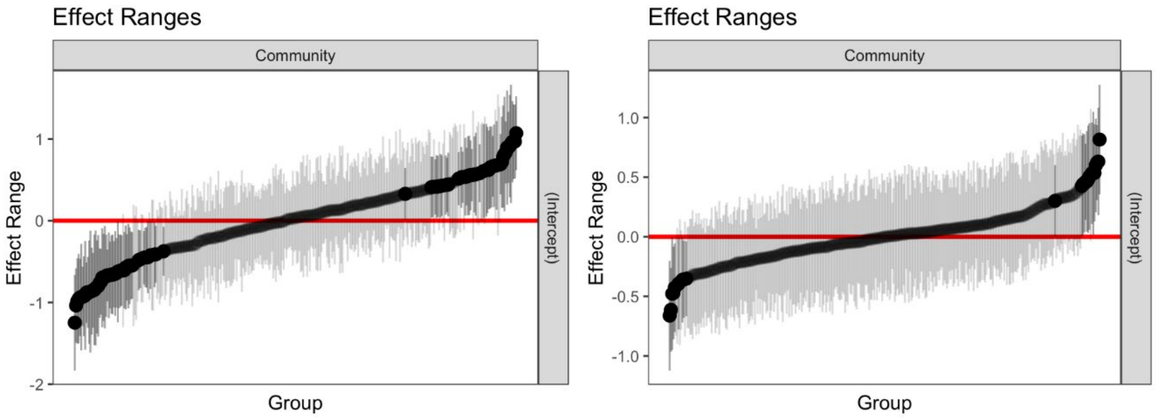

Fig S7. Caterpillar plots of random effects for Substance use (left) and Mental wellbeing (right), Model 3

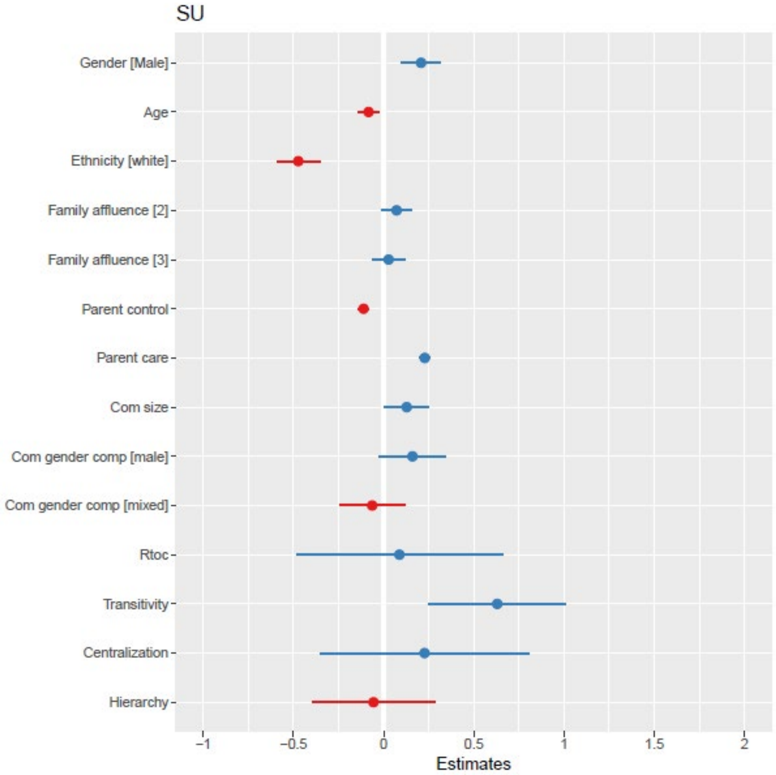

Fig S8. Plot of fixed effects of Model 3 for Substance Use

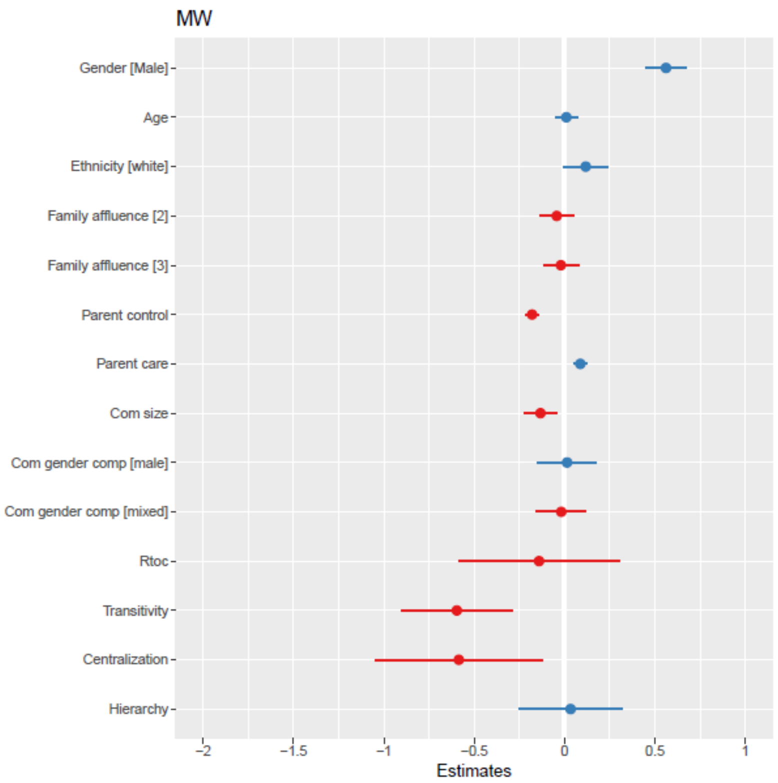

Fig S9. Plot of fixed effects of Model 3 for Mental Wellbeing

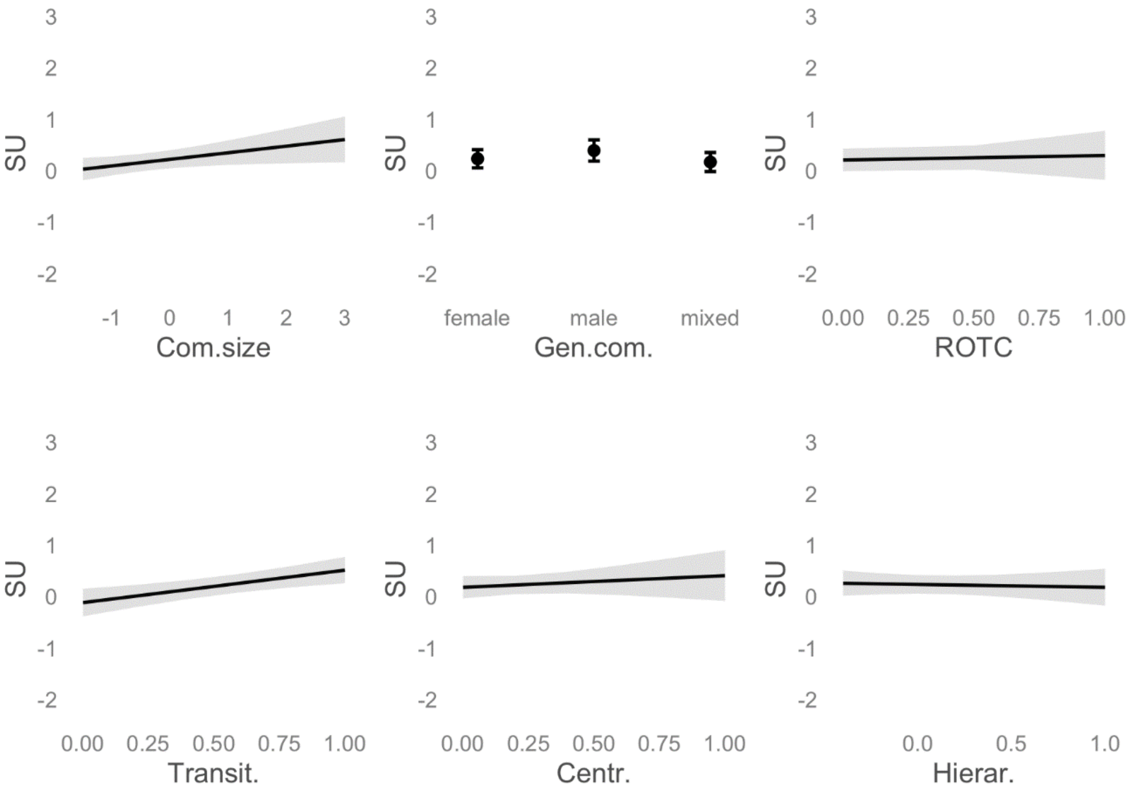

Fig S10. Predictions of Substance use (x-axis) based on Model 3 by varying the community property (y-axis, the focal variable) and holding all other community properties and level 1 covariates (non-focal variables)

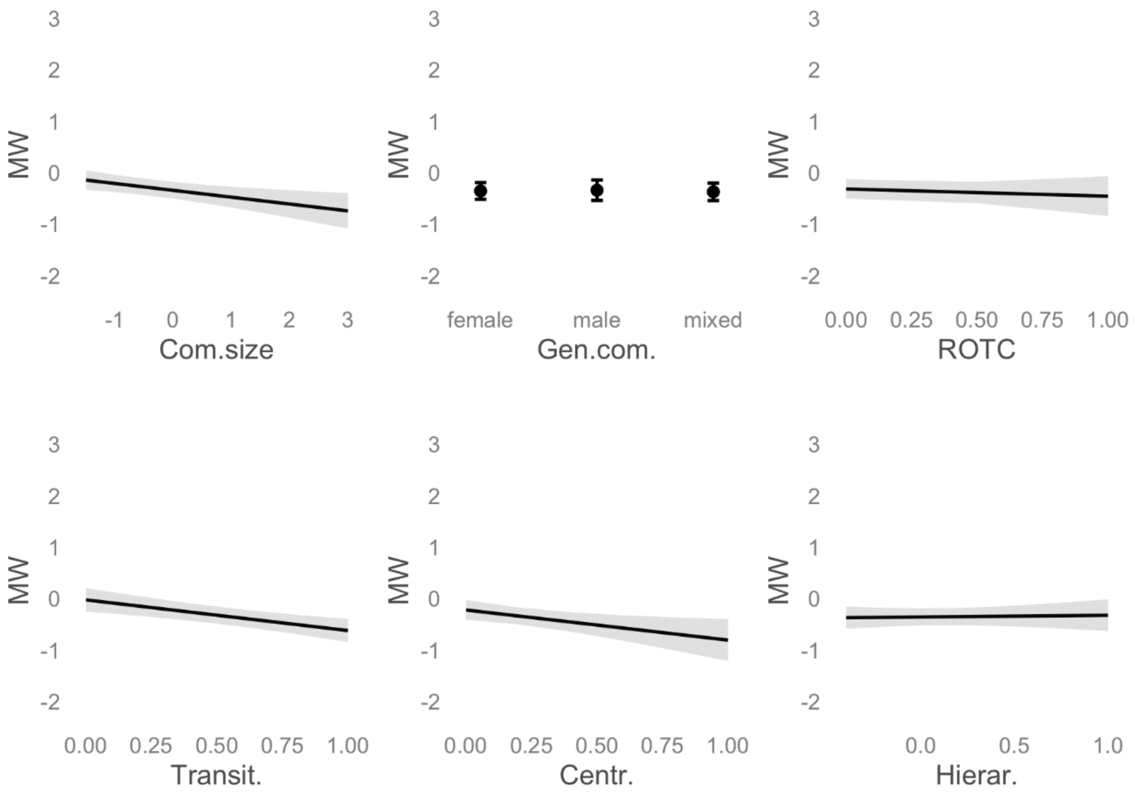

Fig S11. Predictions of Mental wellbeing (x-axis) of Model 3 by varying the community property (y-axis, the focal variable) and holding all other community properties and level 1 covariates (non-focal variables)

5.1 Pair-wise model comparisons

Each progressively more complex model was compared with the previous model to gauge whether the fit is significantly better (ANOVA F-test).
Results demonstrate (Table S7) that for both outcomes, Model 1.1 that included schools as a level within which communities are nested and Model 1 which included only community level, did not differ in how they fit the data.

Table S7. P-values of comparisons between different pairs of models

Dependent variable	Substance use	Mental wellbeing
Compared models	χ^2 p	χ^2 p
M1 & M1.1	1	1
M1 & M2	p<0.001	p<0.001
M2 & M3	0.966	1
M3 & M4	1	1

5.2 Comparison of all models

We compared all six models, for each outcome separately, using performance R package (Lüdecke et al., 2021). Using *compare_performance* function allowed us to assess model fit and rank them from the best to the worst based on five indices: R^2 (adjusted R squared), ICC (adjusted intraclass correlation coefficient), RMSE (root-mean-square error), AIC (Akaike information criterion), Sigma (residual standard error) and BIC (Bayesian information criterion). Based on those indices, Performance Score is calculated for both health outcomes and Model 3 (Tables S8 and S9) is ranked as the best model (for more details on the exact procedure see Lüdecke et al., 2021).

Table S8. Ranked models – Substance use

Model	R^2 conditional	R^2 marginal	ICC	RMSE	Sigma	AIC	BIC	Performance Score
M3	0.41	0.14	0.32	0.73	0.76	0.96	1.00	0.85
M4	0.41	0.14	0.32	0.73	0.76	0.04	0.00	0.58
M2	0.41	0.12	0.33	0.73	0.76	0.00	0.00	0.56
M1	0.37	0.00	0.37	0.77	0.81	0.00	0.00	0.14

Abbreviations: For the meaning of the acronyms in the first row, see the text above Table S8.

Table S9. Ranked models – Mental wellbeing

Model	R^2 conditional	R^2 marginal	ICC	RMSE	Sigma	AIC	BIC	Performance Score
M3	0.26	0.15	0.13	0.82	0.84	0.98	1.00	0.84
M4	0.26	0.15	0.14	0.82	0.85	0.00	0.00	0.57
M2	0.26	0.15	0.13	0.82	0.84	0.02	0.00	0.57
M1	0.19	0.00	0.19	0.86	0.89	0.00	0.00	0.14

Abbreviations: For the meaning of the acronyms in the first row, see the text above Table S8.

5.3. Diagnostics of Model 3 (Walktrap)

Variation inflation factors for all variables in model 5 (both outcomes) are shown in Fig S12 and S13.

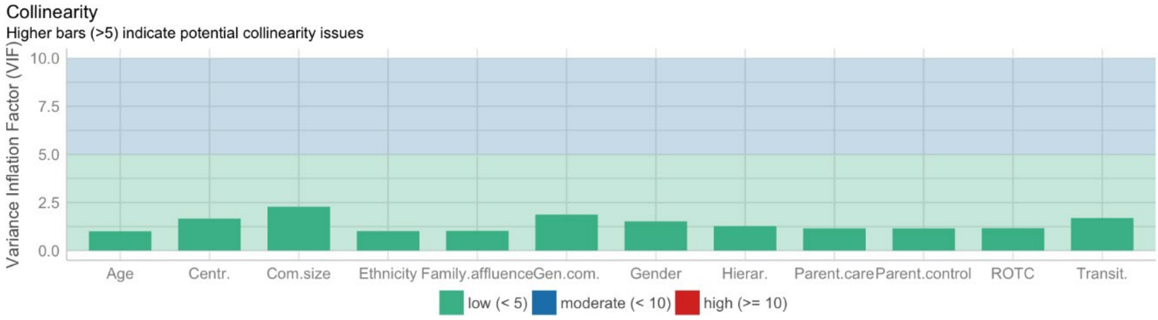

Fig S12. Variation inflation factors (Model 3, Dependent variable: Substance use)

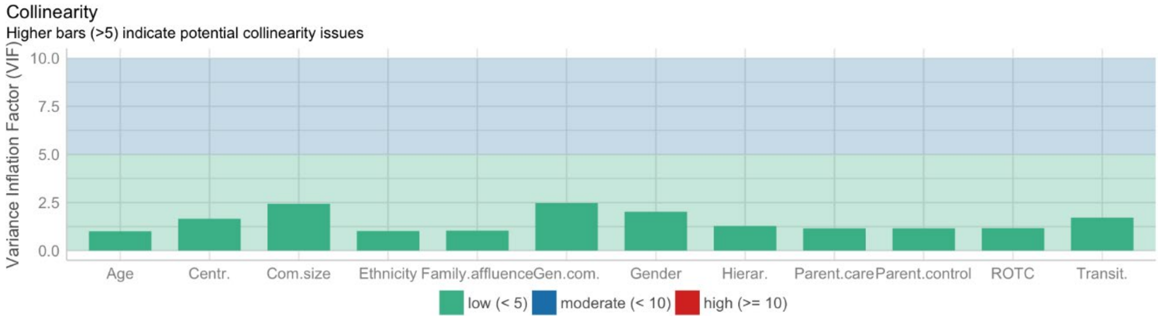

Fig S13. Variation inflation factors (Model 3, Dependent variable: Mental wellbeing)

We performed model diagnostics for Model 3 (GDM: Walktrap) for both dependent variables to check if assumptions required for multilevel modelling are violated. We tested normality of residuals at level 1 and 2, heteroscedasticity, existence of outliers, and autocorrelation (check_model function in performance R package: Ludecke at al., 2021). The assumption of homoscedascity is met for Substance Use (Lavene's test, $F = 1.27$; $p = 0.26$) and for Mental Wellbeing ($F = 0.69$; $p = 0.41$).

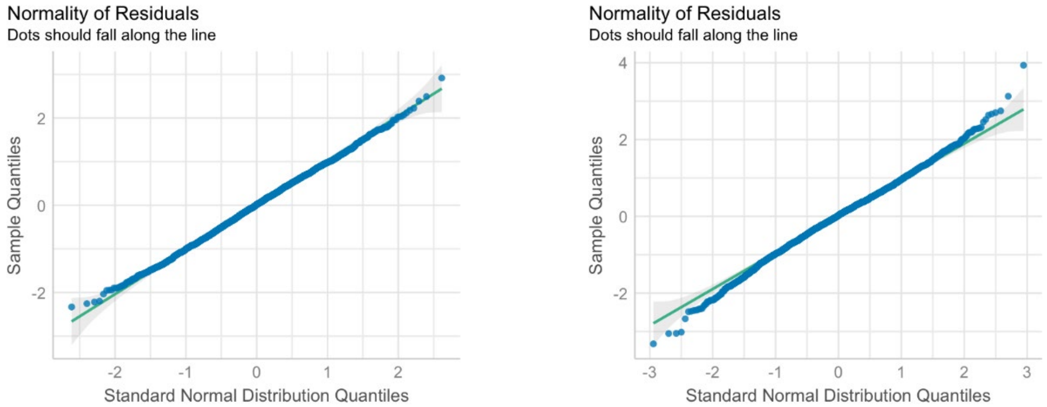

Fig S14. Normality of residuals for Substance use (*p* = 0.309) and Mental Wellbeing (*p*< .001, non-normality detected)

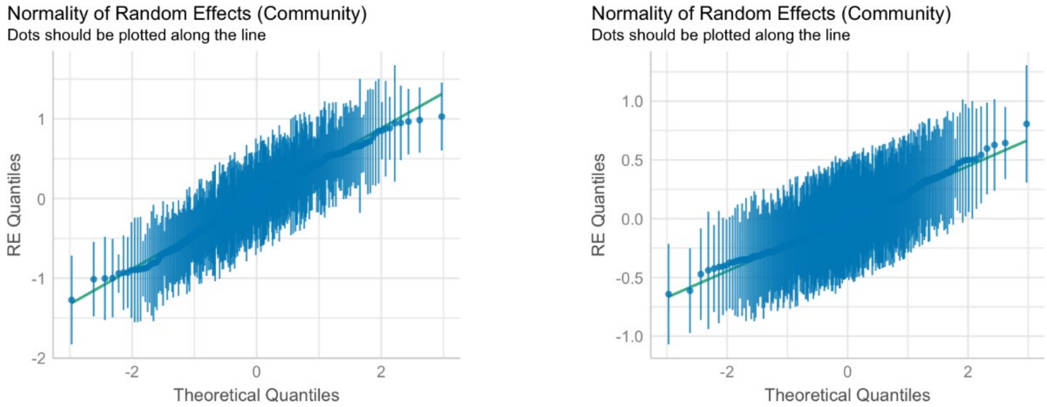

Fig S15. Normality of random effects for Substance use (*p* = 0. 073) and Mental Wellbeing (*p* = 0. 018, non-normality detected)

5.4 Bivariate regression: level 1 and level 2 covariates and health outcomes

Table S10. Bivariate regression coefficients between six individual variables and two health outcomes

Outcome	*Substance Use*		*Mental Wellbeing*	
Predictor	*Estimate*	*p*	*Estimate*	*p*
Gender - male	0.41	<0.001	0.59	<0.001
Age	-0.11	0.001	0.03	0.454
Ethnicity	-0.57	<0.001	0.23	<0.001
Family affluence - medium	0.1	0.061	-0.01	0.78
Family affluence - high	0.18	0.001	0.05	0.345
Parental control	-0.22	<0.001	-0.23	<0.001
Parental care	0.3	<0.001	0.15	<0.001

Table S11. Bivariate regression coefficients between six individual variables and two health outcomes, controlling for peer group membership (as a random effect)

Outcome	*Substance Use*		*Mental Wellbeing*	
Predictor	*Estimate*	*p*	*Estimate*	*p*
Gender - male	0.29	<0.001	0.59	<0.001
Age	-0.1	0.001	0	0.988
Ethnicity	-0.39	<0.001	0.19	0.005
Family affluence - medium	0.07	0.107	-0.03	0.509
Family affluence - high	0.07	0.134	0.03	0.579
Parental control	-0.19	<0.001	-0.22	<0.001
Parental care	0.26	<0.001	0.17	<0.001

Table S12. Bivariate relationship between six community properties and two health outcomes, controlling for Walktrap peer group membership (as random effect)

Outcome	*Substance Use*		*Mental Wellbeing*	
Predictor	*Estimate*	*p*	*Estimate*	*p*
Community size	0.05	0.269	0.01	0.843
Gender comp.- male	0.35	<0.001	0.6	<0.001
Gender comp.- mixed	0.05	0.618	0.2	0.002
ROTC	-0.11	0.679	0.59	0.007
Transitivity	0.31	0.047	-0.31	0.019
Centralization	-0.25	0.336	0.01	0.954
Hierarchy	-0.02	0.925	-0.1	0.479

It is worthwhile noting that when only one community property is included in a multi-level model with peer groups (communities) as random effects (Table S12) effects remain mostly similar. The notable exception is community size, which is not significant and has the same (positive) direction for both outcomes when other community properties are not accounted for.

6 Group detection methods (GDMs)

We started with the ensemble of methods available in R software. All methods used are available in igraph package (Csardi & Nepusz, 2006), except for CP and BIA. CP is available in clique percolation package (Lange, 202), but we slightly modified the original code so that it can handle network matrices as input data and provide results for non-weighted networks. BIA method consisted of several steps described in Table 12. For choosing the optimal number of clusters for each network for BIA method, we used *clusterboot* function from fpc R package (Hennig & Imports, 2015). Igraph package also includes Spinglass, Fluid communities and Leading eigenvector, but we have not used them since they require the input network to be connected, which was not the case for 17 out of 22 school networks. For SBM, we used blockmodels R package (Leger, 2016).
As Table 12 shows. CP, FG, LE, LO, and LP algorithms are implemented only for undirected networks. Therefore, we used the undirected version of original networks for these algorithms. Specifically, we symmetrized networks with so-called "weak rule". Weak rule means that the information about directionality of ties is disregarded – both mutual and non-mutual ties are treated equally, as an undirected tie. In other words, if student A nominated student B as their friend, but B did not nominate A, this tie is treated equally as if A and B nominated each other.

We applied ten GDMs to friendship networks of 22 schools. Fig S16 and S17 illustrate different partitions of the friendship network for one school. For visualizations, we chose schools with relatively smaller number of students, so that partitions are easier to see in visualizations. Note that all GDMs find exclusive communities (where nobody is a member of two or more groups). CP originally gives overlapping communities, but all students that were assigned to more than one group are placed in the community with which they had the most ties. Overlap between colours of different communities is result of the node placement in the plots. Isolates are not shown in Figs S16 and S17.
A short description of ten GDMs is provided in Table S13 (partly based on Smith et al., 2020).

Table S13. Short description of all GDMs used in the study

**Algorithm (abbreviation)**	**Directed**	**Basic logic**	Tuning parameters	Possible use
Blockmodeling – indirect approach (BIA)	Yes	Identifies groups of nodes with similar position and profile of ties to others. Based on the notion of structural equivalence (Batagelj et al., 1992).	Similarity measure based on profile of in and out going ties; partition is done with hierarchical clustering (average method); number of clusters for each school/network is based on combination of indices: average Jaccard similarity and Instability (1000 bootstrap samples), and R^2.	When not interested in the social influence within a community, but rather in different social positions and roles in the network.
Clique Percolation (CP)	No	Starts with identifying k-cliques, which are fully connected networks with k nodes. A community is defined as a set of adjacent k-cliques that share exactly k−1 nodes. With k=3, two 3-cliques are adjacent if they share exactly two nodes (equivalent to an edge). A node can belong to more than one community (Palla et al., 2005)	Cliques of size 3 are considered (González et al., 2007).	When interested in social influence for which tight, small communities with possibly structurally strong ties are supposed to be relevant and there is no emphasis on minimising outside community ties.
Edge-betweenness (EB)	Yes*	Gradually removes the edges with the highest edge betweenness score (Newman & Girvan, 2004).	For directed networks. Directed paths are considered when determining the shortest paths.	When the interest is in identifying edges that are the most crucial for transmission – the ones that have the highest edge-betweenness score and are between communities
Fast-greedy (FG)	No	Tries to find dense subgraphs in graphs via directly optimising the modularity score (Clauset et al., 2005).	None/default settings	
Infomap (IM)	Yes	Finds community structure by simulating the flow of information through a network that minimises the expected description length of a random walker trajectory (Rosvall & Bergstrom, 2007).	The number of attempts to partition the network is set to 10.	Questions about transmission of information, behaviours as simple contagion because it defines communities on basis of flow
Leiden (LE)	No	Similar approach to Louvian method, but with the goal of identifying well-connected communities (Traag et al., 2019).	Objective function is set to "modularity"; resolution parameter = 1; beta = 0.01; number of iterations = 2; initial membership is not provided.	When interested in processes within communities and not between them, LO and LE are good choices because they minimise the outside community connections and maximise inside community connections
Louvain (LO)	No	Based on the modularity measure and a hierarchical approach. In every step, vertices are re-assigned to communities in a local, greedy way: each vertex is moved to the community with which it achieves the highest contribution to modularity (Blondel et al., 2008).	None/default settings	

Label propagation (LP)	No	Starts with random assignment of labels to vertices, and keeps reassigning the labels iteratively based on the labels of nearest neighbours until reaching convergence (Raghavan et al., 2007).	None/default settings	Questions about adoption of social norms because it is based on the processes of iterative adoption
Stochastic blockmodeling (SBM)	Yes	Identifies groups of nodes with similar position. Based on the notion of regular equivalence (Kolaczyk & Csárdi, 2014).	Performs estimation of blockmodels for bernoulli probability distribution, verbosity = 3; exploration factor = 5.	When not interested in the social influence within a community, but rather in different social positions and roles in the network.
Walktrap (WT)	Yes	Finds densely connected communities in a graph by simulating the path of a random walker over time. The idea is that short random walks tend to be trapped in the same community (Pons & Latapy, 2005).	The length of random walk to perform is set to 4.	Research questions about transmission of information, behaviours as simple contagion because it defines communities on basis of flow

* The function *cluster_edge_betweenness* in igraph R package calculates directed edge betweenness for directed graphs.

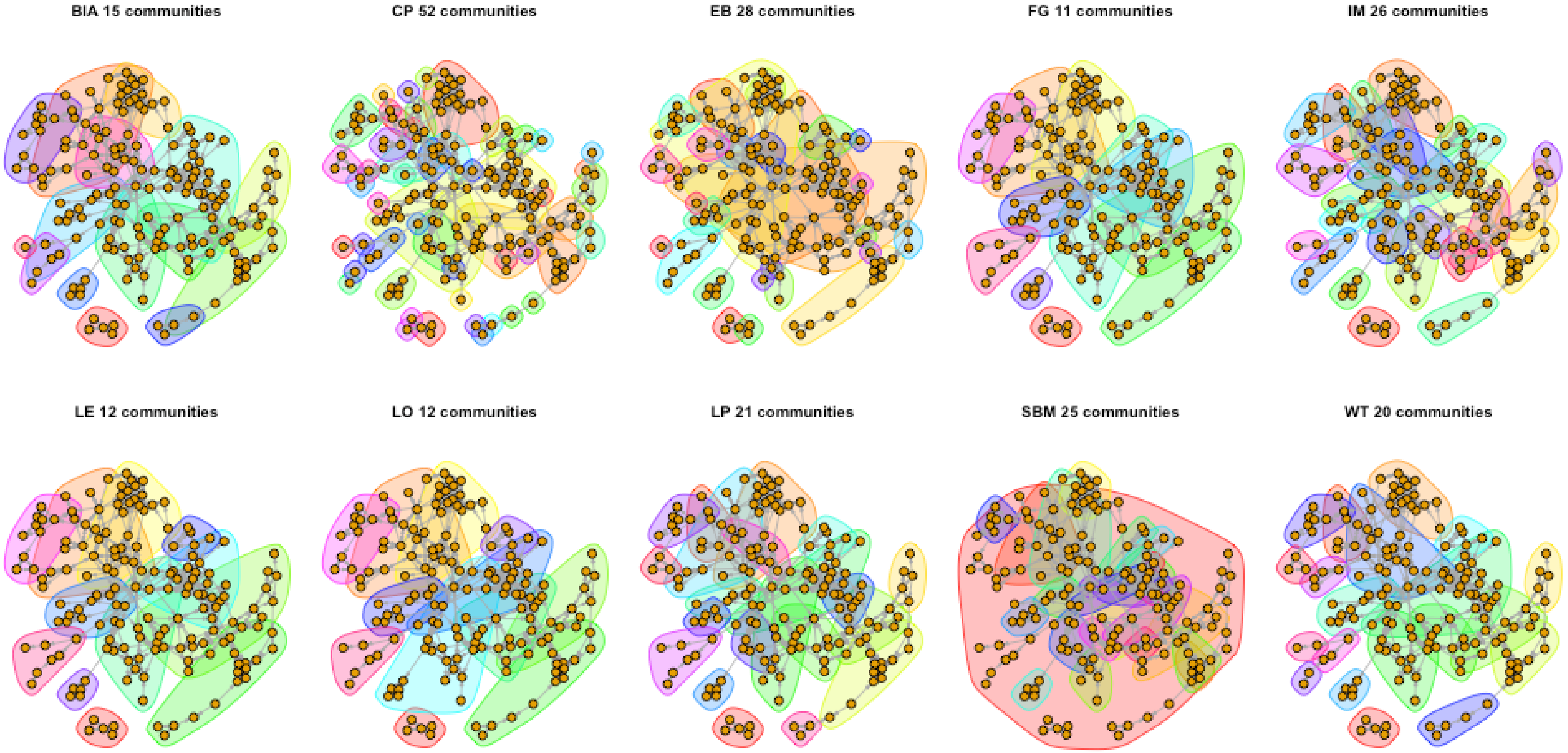

Fig S16. Partitions of ten GDMs for school 3 (*N* = 73; Non-responders = 18%)

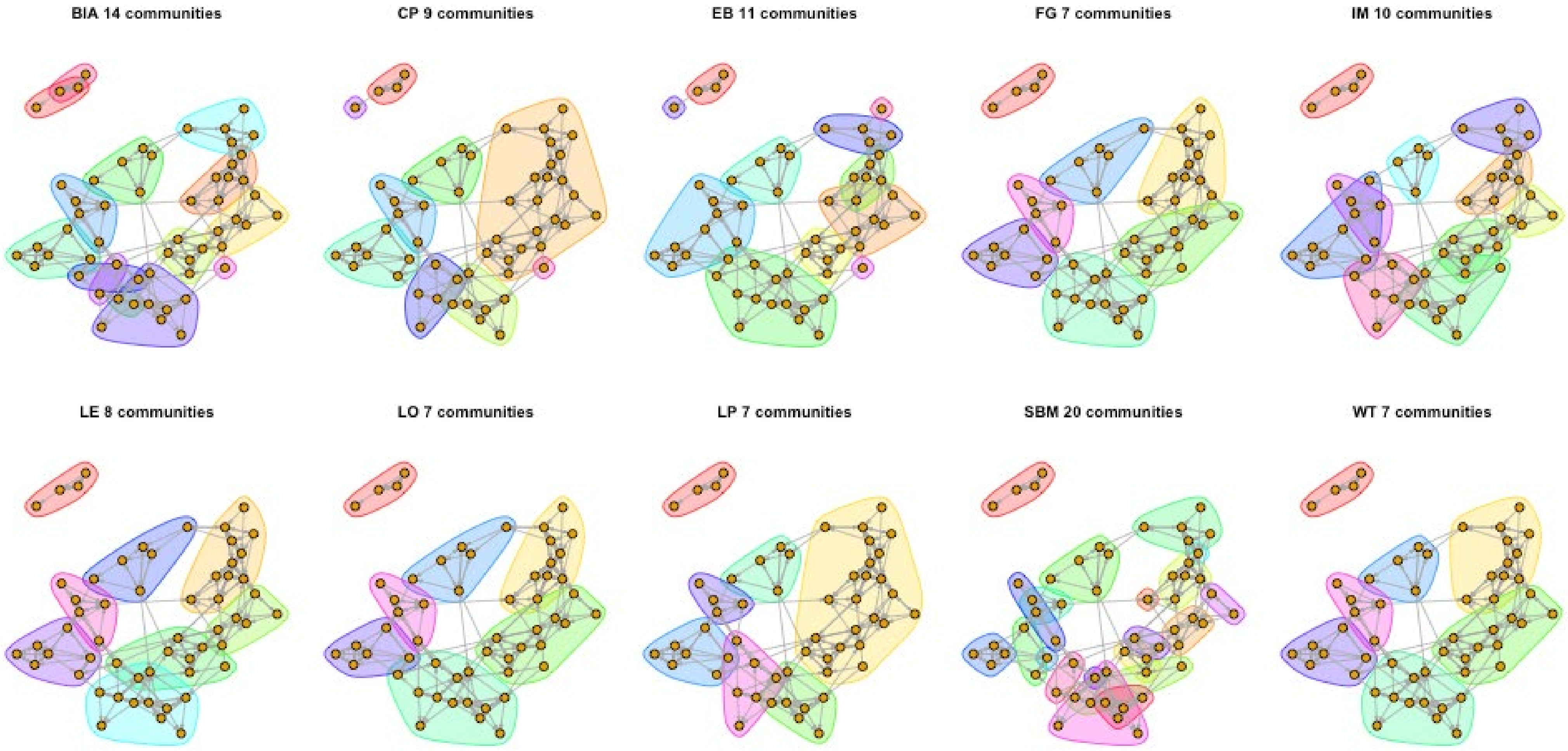

Fig S17. Partitions of ten GDMs for school 15 (N = 57; Non-responders = 7%)

Table S14. Group detection methods and communities found in 22 schools

GDM	BIA	CP	EB	FG	IM	LE	LO	LP	SBM	WT
N Com.	300	895	546	235	525	252	253	401	680	387
Avg. com. size	12.16	4.08	6.68	15.53	6.95	14.48	14.42	9.1	5.37	9.43
Min. com. size	1	1	1	2	1	2	2	1	1	1
Max. com. size	62	98	106	81	28	45	41	40	43	44
N size 1	37	514	214	0	5	0	0	1	78	1
N size 2	14	39	57	13	48	13	13	16	81	34
N size 3	10	61	46	13	50	10	10	32	98	39
N size 4-12	109	222	163	91	371	89	91	264	386	219
N size13-30	112	51	40	100	51	129	127	83	33	87
N size 31+	18	8	26	18	0	11	12	5	4	7
Mean Modularity	0.71	0.62	0.63	0.71	0.71	0.73	0.73	0.69	0.5	0.73

Abbreviations: GDM – group detection method; N – number; Avg. com. size – average community size; Min. com. size – the size of the smallest community; Max. com. size – the size of the biggest community

As expected, communities found with ten GDMs differ (Fig S16 and S17 illustrate different partitions of the friendship network for two schools).

For our data on friendship networks of 22 schools, CP[2] provides the highest number of communities that are on average the smallest, but it also gives the highest number of communities that consist of only one person. The GDMs that result in a smaller number of communities that are consequently bigger on average are FG, LE, and LO. They also have no one-member communities, while EB and CP result in many such communities. LE, LO and WT have the highest mean of modularity[3] scores over 22 schools (0.73), suggesting that, on our dataset, they provide communities that are more connected within and less between. The communities with more than 30 members are found with all GDMs except IM. Given the rationale of the SBM algorithm is based on regular equivalence[4], it is not surprising that SBM has the lowest average modularity (0.5), because the group members

[2] Even though CP method gives more than one community membership for some students, we employed the approach used by Evans et al. (2016) according to which if a student belonged to more than one community, they were assigned to the community in which they had more ties.

[3] Modularity measures the strength of partition of a network into communities. A high modularity score means that a network has dense connections between the people within communities and sparse connections between people that are different communities.

[4] Structural equivalence identifies actors that have the same ties to exactly the same others in a network, while regular equivalence identifies actors that have identical ties to equivalent, but not necessarily identical, others (Hawe et al., 2004).

were not required to be connected but rather to have similar positions in the network. However, another blockmodeling method – BIA based on structural equivalence – has relatively high average modularity (0.71). Relatively low modularity for CP is consistent with detecting many communities with just one member who does not belong to any clique. But, since they are not isolates, they will have ties with others, which will decrease the modularity score because their ties are considered as being between communities.

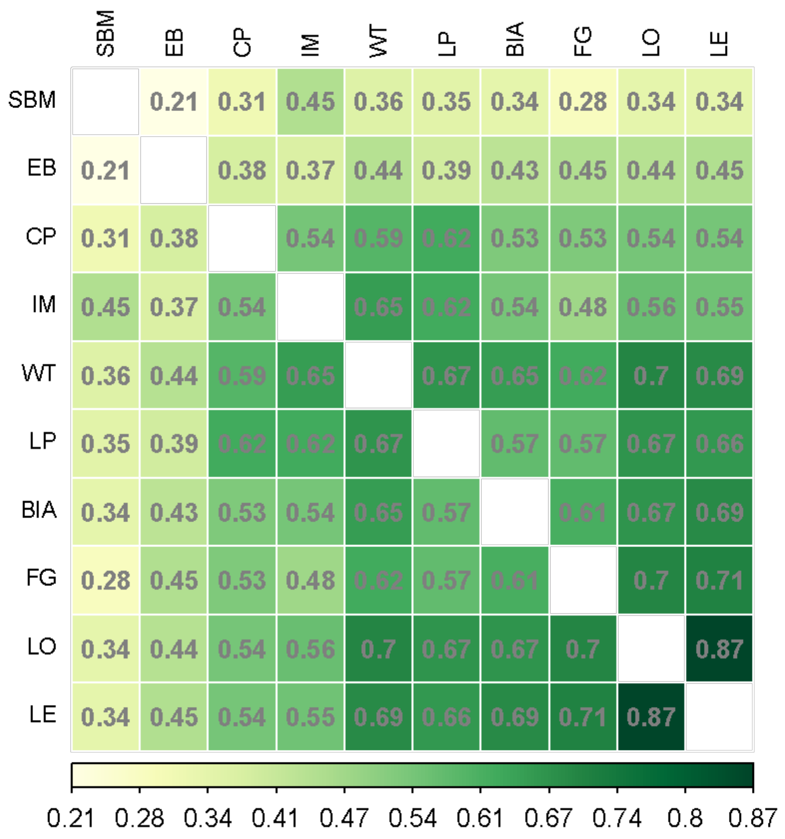

Fig S18. Average similarity based on adjusted Rand measure of 22 schools between ten GDMs (ordering by hierarchical clustering, method "average")

Fig S18 shows adjusted Rand (AR) indices for each pair of GDMs. AR can range from 0 (no overlap) to 1 (completely the same partition). The ordering of GDMs in the plot was done by hierarchical clustering (method average). SBM has the smallest overlap, followed by EB and CP, while LE and LO have the highest overlap with other methods (the highest being between the two).

Tables S15 - S17 further describe percentage of students in different types of communities for each GDM, the percentage of communities of each type for additional nine GDM, and their community properties.

Table S15. GDMs and percentage of all students in female, male, and mixed communities found in 22 schools

GDM	BIA	CP	EB	FG	IM	LE	LO	LP	SBM	WT
N Com.	300	895	546	235	525	252	253	401	680	387
N F com	100	281	213	77	215	86	83	167	285	156
N M com	93	317	174	66	213	75	77	149	275	148
% F com	33.33	31.4	39.01	32.77	40.95	34.13	32.81	41.65	41.91	40.31
%students in F com	26.51	33.36	24.52	23.49	36.94	25.71	25.65	34.89	36	33.13
%students in M com	25.3	32.54	20.82	20.96	38.78	26.97	27.35	33.41	35.14	32.42
%students in Mix com	48.2	34.09	54.65	55.55	24.27	47.33	47	31.71	28.86	34.45

Abbreviations: GDM – group detection method; N – number; Com. – community; F – female; M – male; Mix com – mixed communities

Table S16. Types of communities regarding gender (male, female, or mixed) found with nine GDMs

GDM	*Gender composition*	*N*	*%*
BIA	male	93	32.4
	mixed	94	32.8
	female	100	34.8
CP	male	317	47.5
	mixed	69	10.3
	female	281	42.1
EB	male	174	37.4
	mixed	78	16.8
	female	213	45.8
FG	male	66	28.1
	mixed	92	39.1
	female	77	32.8
IM	male	213	40.8
	mixed	94	18
	female	215	41.2
LE	male	75	29.8
	mixed	91	36.1
	female	86	34.1
LO	male	77	30.4
	mixed	93	36.8
	female	83	32.8

LP	male	149	37.2
	mixed	85	21.2
	female	167	41.6
SBM	male	275	41.3
	mixed	106	15.9
	female	285	42.8
WT	male	148	38.2
	mixed	83	21.4
	female	156	40.3

Table S17. Descriptives of five continuous community properties for additional nine GDMs

GDM	Properties	N	Mean	Median	SD	Min.	Max.	Range	Skew.	Kurtosis
BIA	Community size	300	12.16	11	9.59	1	62	61	1.24	2.46
	ROTC	300	0.42	0.31	0.32	0	1	1	0.94	-0.51
	Transitivity	237	0.58	0.54	0.17	0	1	1	0.51	0.52
	Centralization	237	0.24	0.22	0.11	0	0.71	0.71	1.12	1.92
	Hierarchy	237	0.09	0.04	0.15	-0.1	1	1.1	3.73	16.81
CP	Community size	895	4.08	1	6.71	1	98	97	5.65	54.51
	ROTC	895	0.76	1	0.31	0	1	1	-0.8	-0.96
	Transitivity	342	0.73	0.74	0.21	0	1	1	-0.47	0.45
	Centralization	341	0.26	0.25	0.15	0	0.75	0.75	0.29	0.65
	Hierarchy	341	0.25	0.11	0.32	-0.1	1	1.1	1.37	0.58
EB	Community size	546	6.68	3	12.48	1	106	105	4.29	22.15
	ROTC	546	0.63	0.67	0.35	0	1	1	-0.25	-1.47
	Transitivity	275	0.7	0.74	0.28	0	1	1	-0.75	-0.05
	Centralization	268	0.22	0.2	0.17	0	1	1	1.14	2.08
	Hierarchy	268	0.15	0.04	0.24	-0.25	1	1.25	2.05	3.76
FG	Community size	235	15.53	13	12.3	2	81	79	1.93	5.33
	ROTC	235	0.23	0.24	0.13	0	0.62	0.62	0.16	-0.13
	Transitivity	222	0.54	0.53	0.21	0	1	1	-0.2	1.03
	Centralization	215	0.22	0.2	0.12	0	1	1	2.09	8.26
	Hierarchy	215	0.08	0.03	0.15	-0.25	1	1.25	3.3	13.46
IM	Community size	525	6.95	6	3.95	1	28	27	1.19	2.25
	ROTC	525	0.38	0.38	0.19	0	1	1	0.25	0.27
	Transitivity	472	0.61	0.62	0.26	0	1	1	-0.78	0.49
	Centralization	460	0.32	0.3	0.16	0	1	1	1.21	3.25
	Hierarchy	460	0.15	0.06	0.23	-0.25	1	1.25	2.17	4.57
LE	Community size	252	14.48	14	8.5	2	45	43	0.76	0.54

	ROTC	252	0.23	0.24	0.12	0	0.56	0.56	-0.15	-0.3
	Transitivity	239	0.55	0.53	0.19	0	1	1	0.02	1.49
	Centralization	234	0.21	0.2	0.1	0	0.75	0.75	1.48	4.28
	Hierarchy	234	0.08	0.03	0.14	-0.25	1	1.25	3.84	19.48
LO	Community size	253	14.42	13	8.29	2	41	39	0.68	0.17
	ROTC	253	0.23	0.25	0.12	0	0.55	0.55	-0.32	-0.48
	Transitivity	240	0.54	0.54	0.18	0	1	1	-0.1	1.83
	Centralization	235	0.22	0.2	0.1	0	0.75	0.75	1.52	4.23
	Hierarchy	235	0.08	0.03	0.14	-0.25	1	1.25	3.95	19.73
LP	Community size	401	9.1	7	6.08	1	40	39	1.7	4.14
	ROTC	401	0.34	0.35	0.17	0	1	1	0.06	0.27
	Transitivity	384	0.62	0.6	0.24	0	1	1	-0.44	0.37
	Centralization	374	0.27	0.26	0.13	0	1	1	0.9	3.23
	Hierarchy	374	0.18	0.08	0.25	-0.25	1	1.25	1.87	2.84
SBM	Community size	680	5.37	4	4.64	1	43	42	3.36	18.61
	ROTC	680	0.67	0.68	0.27	0	1	1	-0.3	-1.11
	Transitivity	442	0.73	0.79	0.29	0	1	1	-1.1	0.58
	Centralization	446	0.24	0.23	0.18	0	1	1	0.99	2.11
	Hierarchy	446	0.11	0.02	0.2	-0.25	1	1.25	2.71	8.13

Abbreviations: GDM – community detection method; N – non-missing data; SD – standard deviation; Skew. – skewness.

Some communities have missing values for properties (transitivity, centralization, and hierarchy) that require certain number of members or ties to be calculated. Furthermore, in multi-level models, if some communities were populated with only non-responders, they could not have been used in the analysis. Therefore, there was a difference in total *N* at level 1 of analysis between model 1 and model 3, as shown in Table S18 for each GDM.

Table S18. Difference in sample size between Model 1 and 3 for all GDMs

GDM	*N in Model 1*	*N in Model 3*	*N dropped*	*% dropped*
BIA	3148	3076	72	2.29
CP	3148	2796	352	11.18
EB	3148	2917	231	7.34
FG	3148	3119	29	0.92
IM	3148	3054	94	2.99
LE	3148	3123	25	0.79
LO	3148	3123	25	0.79
LP	3148	3111	37	1.18
SBM	3148	2630	518	16.45
WT	3148	3079	69	2.19

6.1. Spearman correlations of the two outcomes and each community property

In the figure below, Spearman's correlations coefficients of the two outcomes at the individual level and each of six community properties are shown. Due to descriptive purpose of the analysis, *p*-values are not provided (*N* varied from 3079 - 3148). Note that community gender composition was converted to numerical variable, where 0 was assigned to mixed communities, 1 to female, and -1 to male communities.

Substance Use	Mental wellbeing
Community size	
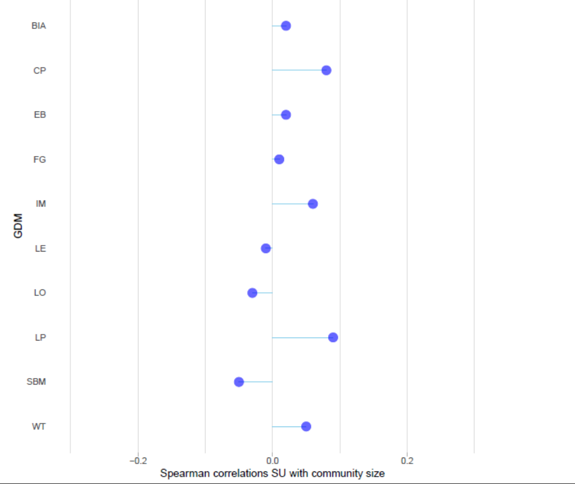	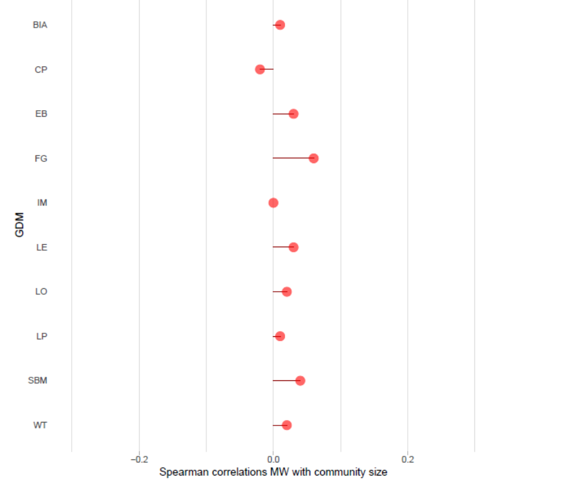
Community gender composition	
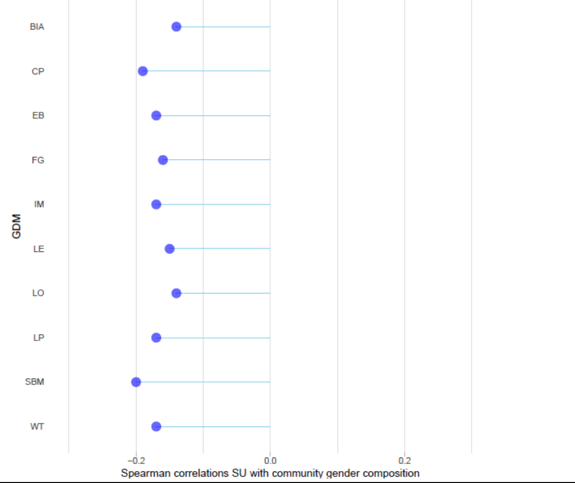	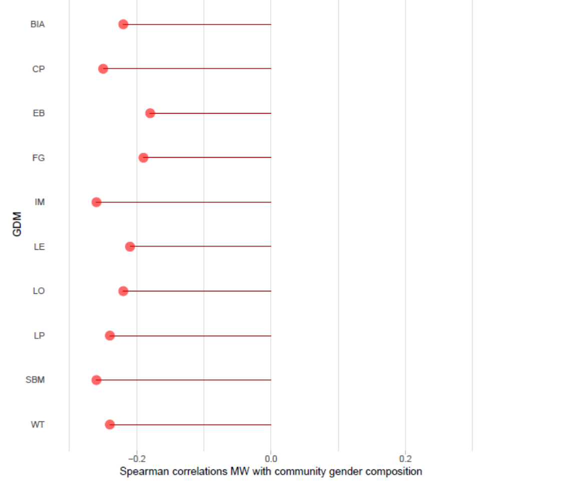
Ratio of ties outside community	

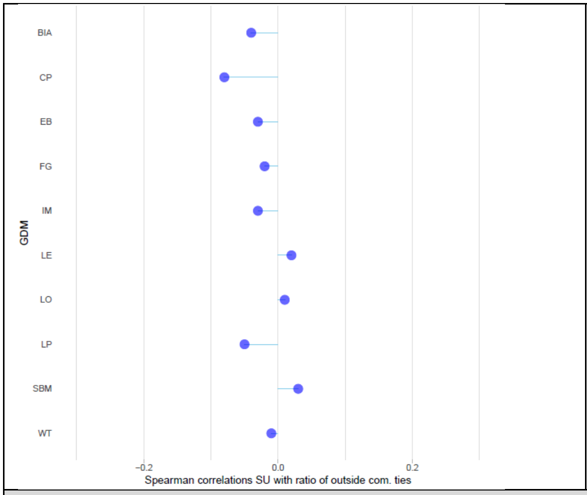

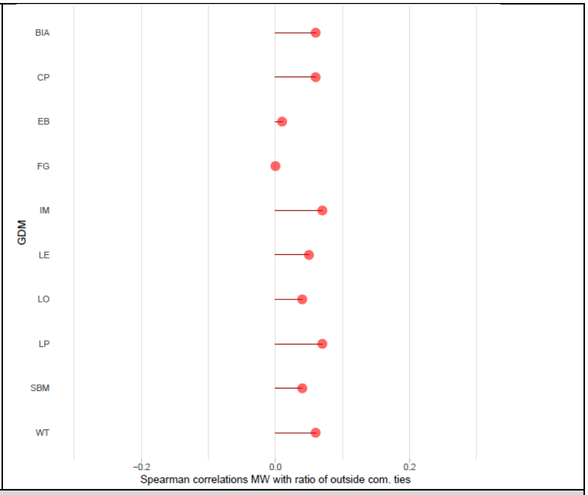

Transitivity

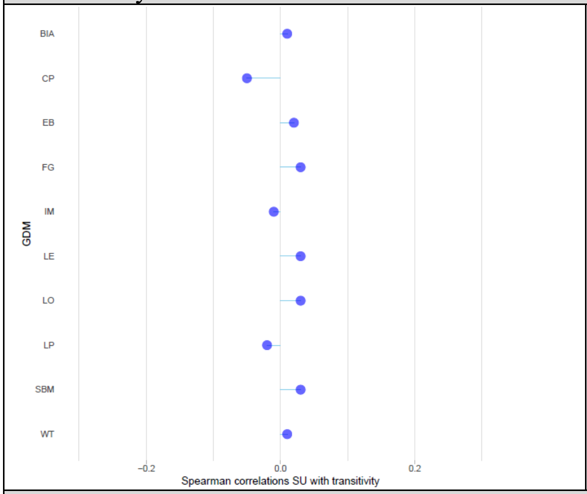

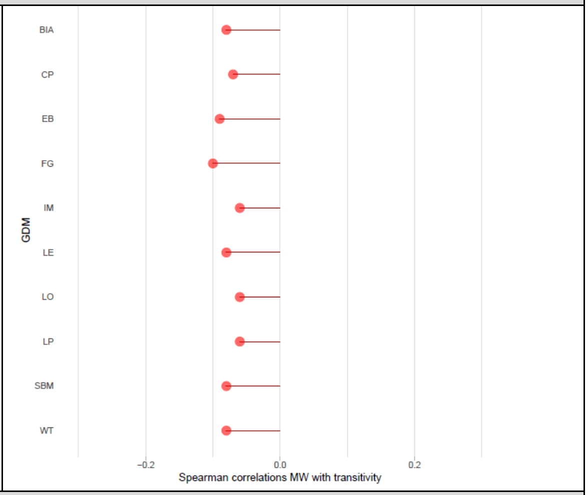

Centralization

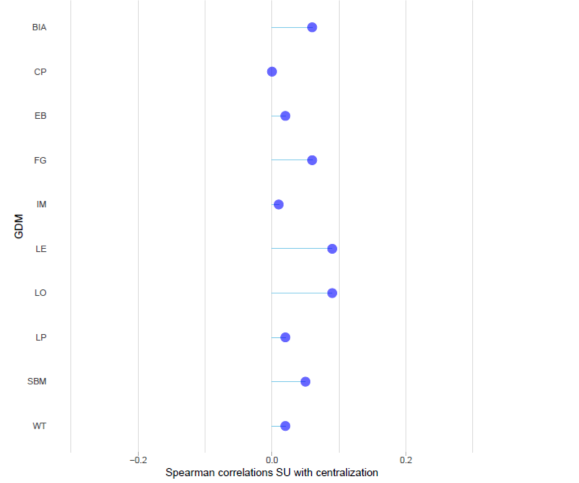

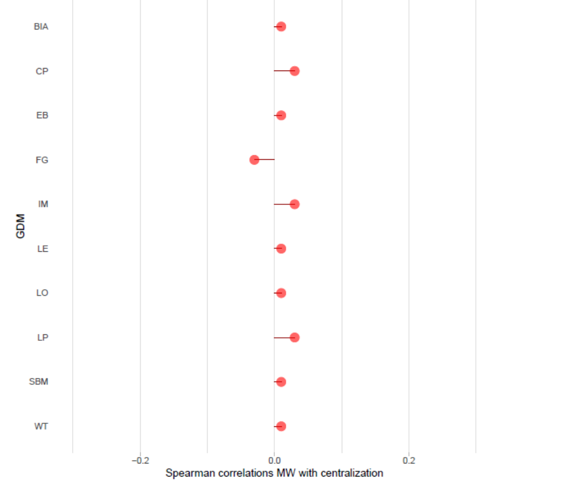

Hierarchy

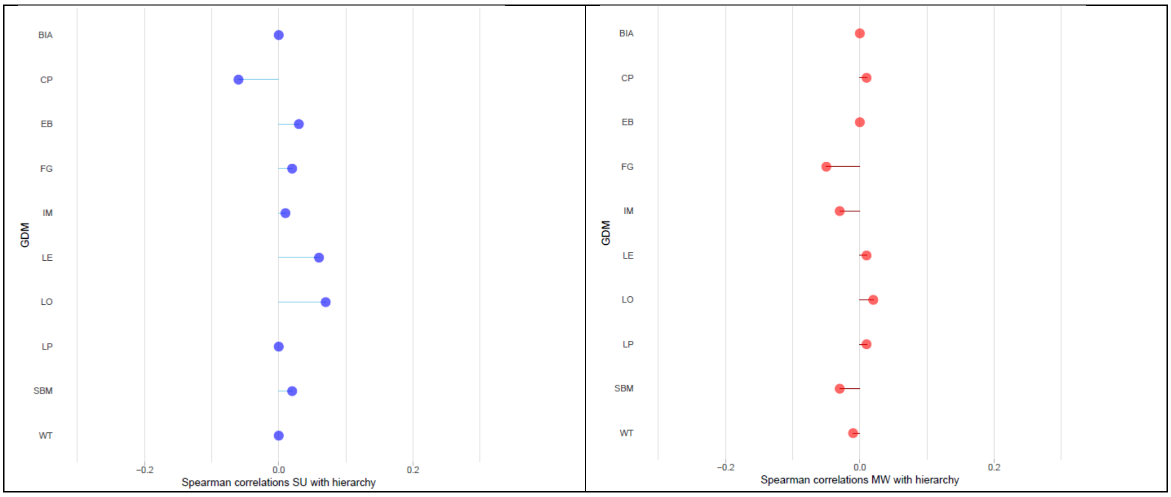

Fig S19. Spearman's correlation coefficients for associations between community properties and two outcomes across 10 GDMs

6.2. Bivariate relationship between six community properties and two health outcomes, controlling for community membership (as random effect) for 10 GDMs

We run series of multilevel models with peer group membership as random effect and one community property, for each community property and each GDM. The results (not including intercept parameter) are presented in the table below (S19).

Table S19. Bivariate relationship between six community properties and two health outcomes, controlling for community membership (as random effect) for 10 GDMs

		Substance use			*Mental wellbeing*		
GDM	*variable*	*Estimate*	*SE*	*p*	*Estimate*	*SE*	*p*
BIA	Community size	0.02	0.045	0.601	0.02	0.035	0.486
	Gender comp.- male	0.37	0.096	<0.001	0.59	0.07	<0.001
	Gender comp.- mixed	0.07	0.09	0.424	0.26	0.064	<0.001
	ROTC	-0.03	0.162	0.837	0.05	0.143	0.747
	Transitivity	-0.02	0.245	0.923	-0.45	0.194	0.023
	Centralization	0.06	0.383	0.882	0.2	0.308	0.528
	Hierarchy	-0.6	0.315	0.055	-0.05	0.269	0.862
	Variable	*Estimate*	*SE*	*p*	*Estimate*	*SE*	*p*
CP	Community size	0.12	0.067	0.077	-0.04	0.047	0.442
	Gender comp.- male	0.42	0.068	<0.001	0.63	0.052	<0.001

	Gender comp.- mixed	0.18	0.094	0.055	0.21	0.063	0.001
	ROTC	-0.11	0.103	0.27	0.24	0.087	0.006
	Transitivity	-0.16	0.187	0.386	-0.23	0.148	0.121
	Centralization	-0.03	0.271	0.921	0.3	0.218	0.165
	Hierarchy	-0.32	0.127	0.011	0.16	0.106	0.129
	Variable	*Estimate*	*SE*	*p*	*Estimate*	*SE*	*p*
EB	Community size	0.03	0.063	0.628	0.04	0.044	0.319
	Gender comp.- male	0.5	0.078	<0.001	0.51	0.063	<0.001
	Gender comp.- mixed	0.11	0.084	0.194	0.19	0.062	0.003
	ROTC	-0.13	0.116	0.28	0.03	0.1	0.758
	Transitivity	0.09	0.151	0.534	-0.25	0.117	0.033
	Centralization	-0.18	0.253	0.467	0.04	0.204	0.848
	Hierarchy	0.02	0.185	0.907	-0.1	0.153	0.505
	Variable	*Estimate*	*SE*	*p*	*Estimate*	*SE*	*p*
FG	Community size	0.02	0.054	0.645	0.06	0.04	0.129
	Gender comp.- male	0.42	0.103	<0.001	0.53	0.077	<0.001
	Gender comp.- mixed	0.12	0.091	0.195	0.23	0.067	0.001
	ROTC	-0.19	0.331	0.571	0.58	0.268	0.031
	Transitivity	0.03	0.222	0.894	-0.19	0.18	0.3
	Centralization	0.42	0.362	0.244	-0.23	0.292	0.437
	Hierarchy	-0.51	0.314	0.107	0.01	0.263	0.972
	Variable	*Estimate*	*SE*	*p*	*Estimate*	*SE*	*p*
IM	Community size	0.08	0.036	0.02	-0.01	0.029	0.655
	Gender comp.- male	0.36	0.068	<0.001	0.6	0.05	<0.001
	Gender comp.- mixed	0.1	0.084	0.213	0.13	0.059	0.03
	ROTC	-0.1	0.18	0.586	0.43	0.152	0.005
	Transitivity	-0.03	0.13	0.829	-0.17	0.11	0.117
	Centralization	-0.2	0.207	0.337	0.3	0.172	0.079
	Hierarchy	-0.25	0.15	0.092	0.03	0.128	0.834
	Variable	*Estimate*	*SE*	*p*	*Estimate*	*SE*	*p*
LE	Community size	0	0.041	0.927	0.04	0.033	0.234
	Gender comp.- male	0.39	0.096	<0.001	0.53	0.071	<0.001
	Gender comp.- mixed	0.11	0.089	0.223	0.17	0.065	0.008
	ROTC	0.2	0.342	0.568	0.71	0.278	0.012
	Transitivity	0.11	0.234	0.651	-0.26	0.192	0.183
	Centralization	0.92	0.408	0.025	-0.33	0.335	0.325
	Hierarchy	-0.36	0.337	0.287	-0.12	0.286	0.669
	Variable	*Estimate*	*SE*	*p*	*Estimate*	*SE*	*p*

LO	Community size	-0.02	0.04	0.666	0.02	0.031	0.455
	Gender comp.- male	0.36	0.096	<0.001	0.55	0.069	<0.001
	Gender comp.- mixed	0.12	0.089	0.171	0.16	0.063	0.01
	ROTC	0.03	0.341	0.932	0.74	0.278	0.008
	Transitivity	0.28	0.241	0.251	-0.15	0.199	0.452
	Centralization	0.66	0.405	0.105	-0.15	0.33	0.656
	Hierarchy	-0.05	0.326	0.875	-0.09	0.277	0.755
	Variable	*Estimate*	*SE*	*p*	*Estimate*	*SE*	*p*
LP	Community size	0.1	0.043	0.025	0	0.033	0.987
	Gender comp.- male	0.36	0.077	<0.001	0.59	0.056	<0.001
	Gender comp.- mixed	0.12	0.086	0.16	0.2	0.06	0.001
	ROTC	-0.31	0.218	0.161	0.63	0.179	<0.001
	Transitivity	0.02	0.158	0.891	-0.18	0.131	0.159
	Centralization	0.02	0.287	0.95	0.28	0.235	0.234
	Hierarchy	-0.11	0.152	0.462	0.02	0.129	0.848
	Variable	*Estimate*	*SE*	*p*	*Estimate*	*SE*	*p*
SBM	Community size	-0.08	0.044	0.075	0.03	0.036	0.401
	Gender comp.- male	0.45	0.057	<0.001	0.57	0.047	<0.001
	Gender comp.- mixed	-0.01	0.072	0.937	0.26	0.057	<0.001
	ROTC	0.16	0.103	0.121	0.15	0.089	0.087
	Transitivity	0.09	0.114	0.434	-0.25	0.091	0.008
	Centralization	0.06	0.186	0.737	0.17	0.156	0.286
	Hierarchy	-0.17	0.171	0.323	0	0.147	0.995
	Variable	*Estimate*	*SE*	*p*	*Estimate*	*SE*	*p*
WT	Community size	0.05	0.046	0.269	0.01	0.035	0.843
	Gender comp.- male	0.35	0.082	<0.001	0.6	0.06	<0.001
	Gender comp.- mixed	0.05	0.091	0.618	0.2	0.064	0.002
	ROTC	-0.11	0.258	0.679	0.59	0.216	0.007
	Transitivity	0.31	0.157	0.047	-0.31	0.132	0.019
	Centralization	-0.25	0.262	0.336	0.01	0.216	0.954
	Hierarchy	-0.02	0.174	0.925	-0.1	0.147	0.479

GDM – group detection method; SE – standard error; ROTC – ratio of ties outside community
Shaded cell – *p* value =<0.10

When individual covariates and other community properties were not included in the model, we can notice that overall, there was a higher number of significant effects for Mental Wellbeing than Substance Use. The highest number of significant community properties was uncovered by CP, followed by IM. The smallest number of significant

community properties was found for EB. Despite being blockmodeling-based techniques, both SBM and BIA have significant community effects.

6.3. Checking the existence of non-linear relationship between community properties and two health outcomes

To examine the potential non-linear effects of community properties on the two outcomes, we constructed separate models for each outcome. These models included random effects of communities and a community property along with its quadratic term. This analysis was performed for all community properties, except for Community gender composition, which was treated as a nominal variable in our study.

Table S20. Linear and quadratic effects of community properties, controlling for random effect of community membership.

	Dependent variable	*Substance use*			*Mental wellbeing*		
GDM	*Parameter*	*Estimate*	*SE*	*p*	*Estimate*	*SE*	*p*
BIA	Community size	0.02	0.05	0.59	0.03	0.04	0.45
	Community size quadratic term	-0.01	0.03	0.81	-0.01	0.02	0.71
	ROTC	-0.7	0.58	0.23	1.35	0.46	**<0.001**
	ROTC quadratic term	0.64	0.53	0.23	-1.3	0.44	<0.001
	Transitivity	-0.45	1.2	0.71	-2.27	0.95	**0.02**
	Transitivity quadratic term	0.35	0.96	0.72	1.49	0.76	0.05
	Centralization	1.67	1.31	0.2	-0.61	1.05	0.56
	Centralization quadratic term	-2.74	2.13	0.2	1.4	1.74	0.42
	Hierarchy	-0.46	0.71	0.52	-0.47	0.59	0.42
	Hierarchy quadratic term	-0.22	0.96	0.82	0.68	0.83	0.41
	Dependent variable	*Substance use*			*Mental wellbeing*		
	Parameter	*Estimate*	*SE*	*p*	*Estimate*	*SE*	*p*
CP	Community size	0.15	0.08	**0.07**	-0.08	0.06	0.2
	Community size quadratic term	-0.02	0.04	0.54	0.03	0.03	0.27
	ROTC	-0.28	0.55	0.61	0.86	0.43	**0.05**
	ROTC quadratic term	0.14	0.44	0.76	-0.52	0.35	0.14
	Transitivity	-0.79	0.89	0.38	-1.34	0.75	**0.07**
	Transitivity quadratic term	0.45	0.63	0.47	0.81	0.53	0.13
	Centralization	-0.63	0.71	0.38	-0.24	0.59	0.68

	Centralization quadratic term	1.09	1.21	0.37	1	1	0.32
	Hierarchy	-0.67	0.44	0.12	0.49	0.35	0.16
	Hierarchy quadratic term	0.38	0.45	0.4	-0.36	0.37	0.33
	Dependent variable	*Substance use*			*Mental wellbeing*		
	Parameter	*Estimate*	*SE*	*p*	*Estimate*	*SE*	*p*
EB	Community size	0.03	0.07	0.68	0.07	0.05	0.14
	Community size quadratic term	0	0.06	0.94	-0.05	0.04	0.22
	ROTC	0.21	0.47	0.65	0.36	0.39	0.35
	ROTC quadratic term	-0.31	0.42	0.46	-0.31	0.35	0.38
	Transitivity	0.08	0.6	0.89	0.81	0.49	**0.1**
	Transitivity quadratic term	0.01	0.47	0.99	-0.84	0.38	0.03
	Centralization	0.29	0.61	0.63	0.28	0.49	0.56
	Centralization quadratic term	-0.78	0.91	0.39	-0.41	0.75	0.58
	Hierarchy	0.78	0.5	0.12	-0.14	0.4	0.74
	Hierarchy quadratic term	-1.01	0.62	0.1	0.05	0.51	0.93
	Dependent variable	*Substance use*			*Mental wellbeing*		
	Parameter	*Estimate*	*SE*	*p*	*Estimate*	*SE*	*p*
FG	Community size	0.04	0.06	0.54	0.06	0.04	0.17
	Community size quadratic term	-0.02	0.04	0.6	0	0.03	0.97
	ROTC	0.29	0.97	0.77	0.86	0.81	0.29
	ROTC quadratic term	-0.96	1.83	0.6	-0.55	1.51	0.72
	Transitivity	0.6	0.75	0.42	0.57	0.65	0.38
	Transitivity quadratic term	-0.53	0.66	0.42	-0.68	0.57	0.23
	Centralization	0.62	0.89	0.48	0.37	0.7	0.6
	Centralization quadratic term	-0.31	1.24	0.8	-0.96	1.04	0.35
	Hierarchy	0.52	0.68	0.45	-0.06	0.56	0.92
	Hierarchy quadratic term	-1.67	0.99	0.09	0.11	0.86	0.89
	Dependent variable	*Substance use*			*Mental wellbeing*		
	Parameter	*Estimate*	*SE*	*p*	*Estimate*	*SE*	*p*
IM	Community size	0.1	0.04	**0.01**	-0.03	0.03	0.33
	Community size quadratic term	-0.04	0.02	0.1	0.03	0.02	0.07
	ROTC	-0.3	0.58	0.61	1.03	0.5	**0.04**
	ROTC quadratic term	0.26	0.72	0.72	-0.79	0.62	0.21
	Transitivity	0.21	0.4	0.6	0.21	0.34	0.54
	Transitivity quadratic term	-0.22	0.36	0.53	-0.35	0.3	0.24
	Centralization	-0.22	0.57	0.7	0.27	0.48	0.57
	Centralization quadratic term	0.03	0.66	0.97	0.04	0.55	0.94
	Hierarchy	-0.22	0.39	0.58	0.07	0.32	0.82

	Hierarchy quadratic term	-0.05	0.47	0.91	-0.06	0.39	0.87
	Dependent variable	*Substance use*			*Mental wellbeing*		
	Parameter	*Estimate*	*SE*	*p*	*Estimate*	*SE*	*p*
LE	Community size	0	0.04	0.92	0.04	0.03	0.22
	Community size quadratic term	0	0.03	0.95	-0.04	0.02	0.11
	ROTC	0.19	1.06	0.86	1.27	0.89	0.16
	ROTC quadratic term	0.01	2.12	1	-1.16	1.75	0.51
	Transitivity	0.55	0.88	0.53	0.58	0.76	0.45
	Transitivity quadratic term	-0.39	0.74	0.6	-0.73	0.64	0.26
	Centralization	1.6	1.22	0.19	1.43	1.01	0.16
	Centralization quadratic term	-1.24	2.1	0.56	-3.3	1.78	0.06
	Hierarchy	0.91	0.69	0.19	0.12	0.57	0.83
	Hierarchy quadratic term	-2.07	0.98	0.04	-0.42	0.86	0.62
	Dependent variable	*Substance use*			*Mental wellbeing*		
	Parameter	*Estimate*	*SE*	*p*	*Estimate*	*SE*	*p*
LO	Community size	-0.02	0.04	0.64	0.02	0.03	0.46
	Community size quadratic term	-0.01	0.03	0.79	-0.01	0.03	0.82
	ROTC	0.13	1.12	0.91	1.31	0.95	0.17
	ROTC quadratic term	-0.22	2.32	0.92	-1.2	1.93	0.53
	Transitivity	-0.47	0.88	0.6	1.09	0.77	0.16
	Transitivity quadratic term	0.67	0.76	0.38	-1.1	0.66	0.1
	Centralization	1.37	1.25	0.28	1.45	1.03	0.16
	Centralization quadratic term	-1.28	2.14	0.55	-2.96	1.81	0.1
	Hierarchy	1.32	0.69	**0.06**	0.22	0.57	0.71
	Hierarchy quadratic term	-2.14	0.96	0.03	-0.5	0.83	0.55
	Dependent variable	*Substance use*			*Mental wellbeing*		
	Parameter	*Estimate*	*SE*	*p*	*Estimate*	*SE*	*p*
LP	Community size	0.1	0.05	**0.03**	-0.01	0.04	0.82
	Community size quadratic term	-0.01	0.03	0.74	0.01	0.02	0.61
	ROTC	0.5	0.68	0.46	0.84	0.58	0.15
	ROTC quadratic term	-1.15	0.92	0.21	-0.29	0.79	0.71
	Transitivity	0.28	0.57	0.62	0.61	0.49	0.22
	Transitivity quadratic term	-0.22	0.46	0.63	-0.67	0.4	0.1
	Centralization	-0.7	0.76	0.36	1.42	0.63	0.03
	Centralization quadratic term	1.11	1.09	0.31	-1.79	0.92	0.05
	Hierarchy	-0.17	0.44	0.7	0.58	0.36	**0.1**
	Hierarchy quadratic term	0.07	0.51	0.89	-0.71	0.43	0.09
	Dependent variable	*Substance use*			*Mental wellbeing*		

	Parameter	*Estimate*	*SE*	*p*	*Estimate*	*SE*	*p*
SBM	Community size	-0.12	0.05	**0.03**	0.08	0.05	**0.09**
	Community size quadratic term	0.03	0.03	0.21	-0.04	0.02	0.09
	ROTC	-0.35	0.51	0.49	0.91	0.43	**0.04**
	ROTC quadratic term	0.42	0.41	0.31	-0.63	0.35	0.07
	Transitivity	-0.35	0.39	0.38	-0.15	0.31	0.64
	Transitivity quadratic term	0.38	0.33	0.25	-0.08	0.26	0.75
	Centralization	-0.11	0.43	0.8	-0.18	0.36	0.62
	Centralization quadratic term	0.27	0.6	0.65	0.54	0.51	0.29
	Hierarchy	0.2	0.4	0.62	0.04	0.33	0.9
	Hierarchy quadratic term	-0.5	0.5	0.31	-0.06	0.42	0.89
	Dependent variable	*Substance use*			*Mental wellbeing*		
	Parameter	*Estimate*	*SE*	*p*	*Estimate*	*SE*	*p*
WT	Community size	0.05	0.05	0.27	0	0.04	0.97
	Community size quadratic term	0	0.04	0.91	0.02	0.03	0.49
	ROTC	0.6	0.73	0.41	1.08	0.62	**0.09**
	ROTC quadratic term	-1.14	1.1	0.3	-0.8	0.97	0.41
	Transitivity	0.35	0.49	0.47	0.04	0.42	0.93
	Transitivity quadratic term	-0.03	0.43	0.94	-0.32	0.37	0.38
	Centralization	0.04	0.68	0.95	0.91	0.55	**0.1**
	Centralization quadratic term	-0.4	0.85	0.64	-1.25	0.71	0.08
	Hierarchy	-0.52	0.46	0.25	0.39	0.38	0.3
	Hierarchy quadratic term	0.66	0.55	0.23	-0.66	0.46	0.15

GDM – group detection method
SE – standard error; ROTC – ratio of ties outside community
Shaded cell – quadratic term with *p* value =<0.10
Bold font – non-quadratic term with *p* value =<0.10

Table S20 shows evidence of non-linear associations between certain community properties and the two outcomes across 10 GDMs (intercepts are not reported). Notably, non-linear effects appear to be more prevalent for Mental wellbeing than for Substance use and they seem to exist for at least one property in all GDMs, except for Clique Percolation.

6.4. Sensitivity of effects of community properties on two health outcomes to group detection method

Tables S21 and S22 summarise results of all GDMs for Model, for SU and MW, respectively. The Tables show for each community property and each GDM *p*-values after they are corrected for multi-testing (10 tests) by using false discovery rate (FDR) method (Benjamini & Yekutieli, 2001).

.

Table S21. Community property effects for Substance Use (Model 3) for 10 GDMs – after correction for multi-testing (FDR)

GDM		Size	Gender. comp. male	Gender. comp. mixed	ROTC	Transitivity	Centralization	Hierarchy
BIA	Est.	0.04	0.026	-0.077	-0.355	0.346	0.292	-0.613
	p	0.674	0.819	0.928	0.62	0.245	0.726	0.138
CP	Est.	0.134	0.18	-0.033	-0.345	0.399	0.071	-0.286
	p	0.402	0.173	0.928	0.62	0.186	0.838	**0.1**
EB	Est.	0.045	0.311	-0.011	-0.181	0.214	-0.057	-0.105
	p	0.674	**0.01**	0.928	0.702	0.307	0.838	0.689
FG	Est.	0.07	0.081	-0.031	-0.407	0.364	0.797	-0.769
	p	0.592	0.591	0.928	0.62	0.211	0.2	**0.1**
IM	Est.	0.022	0.067	-0.016	-0.138	0.126	-0.18	-0.177
	p	0.674	0.591	0.928	0.84	0.412	0.726	0.316
LE	Est.	0.098	0.073	-0.011	-0.013	0.593	1.512	-0.723
	p	0.253	0.591	0.928	0.967	**0.073**	**0.01**	**0.1**
LO	Est.	0.028	0.023	0.03	-0.16	0.6	0.746	-0.516
	p	0.674	0.819	0.928	0.901	**0.073**	0.31	0.222
LP	Est.	0.133	0.147	0.008	-0.257	0.458	0.231	-0.007
	p	0.18	0.236	0.928	0.62	**0.065**	0.726	0.964
SBM	Est.	0.017	0.243	-0.07	0.031	0.225	0.088	-0.234
	p	0.807	**0.045**	0.928	0.944	0.205	0.806	0.223
WT	Est.	0.128	0.16	-0.063	0.088	0.63	0.227	-0.056
	p	0.2	0.23	0.928	0.944	**0.01**	0.726	0.831

Abbreviations: Est. – estimate; *p* – *p*-value; Gender.comp. – gender composition of community
Reference group for Gender composition: female
In bold font: *p*-values =<0.10

Table S22. Community property effects for Mental Wellbeing (Model 3) for 10 GDMs – after correction for multi-testing (FDR)

GDM		Size	Gender. comp. male	Gender. comp. mixed	ROTC	Transitivity	Centralization	Hierarchy
BIA	Est.	-0.085	-0.058	-0.047	0.372	-0.328	-0.435	0.237
	p	0.225	0.845	0.611	0.78	0.3	0.472	0.93
CP	Est.	-0.038	-0.007	-0.046	0.235	-0.31	-0.124	0.222
	p	0.728	0.935	0.611	0.789	0.3	0.729	0.39
EB	Est.	-0.038	-0.155	-0.178	0.055	-0.33	-0.113	-0.084
	p	0.728	0.58	0.2	0.789	0.17	0.729	0.96
FG	Est.	-0.014	-0.067	-0.06	0.137	-0.16	-0.371	0.267
	p	0.765	0.845	0.544	0.789	0.534	0.472	0.93
IM	Est.	-0.016	0.018	-0.087	0.107	-0.05	-0.019	0.106
	p	0.728	0.935	0.488	0.789	0.689	0.915	0.93
LE	Est.	-0.06	-0.081	-0.113	0.069	-0.286	-0.708	0.016
	p	0.316	0.845	0.35	0.789	0.315	0.167	0.974
LO	Est.	-0.069	-0.058	-0.117	0.18	-0.134	-0.702	-0.201
	p	0.225	0.845	0.35	0.789	0.601	0.167	0.93
LP	Est.	-0.08	-0.063	-0.061	0.182	-0.212	-0.22	0.004
	p	0.225	0.845	0.544	0.789	0.312	0.58	0.974
SBM	Est.	0.023	-0.032	-0.086	0.063	-0.094	0.07	0.036
	p	0.728	0.935	0.502	0.789	0.534	0.729	0.974
WT	Est.	-0.133	0.014	-0.018	-0.141	-0.596	-0.585	0.033
	p	**0.04**	0.935	0.798	0.789	**<0.001**	0.14	0.974

Abbreviations: Est. – estimate; *p* – *p*-value; Gender.comp. – gender composition of community
Reference group for Gender composition: female
In bold font: *p*-values =< 0.10

Outcome: Substance Use *Outcome: Mental Wellbeing*

Com. Size

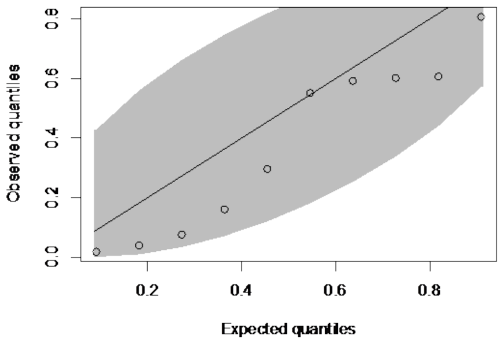

Com. Size

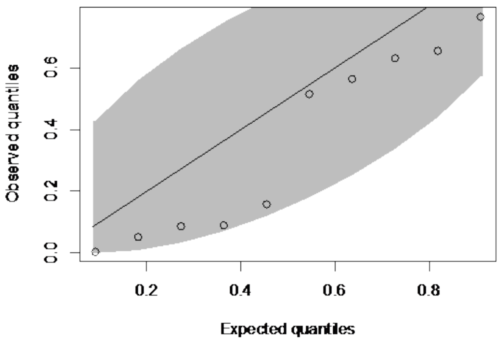

Gender.comp.male_female

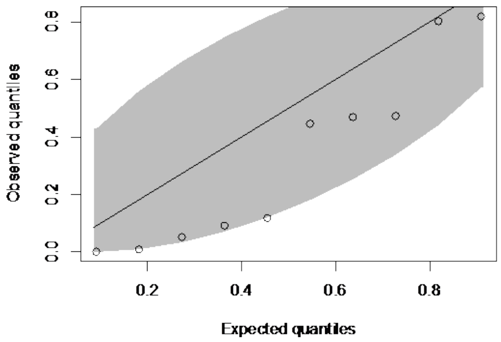

Gender.comp.male_female

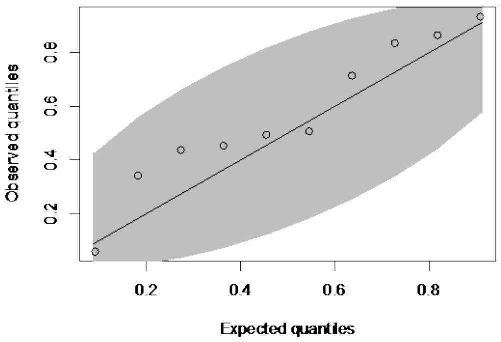

Gender.comp.mixed_female

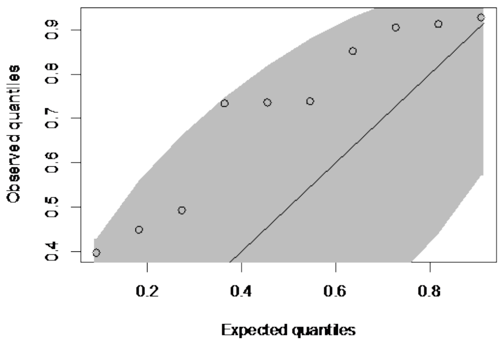

Gender.comp.mixed_female

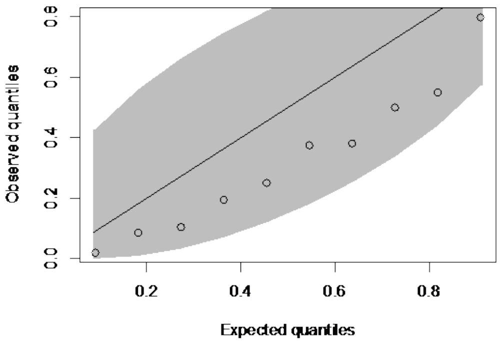

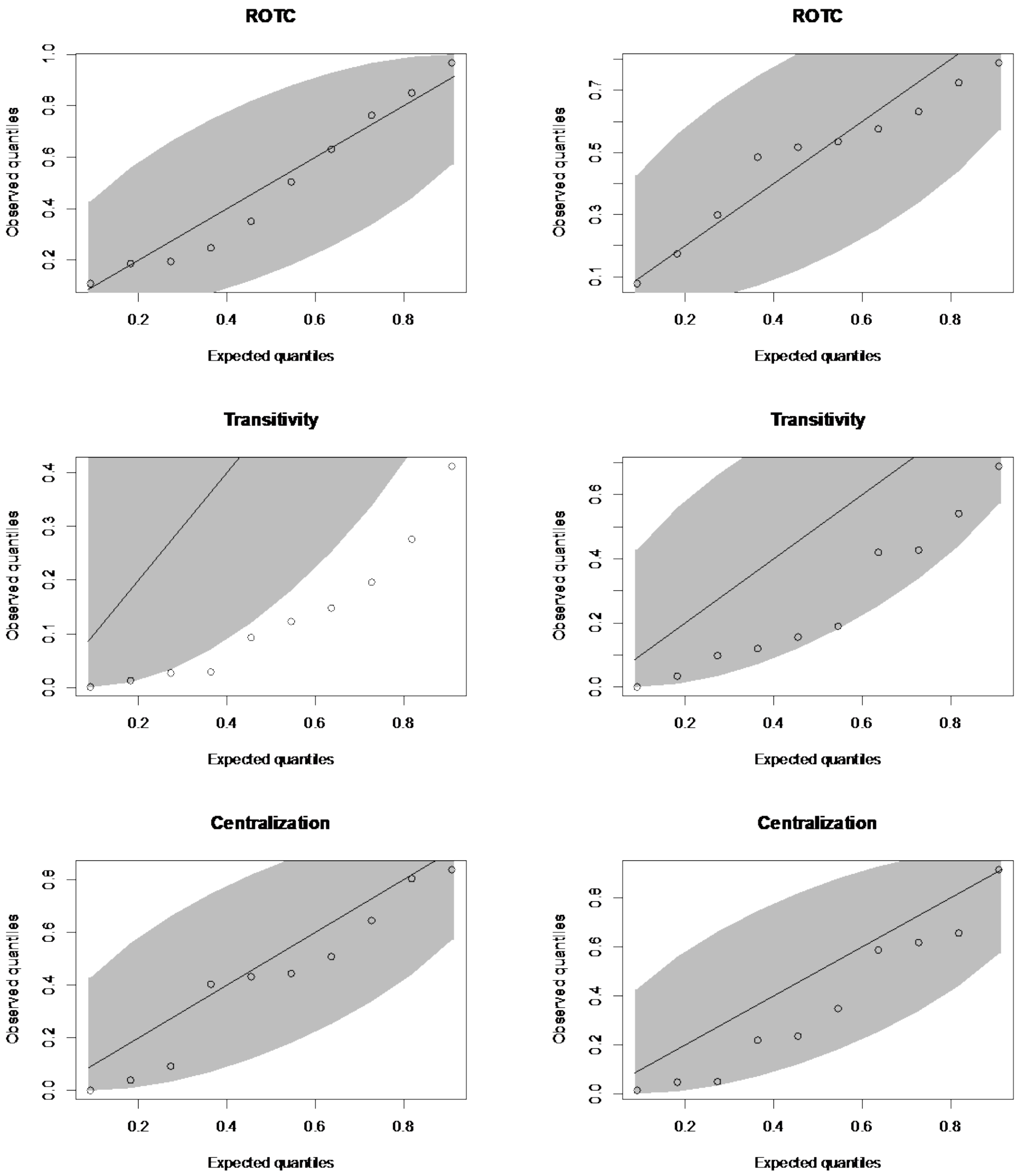
ROTC
ROTC
Transitivity
Transitivity
Centralization
Centralization
Observed quantiles
Expected quantiles
0.2
0.4
0.6
0.8

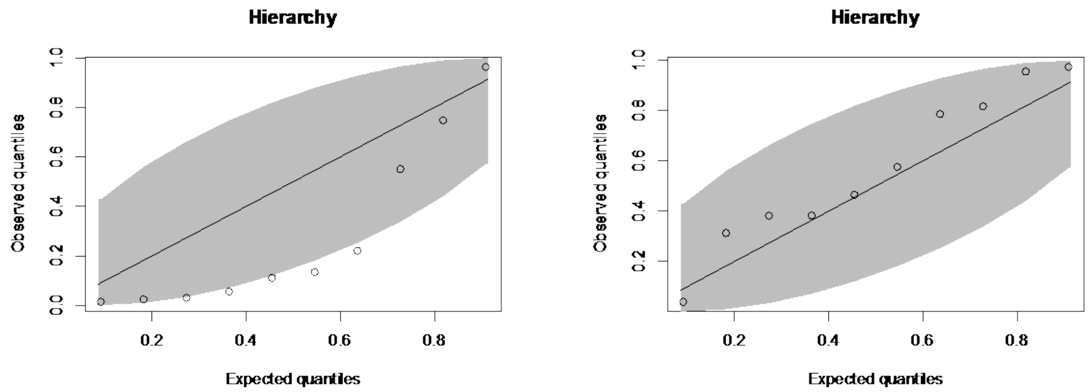

Fig S20. Q–Q plots for ten p–values resulted from each GDM for each community property for the two health outcomes.

Additionally, we examined the original *p*–values graphically using metap R package (Dewey, 2022). Q–Q plots in Fig S20 show ten p–values resulted from each GDM for each community property (Gender composition as dummy variable) for the two health outcomes. The line in each plot is the line of exact fit to the reference uniform distribution. The grey polygon area in each plot shows the simultaneous confidence region where points that do not belong to the uniform distribution lie outside the polygon. Fig S20 shows that for both outcomes and for most community properties ten p-values fell within the expected grey area of multiple testing null results, but some go beyond it. Looking at community property ROTC (both outcomes), there is no evidence of a trend towards association. However, there was evidence of a trend for transitivity and SU association. We can see that for most community properties there were one or more points near the edge of grey area, showing that for most community properties at least one GDM resulted in a p-value that when multitesting is considered still suggest the existence of a significant effect for a specific GDM.

7 Robustness checks

Due to the novelty of our findings, we ran several post-hoc robustness tests.

7.1 Raw scores and factor scores for Substance use and Mental wellbeing as outcome

Model 3 with raw composite score and factor scores as outcome variables (Walktrap) are shown in Table S23.

The results with factor scores and raw scores are overall similar to the results found for principal component scores. However, both scores show a higher clustering for SU and smaller clustering for MW then what is found for principal component scores. Community properties show similar effects for factor scores, but for raw scores no effects were significant for MW as the outcome. There is a tendency for difference in direction of estimates for the same community property effect for the two outcomes for both raw scores and factor scores. Raw scores are often used in research because they are simple, straightforward to interpret and less dependent on sample characteristics (DiStefano et al., 2009). The downside is that they can obscure results when the structure of correlations between variables is more complex.

Table S23. Dependent variable: Raw scores and factor scores for Substance use and Mental Wellbeing- results for Walktrap community detection method; Model 3

	Raw scores		*Factor scores*	
parameter	*Substance Use*	*Mental Wellbeing*	*Substance Use*	*Mental Wellbeing*
Level 1 covariates				
Gender (male)	-0.05 [-0.15, 0.06]	**0.59 [0.48, 0.71]**	0.07 [-0.03, 0.18]	**0.63 [0.52, 0.74]**
Age	**-0.08 [-0.14, -0.03]**	-0.04 [-0.1, 0.03]	-0.06 [-0.12, 0]	-0.01 [-0.07, 0.05]
Ethnicity (white)	**-0.51 [-0.63, -0.39]**	**-0.14 [-0.26, -0.02]**	**-0.4 [-0.53, -0.28]**	0 [-0.13, 0.12]
Family affluence (medium)	0.08 [-0.01, 0.16]	0.01 [-0.08, 0.1]	0.09 [0, 0.18]	-0.03 [-0.13, 0.06]
Family affluence (high)	0.02 [-0.07, 0.11]	0.01 [-0.08, 0.11]	0.06 [-0.03, 0.15]	-0.01 [-0.1, 0.09]
Parental control	-0.02 [-0.05, 0.01]	**-0.21 [-0.25, -0.18]**	**-0.07 [-0.11, -0.04]**	**-0.19 [-0.22, -0.16]**
Parental care	**0.18 [0.14, 0.21]**	**0.19 [0.16, 0.22]**	**0.2 [0.17, 0.23]**	**0.14 [0.11, 0.18]**
Level 2 covariates				
Community size	**0.17 [0.03, 0.31]**	-0.01 [-0.07, 0.06]	**0.18 [0.05, 0.31]**	**-0.1 [-0.18, -0.02]**
Gender comp.(male)	0.15 [-0.06, 0.35]	0.06 [-0.09, 0.2]	0.11 [-0.08, 0.31]	0.08 [-0.07, 0.23]
Gender comp.(mixed)	-0.05 [-0.25, 0.16]	-0.06 [-0.17, 0.05]	-0.06 [-0.25, 0.13]	-0.01 [-0.13, 0.11]
ROTC	0.06 [-0.58, 0.7]	-0.02 [-0.38, 0.33]	0.26 [-0.35, 0.86]	-0.22 [-0.61, 0.18]
Transitivity	**0.79 [0.36, 1.22]**	-0.04 [-0.3, 0.21]	**0.84 [0.43, 1.24]**	**-0.49 [-0.77, -0.22]**
Centralization	0.41 [-0.24, 1.06]	-0.27 [-0.65, 0.12]	0.35 [-0.27, 0.97]	**-0.47 [-0.88, -0.05]**
Hierarchy	-0.03 [-0.41, 0.35]	-0.04 [-0.28, 0.2]	-0.1 [-0.46, 0.26]	0.02 [-0.24, 0.29]
Num. obs.	3079	3079	3079	3079
N groups: Community	339	339	339	339
AIC	7691.67	7997.09	7740.05	8064.60
BIC	7794.22	8099.64	7842.60	8167.15
Log Likelihood	-3828.84	-3981.55	-3853.02	-4015.30
Var: Community (Intercept)	0.38	0.03	0.32	0.06
Var: Residual	0.57	0.74	0.59	0.74
ICCadj./ICCcond.	0.40/0.37	0.04/0.03	0.36/0.32	0.07/0.06
R^2mar./ R^2cond.	0.07/0.44	0.22/0.25	0.09/0.41	0.20/0.26

Abbreviations: Gender comp. – community gender composition; Num. obs. – Number of observations; AIC – Akaike information criterion; BIC – Bayesian information criterion; Var – variance; N groups – number of groups; ICCadj. – adjusted intraclass correlation coefficient; ICCcond. – conditional intraclass correlation coefficient; R^2mar. – marginal R^2; R^2cond. – conditional R^2; Age is dichotomized: 15 yrs = 0; 16 and 17 yrs = 1
Reference categories for factors: Gender – female; Ethnicity – non-white; Family affluence – low; Community gender comp. – female

7.2 Differences between schools with high and low response rate (all GDMs)

For checking the sensitivity of findings to missing attribute data (as a specific kind of robustness), we ran separate analysis on two subsamples made of 11 schools with lowest non-response rate and 11 schools with highest non-response rate (Model 1). High-responding schools are all schools that have less than 12.5% non-responders.

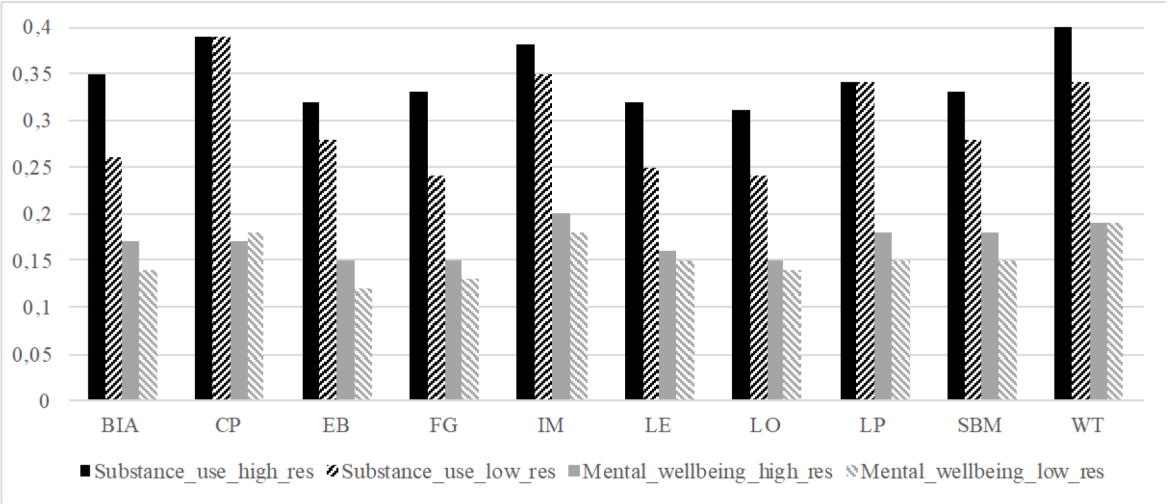

Legend:
Substance use high res – ICC on sample of schools with relatively less non-responders
Mental wellbeing high res – ICC on sample of schools with relatively less non-responders
Substance use low res – ICC on sample of schools with relatively more non-responders
Mental wellbeing low res – ICC on sample of schools with relatively more non-responders

Fig S21. Adjusted intraclass correlation coefficients (ICC) for Substance use and Mental wellbeing (Model 1) for each GDM on subsample of high responding schools (11 schools, N=1605) and subsample of low responding schools (11 schools, N=1543) and ICC for being a non-responder as dependent variable and communities as random effect (x-axis: community detection algorithm, y-axis: ICC values)

As shown in Fig S21, for ten GDMs, estimates of ICC for both outcomes are overall similar for schools with high response rate and schools with low response rate.

8 Two health outcomes and Walktrap communities

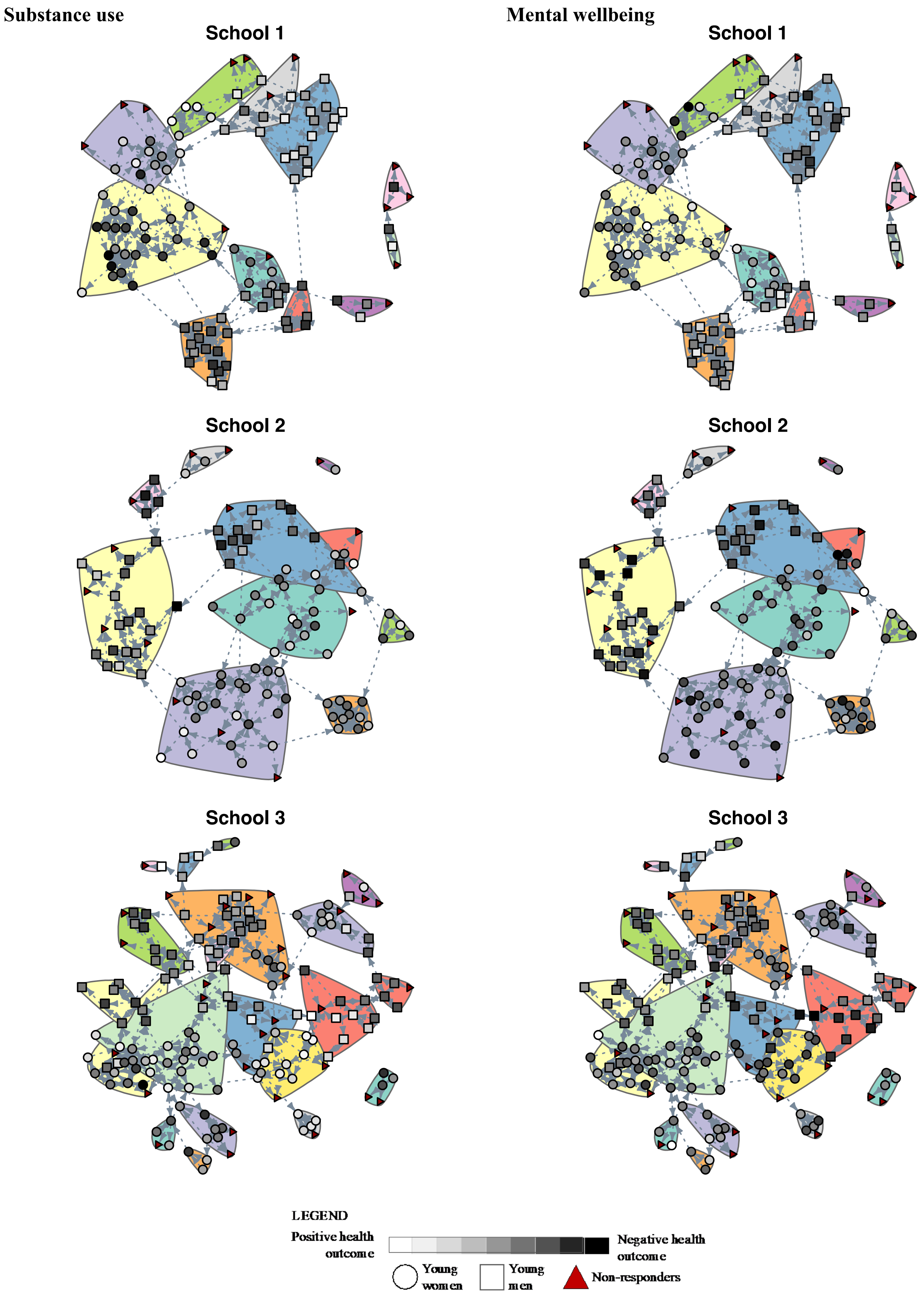

Substance use
School 1
Mental wellbeing
School 1
School 2
School 2
School 3
School 3
LEGEND
Positive health outcome
Negative health outcome
Young women
Young men
Non-responders

Substance use **Mental wellbeing**

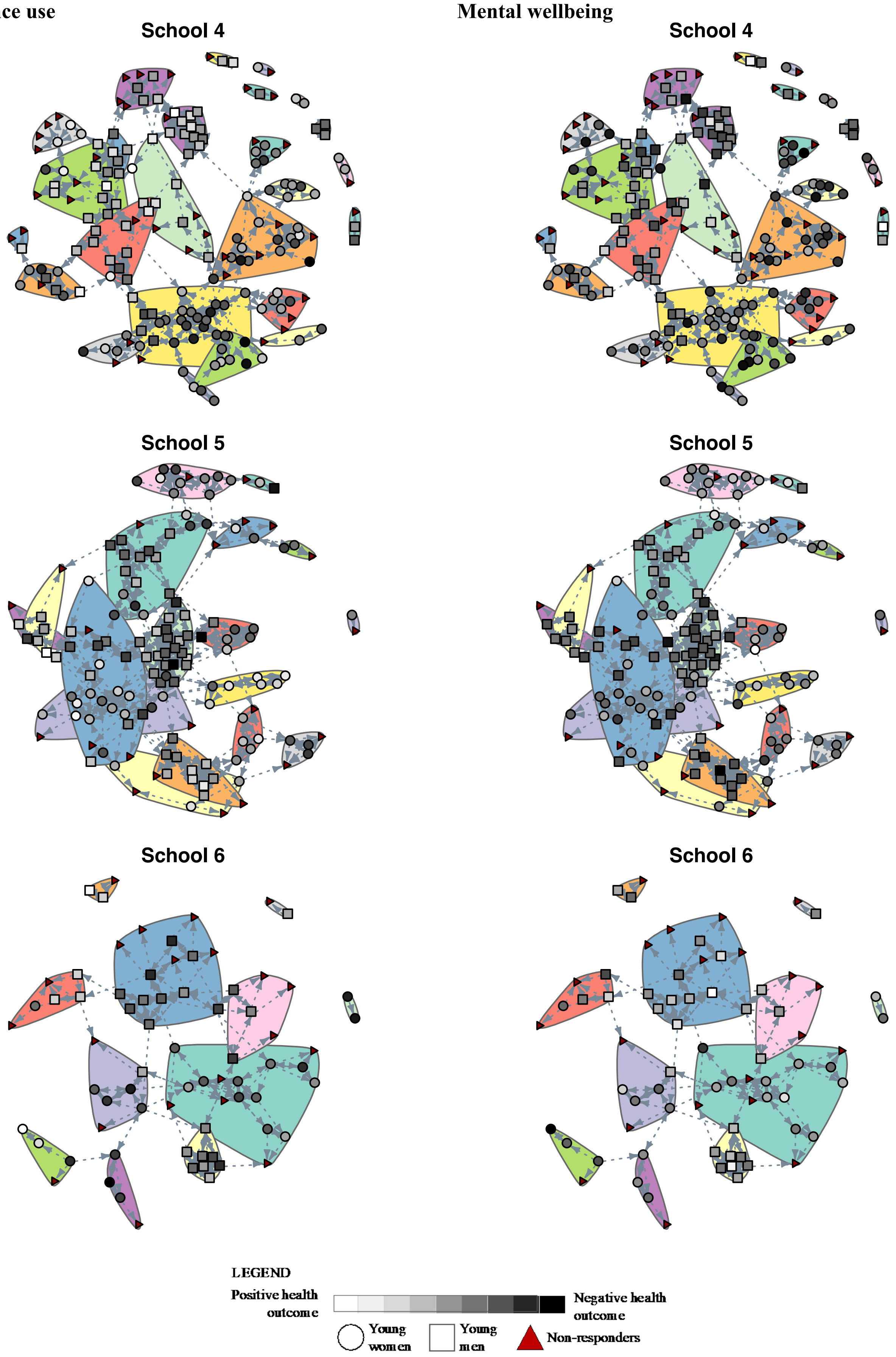

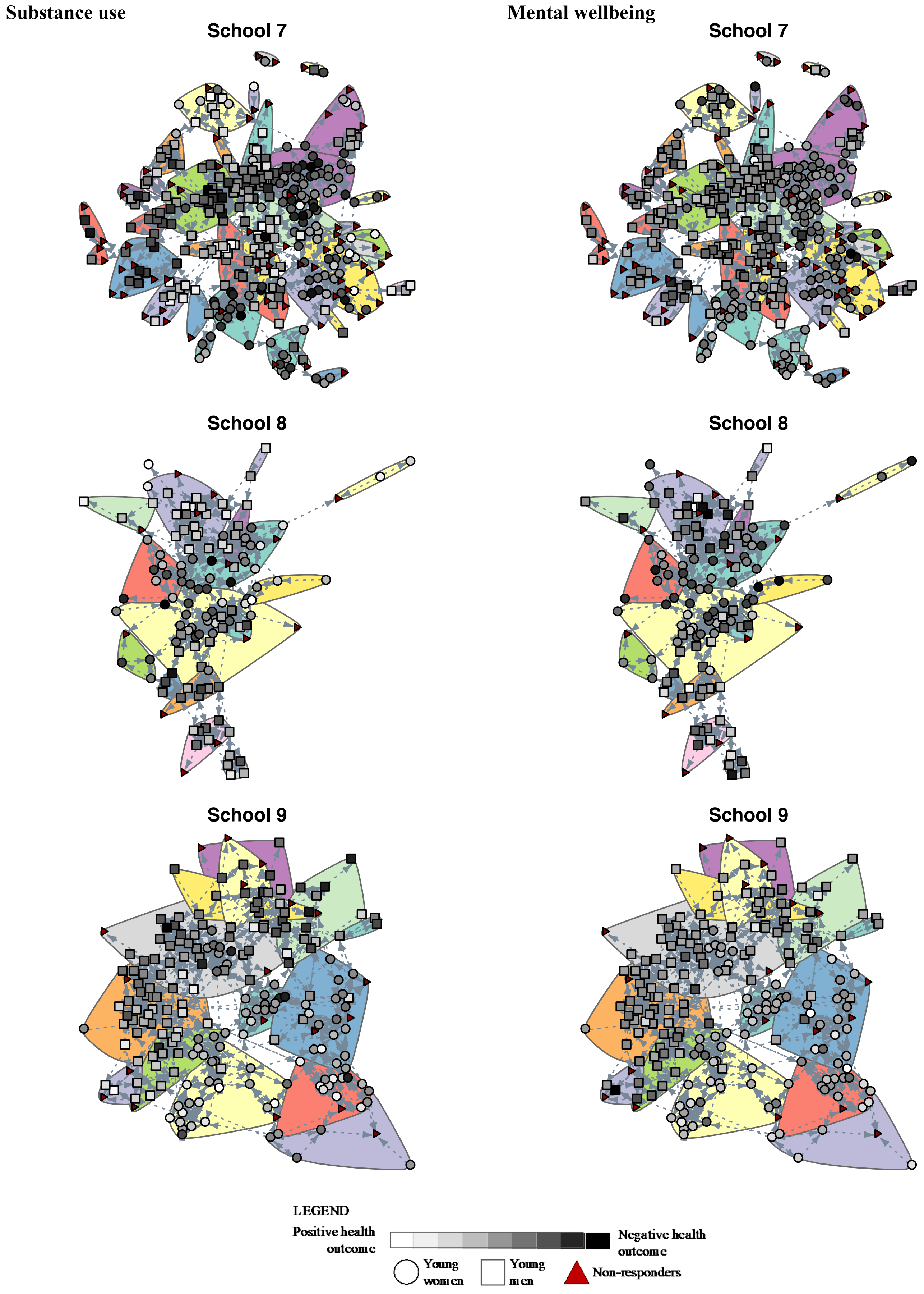
Substance use
Mental wellbeing
School 7
School 7
School 8
School 8
School 9
School 9
LEGEND
Positive health outcome
Negative health outcome
Young women
Young men
Non-responders

Substance use

School 10

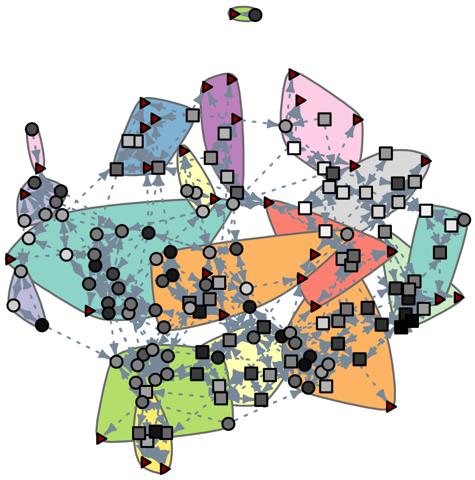

School 11

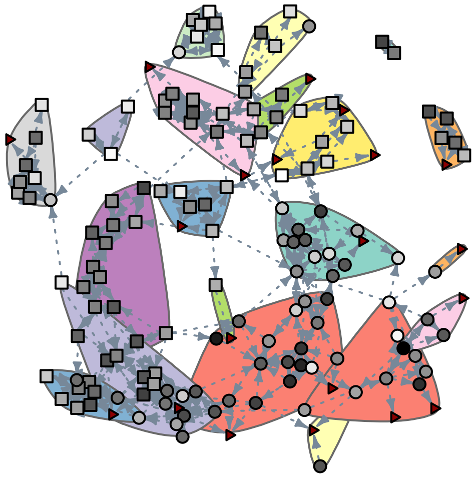

School 12

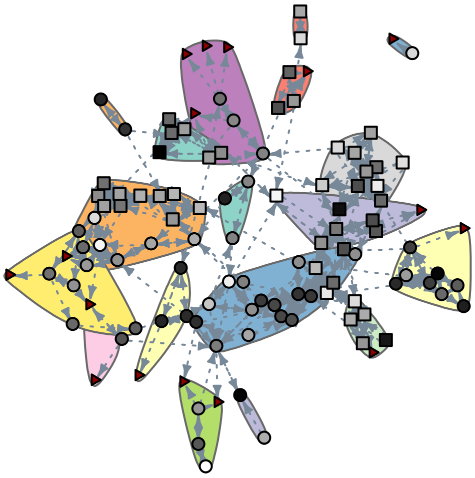

Mental wellbeing

School 10

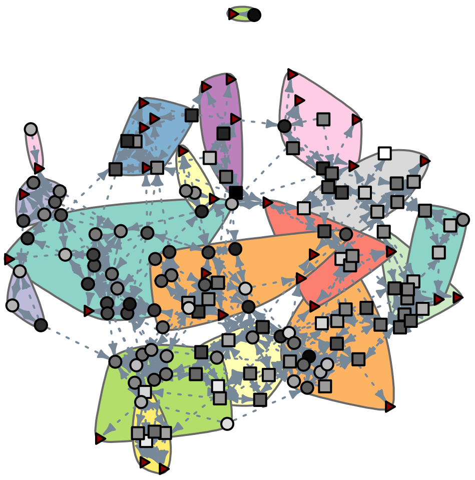

School 11

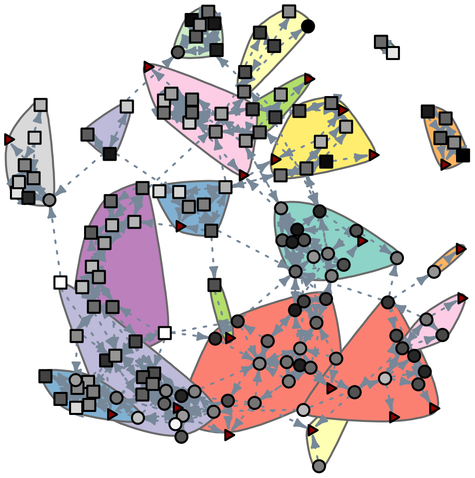

School 12

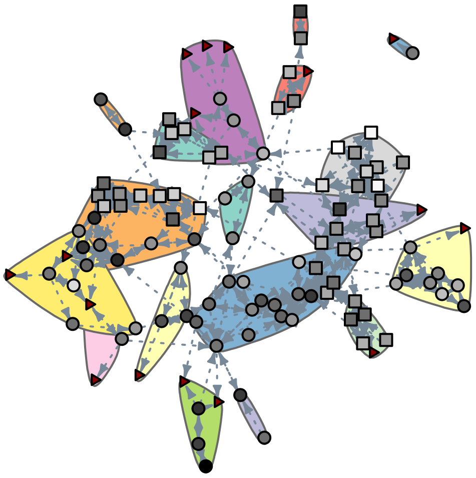

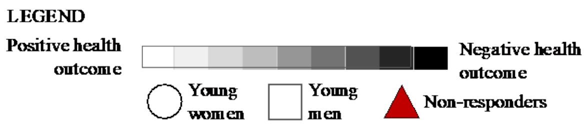

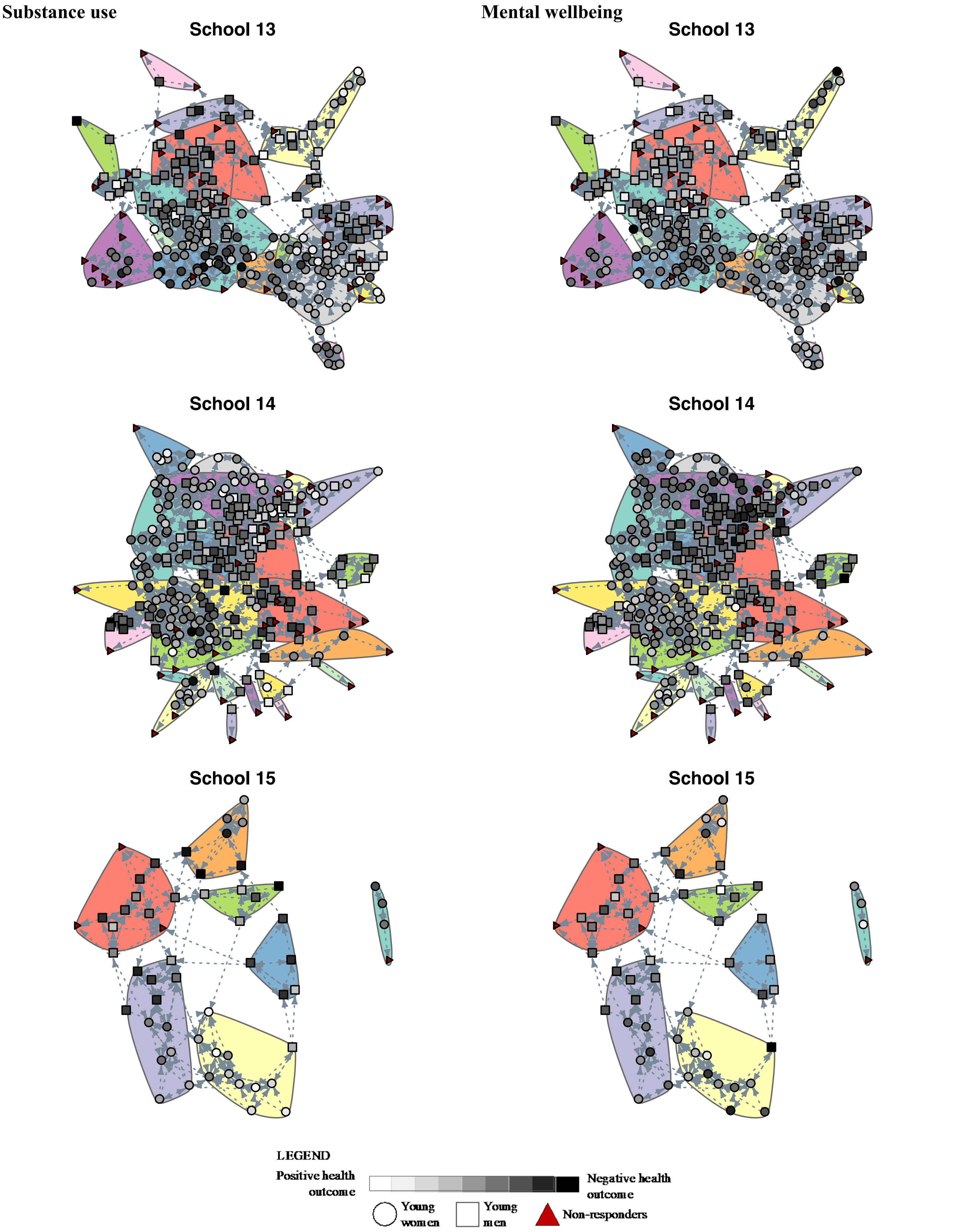
Substance use
Mental wellbeing
School 13
School 13
School 14
School 14
School 15
School 15
LEGEND
Positive health outcome
Negative health outcome
Young women
Young men
Non-responders

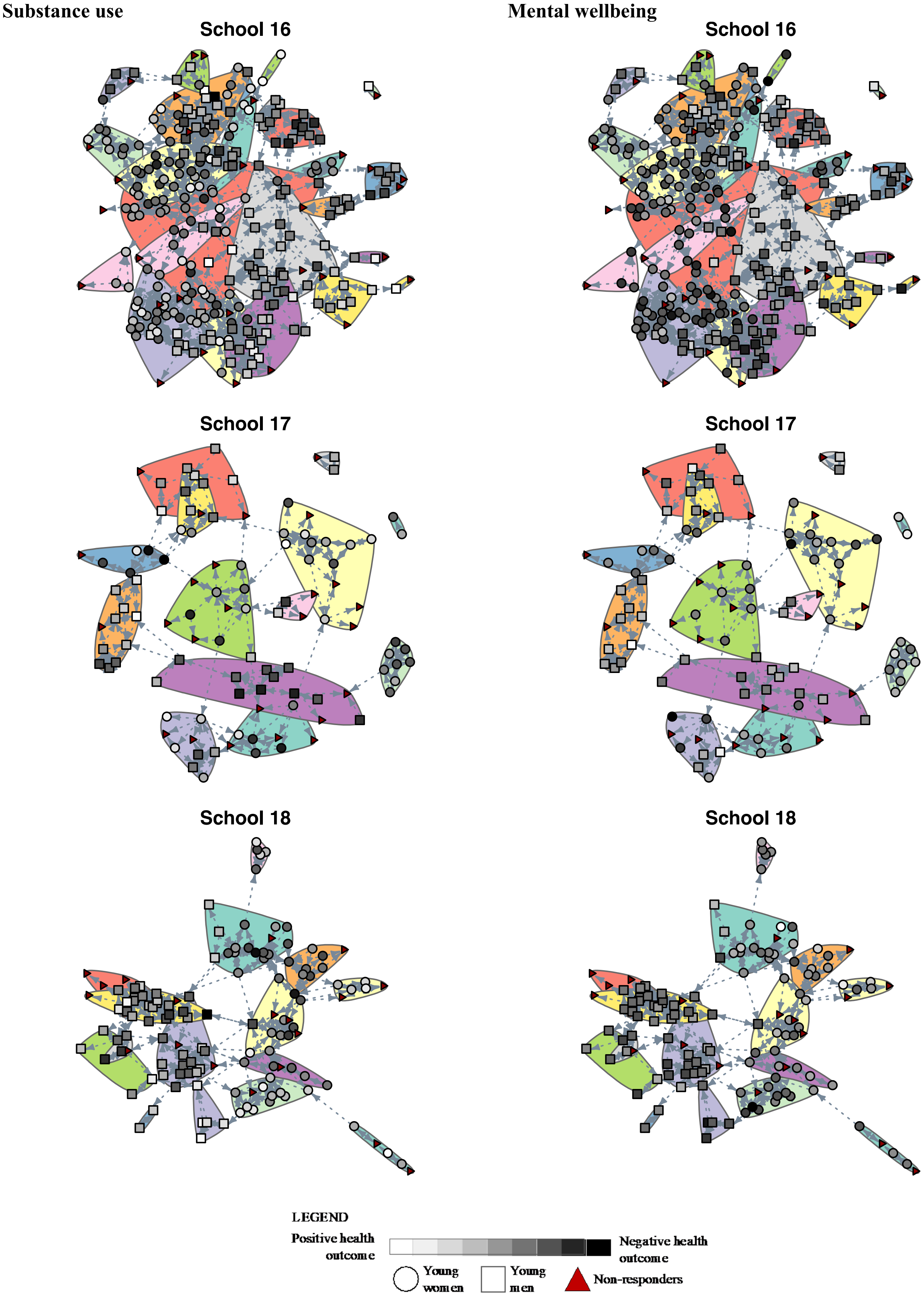
Substance use
Mental wellbeing
School 16
School 16
School 17
School 17
School 18
School 18
LEGEND
Positive health outcome
Negative health outcome
Young women
Young men
Non-responders

Substance use **Mental wellbeing**

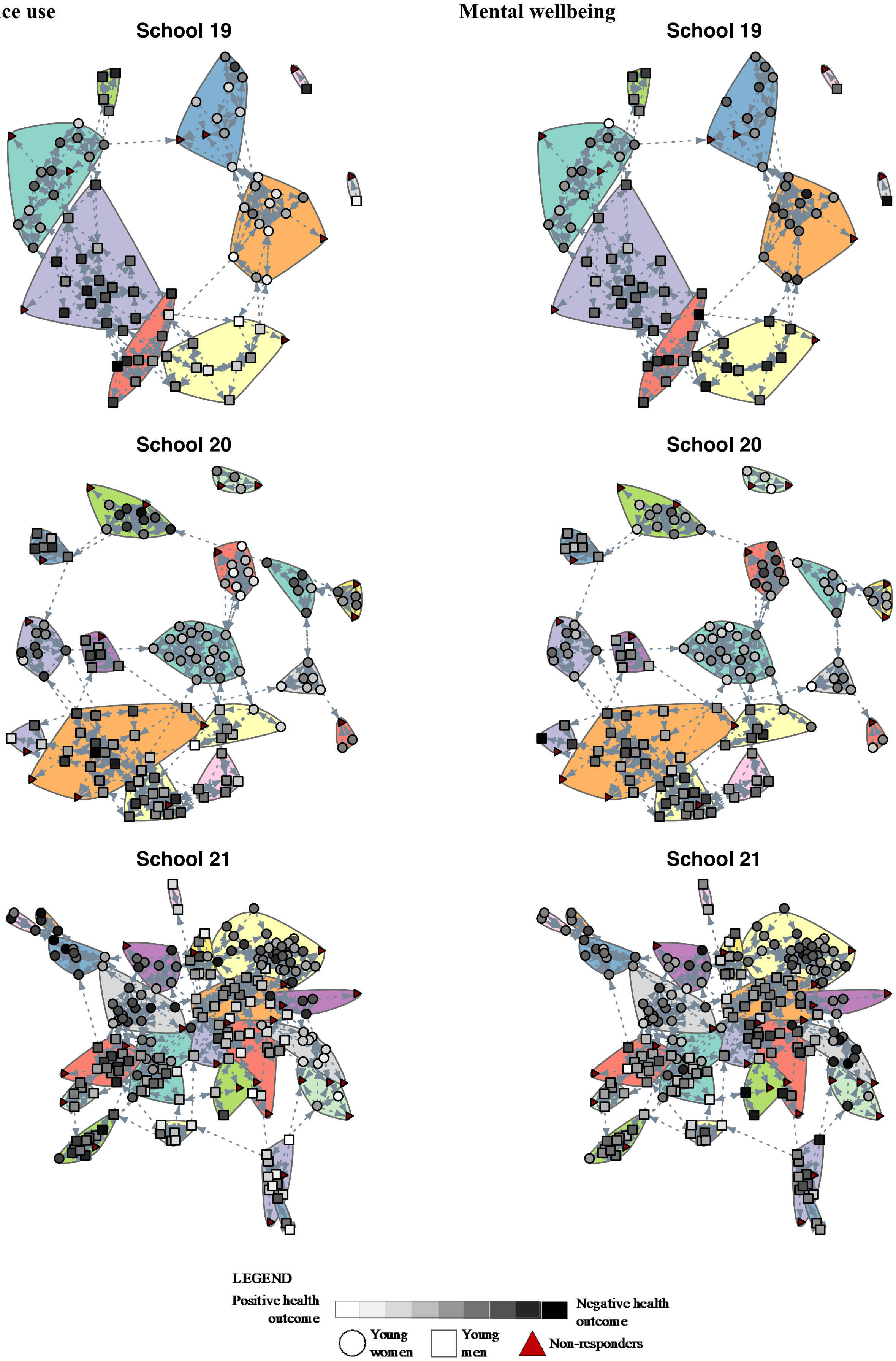

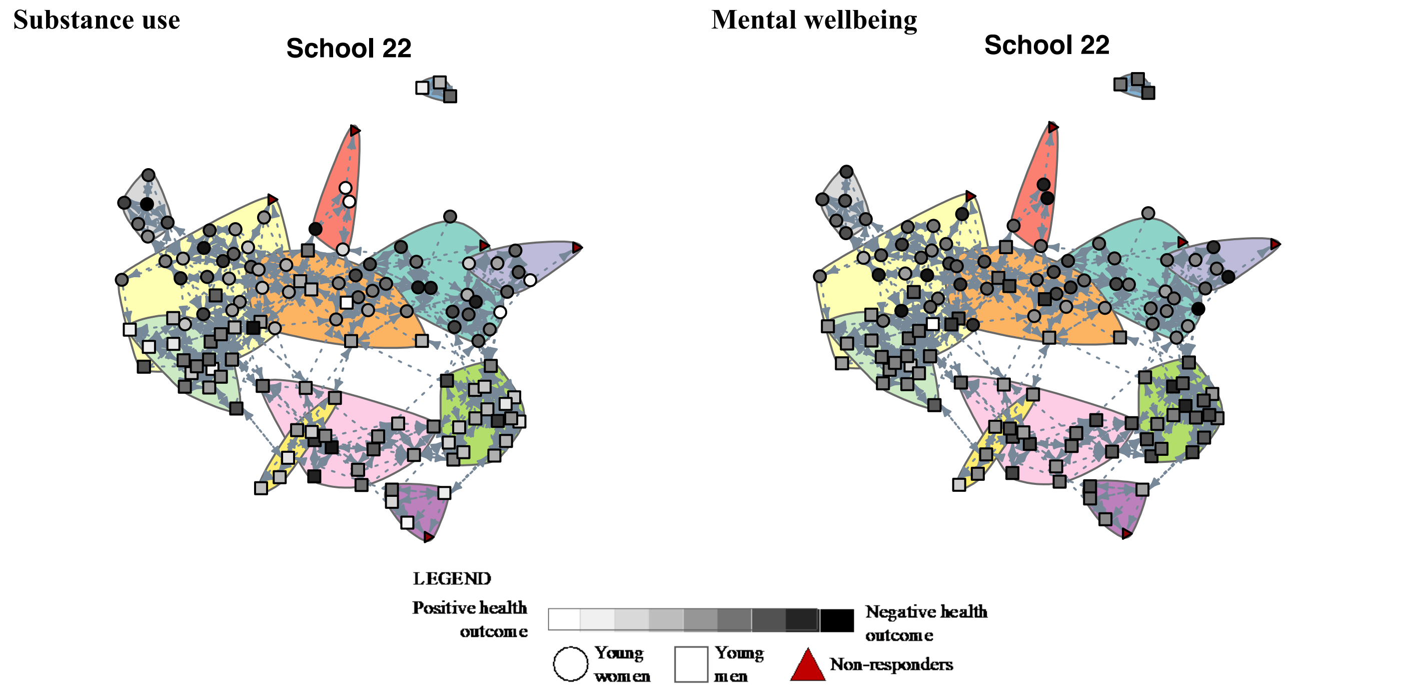

Fig S22. Communities found with Walktrap method in 22 schools and individual health outcomes, Substance Use (left) and Mental Wellbeing (right).

Codes for the analyses reported in the main manuscript are available on GitHub (https://github.com/Srebrenka/GDMs-and-health).

9 References

Batagelj, V., Ferligoj, A., & Doreian, P. (1992). Direct and indirect methods for structural equivalence. *Social networks*, 14(1-2), 63-90.

Benjamini, Y., & Yekutieli, D. (2001). The control of the false discovery rate in multiple testing under dependency. *Annals of Statistics*, 29, 1165–1188.

Blondel, V. D., Guillaume, J. L., Lambiotte, R., & Lefebvre, E. (2008). Fast unfolding of communities in large networks. *Journal of statistical mechanics: theory and experiment*, 2008(10), P10008.

Clauset, A., Newman, M. E., & Moore, C. (2004). Finding community structure in very large networks. *Physical review E,* 70(6), 066111.

Csardi, G., & Nepusz, T. (2006). The igraph software package for complex network research. *InterJournal, complex systems*, 1695(5), 1-9.

Davis, J. A., & Leinhardt, S. (1967). The structure of positive interpersonal relations in small groups.

Dewey, M. (2022). Introduction to the metap package. URL: https://cran.r-project.org/web/packages/metap/vignettes/metap.pdf (Retrieved August 2022)

DiStefano, C., Zhu, M., & Mindrila, D. (2009). Understanding and using factor scores: Considerations for the applied researcher. Practical Assessment, Research, and Evaluation, 14(1), 20.

Freeman, L. C. (1978). Centrality in social networks conceptual clarification. *Social networks*, 1(3), 215-239.

González, M. C., Herrmann, H. J., Kertész, J., & Vicsek, T. (2007). Community structure and ethnic preferences in school friendship networks. *Physica A: Statistical mechanics and its applications*, 379(1), 307-316.

Hawe, P., Webster, C., & Shiell, A. (2004). A glossary of terms for navigating the field of social network analysis. *Journal of Epidemiology & Community Health*, 58(12), 971-975.

Hennig, C., & Imports, M. A. S. S. (2015). Package 'fpc'. Available at: Available at: https://cran. r-project. org/web/packages/fpc/index. htmlENT91.

Kolaczyk, E. D., & Csárdi, G. (2014). *Statistical analysis of network data with R* (Vol. 65). New York: Springer.

Lange, J. (2021). CliquePercolation: An R Package for conducting and visualizing results of the clique percolation network community detection algorithm. *Journal of Open Source Software*, 6(62), 3210.

Leger, J. B. (2016). Blockmodels: A R-package for estimating in Latent Block Model and Stochastic Block Model, with various probability functions, with or without covariates. arXiv preprint arXiv:1602.07587.

Lüdecke, D., Ben-Shachar, M. S., Patil, I., Waggoner, P., & Makowski, D. (2021). performance: An R package for assessment, comparison and testing of statistical models. *Journal of Open Source Software*, 6(60).

Newman, M. E., & Girvan, M. (2004). Finding and evaluating community structure in networks. *Physical review E*, 69(2), 026113.

Palla, G., Derényi, I., Farkas, I., & Vicsek, T. (2005). Uncovering the overlapping community structure of complex networks in nature and society. *Nature*, 435, 814–818. https://doi.org/10.1038/nature03607

Pons, P., & Latapy, M. (2005). Computing communities in large networks using random walks. In *International symposium on computer and information sciences* (pp. 284-293). Springer, Berlin, Heidelberg.

Raghavan, U. N., Albert, R., & Kumara, S. (2007). Near linear time algorithm to detect community structures in large-scale networks. *Physical review E*, 76(3), 036106.
Rosvall, M., & Bergstrom, C. T. (2007). Maps of information flow reveal community structure in complex networks. arXiv preprint physics.soc-ph/0707.0609.
Rosvall, M., Axelsson, D., & Bergstrom, C. T. (2009). The map equation. *The European Physical Journal Special Topics*, 178(1), 13-23.
Traag, V. A., Waltman, L., & Van Eck, N. J. (2019). From Louvain to Leiden: guaranteeing well-connected communities. *Scientific reports*, 9(1), 1-12.
Van Buuren, S., & Groothuis-Oudshoorn, K. (2011). mice: Multivariate imputation by chained equations in R. *Journal of statistical software*, 45, 1-67.
